# Adhesion-derived condensates control component availability to regulate adhesion dynamics

**DOI:** 10.1038/s41467-026-74001-3

**Published:** 2026-06-05

**Authors:** Michal Dibus, Megan R. Chastney, Giray Enkavi, Gautier Follain, Omkar Joshi, Ilpo Vattulainen, Johanna Ivaska

**Affiliations:** 1 Turku Bioscience Centre, University of Turku and Åbo Akademi University, Turku, Finland; 2 InFLAMES Research Flagship, University of Turku, Turku, Finland; 3 Department of Physics, University of Helsinki, Helsinki, Finland; 4 Faculty of Science and Engineering, Cell Biology, Åbo Akademi University, Turku, Finland; 5 Turku Collegium for Science, Medicine and Technology, TCSMT, University of Turku, Turku, Finland; 6 Department of Life Technologies, University of Turku, Turku, Finland; 7 Western Finnish Cancer Center (FICAN West), University of Turku, Turku, Finland; 8 Foundation for the Finnish Cancer Institute, Helsinki, Finland

**Keywords:** Cell adhesion, Cell migration, Actin

## Abstract

Integrin adhesion complexes mediate dynamic cell-matrix interactions to regulate cell adhesion, migration and invasion. While increasing evidence suggests that adhesion components can undergo liquid-liquid phase separation, the functional relevance of adhesion-derived condensates remains elusive. Here, we demonstrate that tensin 1 (TNS1), a multidomain adaptor protein linking active integrins with the actin cytoskeleton, forms condensates in cells to regulate adhesion component availability. We show that endogenous TNS1 condensates are formed upon focal adhesion disassembly or limited integrin-extracellular matrix engagement, acting as reversible reservoirs for unphosphorylated adhesion proteins. We identify the TNS1 intrinsically disordered region as governing TNS1 condensation and condensate biophysical properties. We further demonstrate a negative regulatory role for phosphorylation on condensate assembly upon stress-responsive kinase activation. Finally, we confirm the functional effects of TNS1 condensation on adhesion dynamics and cell migration, identifying a feedback loop acutely regulating kinase-mediated adhesion turnover upon integrin activation.

## Abstract (web summary)

Integrin adhesions allow dynamic cell-matrix interactions to regulate cell behaviour. Here, the authors reveal that tensin 1, a multidomain adaptor protein, forms condensates in cells that act as reversible reservoirs for adhesion proteins.

## Introduction

Integrin adhesion complexes mediate cell interactions with extracellular matrix (ECM) components and are crucial in regulating various cellular processes, including cell survival and migration. At the onset of adhesion, small and short-lived nascent adhesions are formed that can further mature into focal adhesions (FAs) as a result of changes in protein conformation, molecular composition and actomyosin-mediated contractility^1,2^. Since FAs represent dynamic multi-protein assemblies^3^, rapid recruitment of individual FA components must be tightly controlled. While the nanoscale molecular composition of FAs is relatively well understood^4^, mechanisms governing FA component availability to facilitate their rapid recruitment at adhesion sites remain elusive. In addition, there is increasing evidence suggesting liquid-like FA organisation^5–7^, prompting further studies into how liquid-like properties of individual FA components influence their regulation and dynamics^8^.

Liquid-liquid phase separation is a biophysical process in which biomolecules spontaneously demix from solution to form condensates, whose properties are shaped by the surrounding physicochemical environment^9^. It is well-established that (nano-scale) phase separation drives macromolecule compartmentalisation in cells, resulting in biogenesis of membraneless organelles, such as processing bodies (P-bodies), stress granules and nuclear bodies^10^. In addition, recent studies have highlighted the role of biomolecular condensates in regulating diverse array of signalling events and cellular processes across the kingdoms of life, revolutionising the way we understand them. For instance, condensation of the tight junction protein ZO-1 at cell membrane interfaces has been proven essential for the assembly of tight-junction belts in polarised epithelial tissues^6,11,12^. Similarly, phase separation of the WNK1 kinase has been shown to play a crucial role in cellular response to hyperosmotic stress^13^. Finally, in plants, FLOE1 condensates modulate seed germination in response to unfavourable environmental conditions^14^.

A number of recent studies reported key nascent and FA components to form biomolecular condensates in vitro and in cells, regulating their localisation and function^5,7,15–20^. However, while these works provide valuable insights into FA-assembly mechanisms, many conclusions are drawn from in vitro reconstitutions of purified proteins, omitting the complexity of the cellular environment. The large multidomain tensin family members (tensin 1–3; TNS1–3) are key components of adhesion complexes and exert their functions by linking the actin cytoskeleton with the ECM through direct and indirect interactions with integrin heterodimers^21,22^. The shorter, oncogenic tensin 4 acts as a promigratory protein supporting growth-factor-receptor-mediated malignant processes^23,24^. Work from us and others has implicated TNS1 in maintaining active integrin pools and adhesion reinforcement^25–28^ and facilitating tension-dependent FA maturation into centrally localised fibrillar adhesions^29–32^. Furthermore, the importance of TNS1 dynamics in regulating integrin adhesions is exemplified in vascular endotoxemia, where rapid recruitment of TNS1 reinforces FAs, ultimately leading to increased vascular leakage^33^. Although key roles of TNS1 in FA regulation have been demonstrated across species^21,25^, the mechanisms underlying TNS1 dynamics in these processes remain poorly understood.

In this work, we provide evidence that TNS1 forms functional, endogenous protein condensates in a cellular context. As such, in addition to canonical roles in FAs, TNS1 selectively microcompartmentalises unphosphorylated adhesion components into reversible cytoplasmic reservoirs in response to different stimuli. We further identified regions critical for driving TNS1 condensation and established their significance in regulating condensate properties and TNS1-mediated biological functions. Finally, we demonstrated the regulatory role of TNS1 phosphorylation in condensate disassembly and control of FA dynamics.

## Results

### TNS1 forms biomolecular condensates in cells

**Fig. 1:**
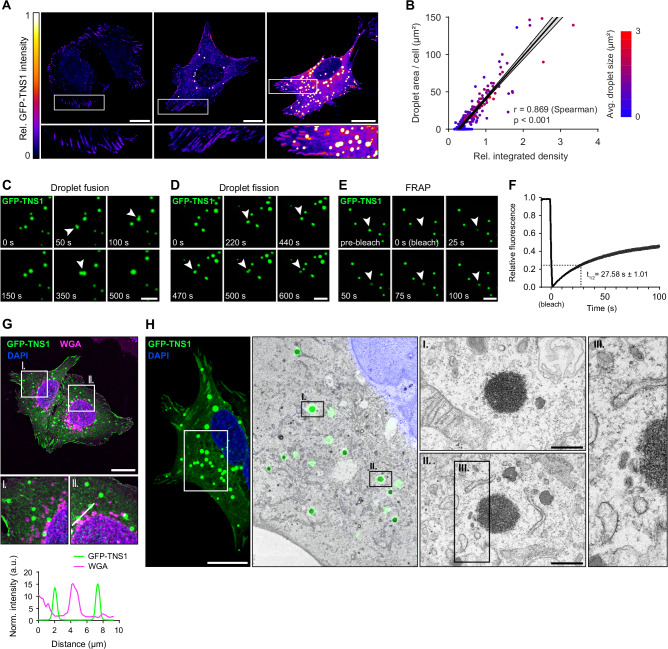
TNS1 undergoes phase separation in a concentration-dependent manner. **A** Representative confocal images of U2OS cells with varying expression of GFP-TNS1. Scale bars 20 μm. **B** Correlation between relative expression of GFP-TNS1 in U2OS cells and the total condensate area per cell, represented as linear regression with 95% confidence intervals. Colour-coded data points represent average condensate size in each of the quantified cells. Quantified from *n* = 189 cells across 2 biological replicates. **C** Representative confocal time-lapse images of GFP-TNS1 condensate fusion (indicated by arrowheads) in U2OS cells. Cells were imaged for 10 min at 10 s intervals. Scale bar 5 μm. **D** Representative confocal time-lapse images of GFP-TNS1 condensate relaxation (indicated by arrowheads) in U2OS cells. Cells were imaged for 10 min at 10 s intervals. Scale bar 5 μm. **E** Representative images from FRAP experiments. After bleaching one GFP-TNS1 condensate per cell, cells were imaged for 100 s at 1 s intervals. Bleached condensate is indicated by the arrowhead. Scale bar 5 μm. **F** Average FRAP curve from measurement of *n* = 34 condensates plotted with standard error of the mean (SEM). Half-life of maximum recovery (*t*_½_) with SEM is indicated. **G** Representative confocal images of U2OS cells expressing GFP-TNS1, stained with fluorescently-conjugated wheat germ agglutinin (WGA) and DAPI. Intensity profile plot depicts relative GFP-TNS1 and WGA intensities along the white line indicated in closeup II. Scale bar 20 μm. a.u. arbitrary units. **H** Representative images from correlative light-electron microscopy of GFP-TNS1 condensates in U2OS cells. Scale bar on confocal image 20 μm, scale bars on electron micrographs 500 nm.

In our previous studies focusing on the role of TNS1 in metabolism-mediated regulation of integrin activity^29^, we noted the formation of TNS1 biomolecular condensates similar to those observed in phase-separated proteins. To characterise these condensates and investigate whether TNS1 can undergo phase separation, we generated a U2OS (human osteosarcoma) cell line with inducible GFP-TNS1 expression. Analysis of individual cells with varying TNS1 expression levels revealed a strong positive correlation between TNS1 expression and the total TNS1 condensate area in individual cells (Fig. 1A, B), suggesting that TNS1 condensation is concentration-dependent. We observed a similar positive correlation in human umbilical vein endothelial cells (HUVECs) expressing GFP-TNS1, confirming that TNS1 condensation is not specific to the U2OS cell line (Supplementary Fig. 1A, B). We next focused on the dynamics of GFP-TNS1 condensates in living U2OS cells and confirmed that they are able to both fuse (Fig. 1C and Supplementary Video 1) and split into smaller ones (Fig. 1D and Supplementary Video 2). We also observed TNS1 condensate formation during FA disassembly (Supplementary Fig. 1C and Supplementary Video 3). While condensates proximal to FAs exhibited very limited mobility, more distal condensates exhibited dynamic movement in the cytosol (Supplementary Fig. 1D–F and Supplementary Video 4). Using fluorescence recovery after photobleaching (FRAP), we also demonstrated rapid protein turnover (ca. 49% recovery, half-life 27.58 s ± 1.01 s) between the GFP-TNS1 condensates and the soluble pool of the protein (Fig. 1E, F and Supplementary Video 5). Finally, one of the major features of biomolecular condensates is the absence of membranes around these structures. To evaluate this, we first stained the GFP-TNS1-expressing cells with fluorescently-conjugated wheat germ agglutinin (WGA) that binds to glycoproteins on the nuclear, endosomal and plasma membranes. WGA staining showed no overlap with GFP-TNS1 condensates, confirming that they are not a part of the endosomal compartment (Fig. 1G). However, given the limitations of WGA staining, we further employed correlative light electron microscopy (CLEM) to confirm the absence of membrane around TNS1 condensates. Electron micrographs correlated with immunofluorescence (IF) images revealed the absence of any discernible membrane around the GFP-TNS1 condensates, in contrast to the adjacent membrane-limited organelles, such as mitochondria, endoplasmic reticulum or vesicles (Fig. 1H, annotated micrograph in Supplementary Fig. 1G). Moreover, the condensates appeared as electron-dense granules similar to other phase-separated cellular structures, such as Cajal bodies^34^, P-bodies^35^ and stress-granules^36,37^. Taken together, in line with the accepted guidelines^38^, we provide strong evidence that TNS1 condensates are dynamic membraneless particles able to exchange material with the surrounding cytoplasm, confirming that TNS1 can undergo phase separation in a concentration-dependent manner *in cellulo*.

### Endogenous TNS1 condensation is controlled by ECM ligand availability and actomyosin contractility

**Fig. 2:**
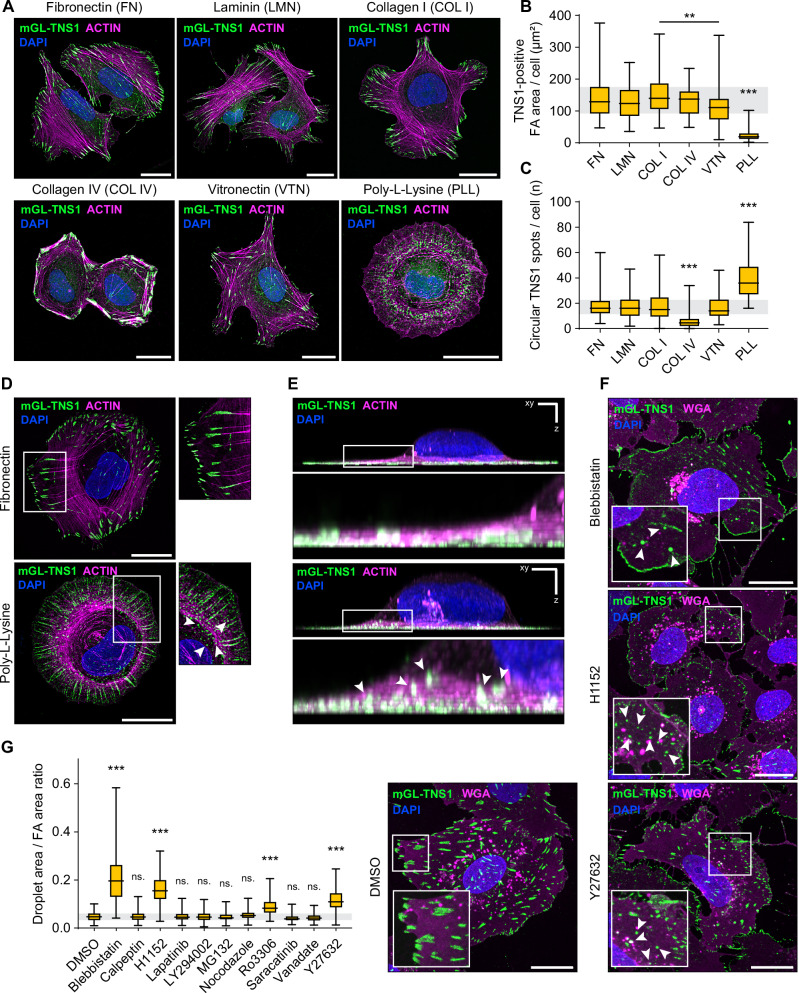
Endogenous TNS1 condensation is controlled by integrin ligand availability and actomyosin contractility. **A** Representative confocal images of U2OS cells with endogenously tagged TNS1 (mGreenLantern TNS1, mGL-TNS1) seeded for 3 h on different integrin ligands or poly-L-lysine (PLL) as indicated. Images have been deconvolved using Huygens Professional (SVI). Scale bars 20 μm. **B** Quantification of FA area per cell from *n* = 56 (FN), 61 (LMN), 97 (COL I), 84 (COL IV), 74 (VTN) and 55 (PLL) cells from two (FN, LMN, COL IV, VTN, PLL) to three (COL I) biological replicates. FAs were quantified as TNS1-positive spots with circularity 0–0.9. **C** Quantification of the number of circular TNS1 spots (circularity 0.9–1) per cell from *n* = 56 (FN), 61 (LMN), 97 (COL I), 84 (COL IV), 74 (VTN) and 55 (PLL) cells per condition from two (FN, LMN, COL IV, VTN, PLL) to three (COL I) biological replicates. **D** Representative confocal images of U2OS cells with mGL-TNS1 seeded either on fibronectin or poly-L-lysine and stained for F-actin and DAPI. Some of the mGL-TNS1 condensates are indicated with arrows. Images have been deconvolved using Huygens Professional (SVI). Scale bar 20 μm. **E** Maximum intensity projection orthogonal rendering of cells from Fig. 2D. mGL-TNS1 condensates are indicated by arrows. Projection was performed in Huygens Professional (SVI). Scale bar 5  μm. **F** Representative confocal images of mGL-TNS1 U2OS cells treated with DMSO, blebbistatin, H1152 and Y27632 inhibitors (both ROCK inhibitors). Cells were stained with WGA and DAPI. mGL-TNS1 condensates are indicated with arrows. Scale bars 20 μm. **G** Quantification of the mGL-TNS1 droplet area and FA area ratio from *n* = 110 (DMSO), 118 (blebbistatin), 107 (calpeptin), 119 (H1152), 116 (lapatinib), 116 (LY294002), 101 (MG132), 110 (nocodazole), 109 (Ro3306), 112 (saracatinib), 108 (vanadate) and 124 (Y27632) cells from three biological replicates treated with the indicated compounds. Representative images are shown in Fig. 2F and Supplementary Fig. 3A. Statistical analysis was performed using the Kruskal–Wallis test with Dunn’s multiple comparisons test (**B**, **C**, **G**). Unless indicated, the statistical significance level is valid for comparison with each of the conditions (**B**, **C**) or with DMSO (**G**). *p* < 0.001 (***). Source data, including exact *p*-values are provided as a Source data file.

To study TNS1 condensation at endogenous protein levels, we generated a U2OS cell line with endogenous TNS1 tagged with the bright monomeric green fluorescent protein, mGreenLantern (mGL)^39^ (Supplementary Fig. 2A–D). To examine the response of TNS1-containing FAs to different ECM stimuli, we seeded these cells on glass coverslips coated either with various integrin-binding ECM ligands or with poly-L-Lysine (PLL) to mimic conditions with low ligand availability. While we observed only minor differences in FA area between cells seeded on fibronectin (FN), laminin (LMN), collagen I (COL I), collagen IV (COL IV) and vitronectin (VTN), FA area was significantly reduced in cells seeded on PLL (Fig. 2A, B). Interestingly, this correlated with a substantial increase in the amount of small, circular mGL-positive spots likely corresponding to TNS1 condensates and nascent adhesions (Fig. 2C). In contrast, cells seeded on COL IV exhibited a significant reduction of circular TNS1 spots (Fig. 2C), consistent with the observed changes in FA morphology reminiscent of large and stable cornerstone FAs, previously described in human pluripotent stem cells^40^. To further verify whether some of the TNS1 spots in cells seeded on PLL represent TNS1 condensates, we generated maximum intensity orthogonal projections of cells seeded either on FN or PLL (Fig. 2D, E). While in cells seeded on FN, all endogenous TNS1 was localised to the basal plane corresponding to FAs, in cells seeded on PLL, several mGL-TNS1 spots were observed in higher cell planes (Fig. 2E), indicating TNS1 forms condensates at endogenous levels in response to limited integrin-ECM engagement. To extend our observations further to a 3D environment, we embedded the cells into a 3D fibrillar collagen I matrix. After 3 h, the cells had already formed prominent protrusions with TNS1 adhesions anchored on the underlying collagen fibres (Supplementary Fig. 2E, left panel). Interestingly, in less protruding cells, we consistently observed several cytoplasmic TNS1 condensates (Supplementary Fig. 2E, right panels), suggesting their potential role in the local buffering of TNS1 amounts also in 3D conditions. We then performed a small screen using inhibitors of signalling pathways with established roles in regulating FA dynamics and examined their effect on TNS1 condensate formation in cells with endogenously tagged TNS1. While most inhibitors targeting canonical pathways downstream of integrin receptor activation and protein stability did not have a significant effect on TNS1 condensate formation (Fig. 2F, G and Supplementary Fig. 3A), treatment with compounds affecting actomyosin contractility and FA stability (ROCK inhibitors H1152, Y27632; Myosin-II inhibitor Blebbistatin; CDK1 inhibitor Ro3306) led to a markedly increased amount of TNS1 condensates, as a result of local increase in TNS1 concentration after FA disassembly (Fig. 2F, G). To further address whether TNS1 condensation at endogenous levels is responsive to mechanosensitive cues, we seeded cells on FN-coated hydrogels of varying stiffness (0.5, 2 and 20 kPa), including glass coverslips as a control. As expected, and in line with our previous work on U2OS cells^41^, we observed a significant increase in cell area with increasing substrate stiffness (Supplementary Fig. 3B, C), which correlated with increased mGL-TNS1-positive FA area in these cells (Supplementary Fig. 3D). However, the cells seeded on softer substrates (0.5 and 2 kPa) did not exhibit increased numbers of mGL-TNS1 circular spots when compared to cells seeded on stiffer substrate (20 kPa) or glass as would have been expected if TNS1 condensation was driven by mechanosensitive cues from substrate stiffness (Supplementary Fig. 3E, F). Taken together, our results suggest that endogenous TNS1 condensates are formed in response to limited integrin ligand availability and decreased actomyosin contractility, and their formation could play a role in microcompartmentalisation and buffering of local protein concentration.

### TNS1 condensates selectively recruit unphosphorylated FA proteins

**Fig. 3:**
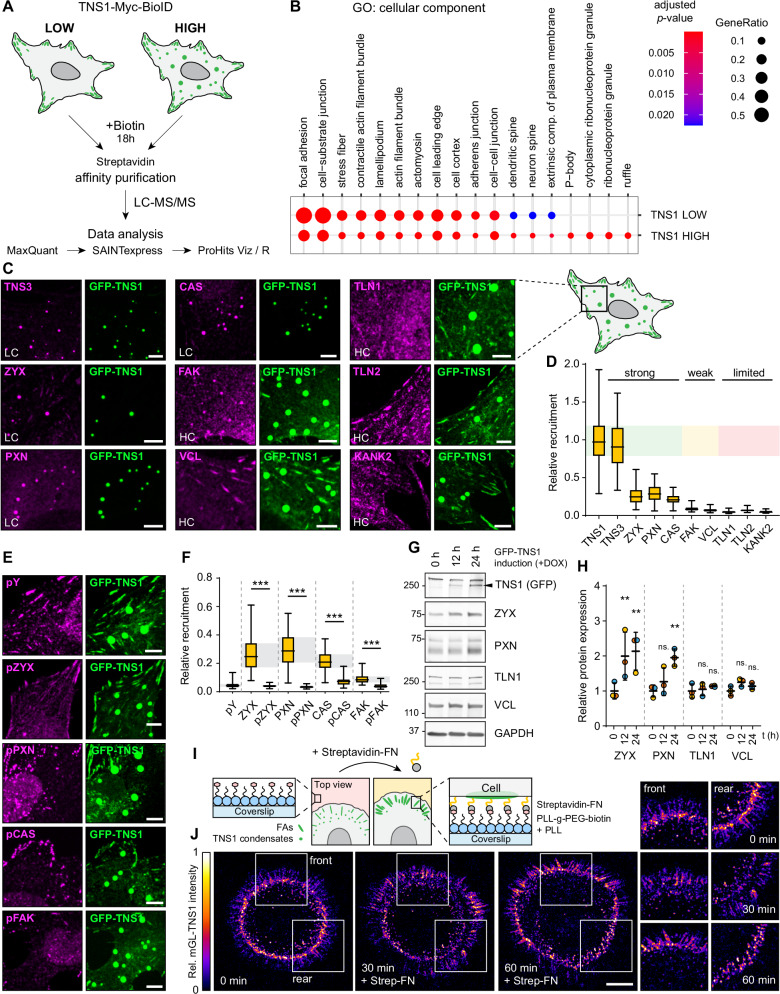
TNS1 condensates act as a selective and reversible reservoir for unphosphorylated FA proteins. **A** Schematic representation of the BioID workflow. **B** Gene ontology (GO) cellular component enrichment analysis of proteins identified in TNS1 BioID screen (one-sided Fisher’s exact test with Benjamini–Hochberg adjustment). **C** Closeups of GFP-TNS1 condensates in U2OS cells (representative images in Supplementary Fig. 5A) stained with the indicated antibodies. Cells were seeded overnight on FN-coated coverslips. LC low contrast, HC high contrast. Scale bars 5 μm. **D** Quantification of relative recruitment of indicated FA components into cytoplasmic GFP-TNS1 condensates (ratio of integrated density in the condensates and total integrated density (excluding the nuclear signal in all channels) normalised on total condensate area in each cell). Quantification was performed from *n* = 682 (TNS1), 55 (TNS3), 48 (ZYX), 45 (PXN), 44 (CAS), 47 (FAK), 40 (VCL), 50 (TLN1), 71 (TLN2) and 45 (KANK2) cells from two (TNS3, ZYX, PXN, CAS, FAK, VCL, TLN1, KANK2) or three (TNS1, TLN2) independent replicates. **E** Closeups of GFP-TNS1 condensates in U2OS cells (representative images in Supplementary Fig. 5B) stained with the indicated phospho-specific antibodies. Cells were seeded overnight on FN-coated coverslips. Scale bars 5 μm. **F** Quantification of the relative recruitment of the indicated proteins into cytoplasmic GFP-TNS1 condensates as in Fig. 3D. Quantification was performed from *n* = 63 (pY), 48 (ZYX), 85 (pZYX), 45 (PXN), 59 (pPXN), 44 (CAS), 55 (pCAS), 47 (FAK) and 63 (pFAK) cells from two (pY, ZYX, PXN, pPXN, CAS, pCAS, FAK, pFAK) or three (pZYX) independent experiments. Statistical analysis was performed using the Kruskal–Wallis test with Dunn’s multiple comparisons test. *p* < 0.001 (***). **G** Representative immunoblots of expression levels of indicated proteins at indicated times after GFP-TNS1 expression (doxycycline). Molecular weight markers are in kDa. **H** Quantification of relative protein expression from immunoblots in Fig. 3H, normalised to protein expression at *t* = 0 h. Quantification from *n* = 3 independent experiments, data points corresponding to individual replicates are colour-coded. Statistical analysis was performed using 2-way ANOVA with Holm–Šídák’s multiple comparison test. Indicated statistical differences are compared to protein expression at *t* = 0 h. *p* < 0.001 (***); ns. not significant. **I** Schematic representation of the dynamic release method. Cells were seeded on glass coverslips coated with a combination of PLL-g-PEG-biotin and PLL. Cell adhesion was promoted by adding streptavidin-conjugated FN to the culture medium. **J** Representative confocal time-lapse images of U2OS mGL-TNS1 cells seeded as described in Fig. 3I before (*t* = 0 min) and after (*t* = 30 min and 60 min) adding streptavidin-FN. Scale bar 10 μm. Source data, including exact *p*-values are provided as a Source data file.

To study the molecular composition of TNS1 condensates and their potential role in buffering the concentration of FA components, we employed proximity biotinylation using BioID in U2OS cells with either low or high expression of TNS1-Myc-BioID (bait; approx. 3.7-fold (±0.3) and 26.8-fold (±3.2) expression when compared to endogenous TNS1 levels, respectively; Supplementary Fig. 4A–C). While in the low-expressing cells the bait predominantly localised to FAs, higher TNS1-Myc-BioID expression induced condensate formation allowing us to study the phase separation-dependent TNS1 proximity interactome (Fig. 3A and Supplementary Fig. 4D). Statistical analysis of the BioID proteomic data using SAINTexpress^42^ identified 160 high confidence hits across 4 independent replicates (Supplementary Fig. 4E). As expected, gene ontology (GO) analysis of identified proteins revealed a significant enrichment in GO cellular component terms related to canonical TNS1 functions in linking cell adhesions with the actin cytoskeleton (Fig. 3B). To validate the results of the screen and to identify FA-associated proteins in the TNS1 condensates, we performed immunofluorescence staining against a set of FA proteins in U2OS cells with inducible GFP-TNS1 expression seeded on FN. While tensin 3 (TNS3), zyxin (ZYX), paxillin (PXN) and p130-Crk associated substrate (CAS) exhibited strong recruitment to TNS1 condensates, other FA-associated proteins such as focal adhesion kinase (FAK), vinculin (VCL), talin 1 (TLN1), talin 2 (TLN2) or KN motif and ankyrin repeat domains 2 (KANK2) showed weak to limited condensate localisation (Fig. 3C, D, uncropped representative images for each staining are shown in Supplementary Fig. 5A). To further address the recruitment specificity, we quantified the correlation between relative mean intensity of the studied proteins in individual GFP-TNS1 condensates and the condensate size. While strongly positive correlation values demonstrated that TNS3, ZYX, PXN, CAS, FAK and VCL are, although to a very different extent, specifically recruited to GFP-TNS1 condensates, low correlation values indicated that TLN1, TLN2 and KANK2 recruitment to GFP-TNS1 condensates is limited or non-detectable (Supplementary Fig. 6 and Fig. 3D), suggesting that protein recruitment to TNS1 condensates is of a selective nature. We next assessed the canonical phosphorylation status of these proteins downstream of active integrin signalling by staining with a general phospho-tyrosine (pY) antibody or antibodies recognising phosphorylated ZYX, PXN, CAS or FAK. While all these antibodies specifically recognised the respective phosphorylated proteins at FAs, the staining was completely absent from GFP-TNS1 condensates (Fig. 3E, F, uncropped representative images for each staining are shown in Supplementary Fig. 5B). To validate these results also at the endogenous level, we seeded the U2OS cells with endogenously tagged TNS1 (mGL-TNS1) on PLL and stained for either PXN or phosphorylated PXN. Indeed, while PXN did colocalise with the mGL-TNS1 spots, phosphorylated PXN localised predominantly in the belt of nascent adhesions at the cell periphery and not in TNS1 condensates (Supplementary Fig. 4F). Together, our results demonstrate that TNS1 condensates selectively recruit unphosphorylated FA components.

### TNS1 condensates act as reversible reservoirs of FA proteins

We next hypothesised that TNS1 condensates may function to buffer the local concentration of FA proteins, thereby maintaining a FA component pool readily available for rapid recruitment during FA assembly. To support this notion, we first examined whether an increase in TNS1 expression could acutely stabilise the levels of FA components by their recruitment to TNS1 condensates. To test this, we induced GFP-TNS1 expression in U2OS cells using doxycycline for either 12 or 24 h and compared the expression levels of the selected proteins to those in uninduced cells. An increase in GFP-TNS1 expression led to significantly elevated levels of ZYX and PXN that are strongly recruited into TNS1 condensates. In contrast, the levels of TLN1 and VCL, which exhibit weak/limited recruitment to TNS1 condensates, were not affected by changes in GFP-TNS1 expression (Fig. 3G, H). Real-time quantitative PCR confirmed that the increase in ZYX and PXN protein levels was not mediated by transcriptional regulation (Supplementary Fig. 7A). Furthermore, expression of GFP control in U2OS cells did not affect protein expression of any of the studied FA components (Supplementary Fig. 7B, C). These data suggest that elevated TNS1 levels are sufficient to support expression of ZYX and PXN proteins. However, TNS1 depletion (knock-out, KO) in U2OS cells did not alter the expression levels of ZYX and PXN in these cells (Supplementary Fig. 7D, E), implying that TNS1 is not necessary for their expression and their stability is expected to be regulated also by recruitment to adhesions and possibly to other adhesion-derived condensates. In addition, TNS1 expression is not required for the condensation of other FA components, such as CAS and PXN, which have previously been shown to form condensates^7,19^ (Supplementary Fig. 7F).

To assess the crosstalk between TNS1 condensates and FA assembly, we first seeded the U2OS cells with inducible expression of GFP-TNS1 on PLL, followed by acute integrin activation using MnCl_2_. Time-lapse imaging with high temporal resolution revealed that a substantial number of GFP-TNS1 condensates at the cell periphery disassembled, thereby locally increasing the availability of the adhesion components in the cytoplasmic pool, which correlated with the formation of prominent TNS1-positive adhesions in close proximity (Supplementary Fig. 7G and Supplementary Video 6). Importantly, we did not observe TNS1 condensates to directly fuse with newly forming FAs. To confirm our findings at endogenous levels, we seeded the U2OS cells with endogenously tagged TNS1 (mGL-TNS1) on PLL and acquired images before and 40 min after integrin activation. Similarly, we observed increased intensity of mGL-TNS1-positive FAs at the cell periphery coupled with disassembly of the adjacent TNS1 condensates (Supplementary Fig. 7H). To further support our observations, we next employed a modified dynamic micropattern release approach we have recently developed^43^, which enabled spatio-temporally controlled spreading of cells from non-integrin-engaging conditions to integrin ligand-coated surface (Fig. 3I). We seeded the U2OS mGL-TNS1 cells on coverslips coated with a combination of PLL-g-PEG-biotin and PLL. As expected, the cells recapitulated the phenotype of cells seeded on PLL-coated coverslips, with round morphology, a high number of mGL-TNS1 condensates and limited TNS1-positive FAs. After acquiring the initial images (*t* = 0 min), streptavidin-conjugated FN was added to the culture medium to promote integrin engagement and cell spreading. Already at *t* = 30 min, we observed the formation of new TNS1-positive FAs at the cell periphery, accompanied by the dissolution of the adjacent TNS1 condensates. This became even more pronounced at *t* = 60 min with strong mGL-TNS1 FAs forming at the leading edge and a substantial loss of TNS1 condensates in their close proximity (Fig. 3J). Interestingly, at *t* = 60 min we also observed the formation of new TNS1 condensates from disassembling FAs at the cell rear (Fig. 3J).

Taken together, our results indicate that TNS1 condensates can acutely stabilise FA components to promote adjacent FA assembly.

### P-bodies are closely associated with TNS1 condensates

The GO analysis of the BioID screen revealed a significant enrichment of genes related to P-bodies and ribonucleoprotein granules in U2OS cells with high expression of TNS1-Myc-BioID (Fig. 3B, Supplementary Figs. 4E and 8A). To examine whether TNS1 condensates recruit P-body proteins, we initially stained the GFP-TNS1 expressing U2OS cells seeded on FN for a canonical P-body component^44^ identified in the BioID screen, mRNA-decapping enzyme 1B (DCP1B). Surprisingly, DCP1B staining did not overlap with the GFP-TNS1 condensates but instead localised in their proximity (Supplementary Fig. 8B), suggesting a close association between the P-bodies and TNS1 condensates. We further stained the cells for two well-established P-body markers, enhancer of mRNA-decapping protein 3 (EDC3) and DEAD-Box Helicase 6 (DDX6). Both EDC3 and DDX6 staining recapitulated the close proximity of P-bodies and TNS1 condensates (Supplementary Fig. 8C), which was further confirmed by imaging EDC3 using super-resolved structured illumination microscopy (SR-SIM) (Supplementary Fig. 8D). In addition, we observed association of P-bodies with endogenous TNS1 condensates in U2OS cells with endogenously tagged TNS1 (mGL-TNS1) seeded on PLL (Supplementary Fig. 8E). Since P-bodies are involved in messenger RNA (mRNA) storage, translational repression and mRNA decay, and mRNA recruitment has been reported proximal to force-loaded focal adhesions^45^, we assayed the presence of mRNA in the GFP-TNS1 condensates using fluorescent in situ hybridisation (FISH) with probes targeting mRNA poly-A tails. Although the probes readily detected polyA-mRNA in the cell nucleus and cytoplasm, we did not detect any enrichment of the FISH signal in TNS1 condensates (Supplementary Fig. 8F), indicating that mRNA is either absent from TNS1 condensates or the polyA tails are not accessible for binding of the oligo-dT probe. Overall, these observations further validate the results of our proteomic data; however, the functional significance of P-body association with FA-derived condensates remains the objective of future studies.

### TNS1 condensation is mediated by its intrinsically disordered region

**Fig. 4:**
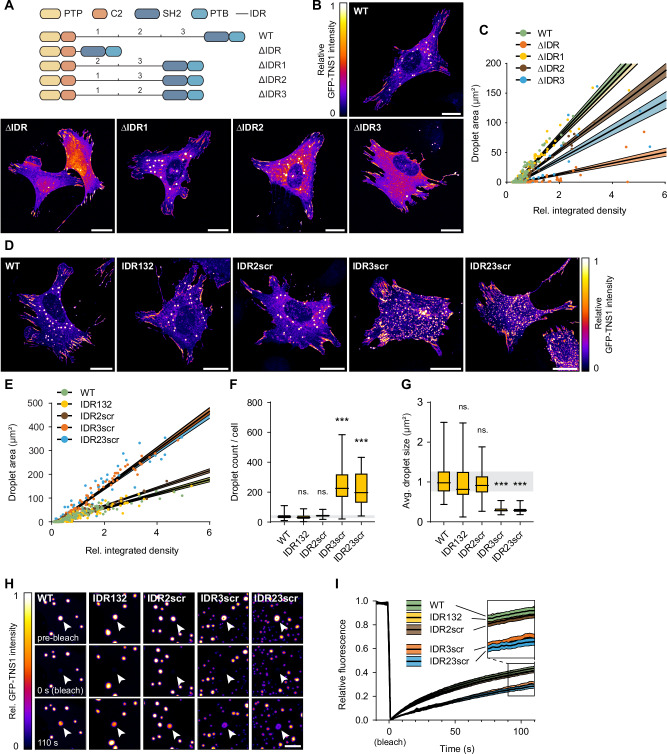
TNS1 condensation is mediated by its intrinsically disordered region. **A** Schematic representation of the domain organisation of TNS1 WT and the individual IDR deletion mutants. **B** Representative confocal images of U2OS TNS1 KO cells expressing the indicated GFP-TNS1 variants. Cells were seeded overnight on FN-coated coverslips. Scale bars 20 μm. **C** Correlation between droplet area and relative integrated density quantified from U2OS TNS1 KO cells expressing individual GFP-TNS1 variants. Quantification was performed from *n* = 114 (WT), 134 (ΔIDR), 121 (ΔIDR1), 90 (ΔIDR2) and 168 (ΔIDR3) cells. The centre line represents the fitted linear regression (mean prediction), 95% confidence bands are shown. **D** Representative confocal images of U2OS TNS1 KO cells expressing individual GFP-TNS1 variants. Cells were seeded overnight on FN-coated coverslips. Scale bars 20 μm. **E** Correlation between droplet area and relative integrated density quantified from U2OS TNS1 KO cells expressing individual GFP-TNS1 variants. Quantification was performed from *n* = 133 (WT), 137 (IDR132), 136 (IDR2scr), 137 (IDR3scr) and 137 (IDR23scr) cells. The centre line represents the fitted linear regression (mean prediction), 95% confidence bands are shown. **F** Quantification of GFP-TNS1 droplet count from *n* = 61 (WT), 64 (IDR132), 61 (IDR2scr), 46 (IDR3scr) and 48 (IDR23scr) cells per condition. **G** Quantification of GFP-TNS1 average droplet size from *n* = 61 (WT), 65 (IDR132), 62 (IDR2scr), 46 (IDR3scr) and 48 (IDR23scr) cells. **H** Representative FRAP images of individual GFP-TNS1 condensates at the indicated time points. Scale bar 5 μm. **I** Average FRAP curves from measurement of *n* = 40 (WT), 41 (IDR132), 39 (IDR2scr), 40 (IDR3scr) and 31 (IDR23scr) condensates, plotted with standard error of the mean (SEM). Statistical analysis was performed using the Kruskal–Wallis test with Dunn’s multiple comparisons test against WT (**F**, **G**). *p* < 0.001 (***); ns. not significant. Source data, including exact *p*-values are provided as a Source data file.

Protein condensation is generally mediated either by site-specific interactions (SSI) between structurally organised domains, by low complexity sequences across intrinsically disordered regions (IDRs), or by a combination of both^38,46^. TNS1 encompasses two N-terminal (protein tyrosine phosphatase, PTP; homologous protein kinase C conserved region 2, C2) and two C-terminal domains (src-homology 2 domain, SH2; phosphotyrosine binding domain, PTB) that are interconnected with a long IDR (Supplementary Fig. 9A). To discriminate between the SSI- and IDR-mediated phase transition mechanisms and to assess how individual regions contribute to TNS1 condensation, we generated a series of deletion mutants lacking each of the individual domains or the entire IDR (Supplementary Fig. 9A). Expression of the individual GFP-TNS1 mutants in U2OS TNS1 KO cells showed that deletion of any of the structurally conserved domains (ΔPTP, ΔC2, ΔSH2 or ΔPTB) did not have any substantial effect on TNS1 condensation. In contrast, deletion of the IDR (ΔIDR) resulted in an almost complete abrogation of the ability of TNS1 to phase separate (Supplementary Fig. 9B). To evaluate whether there are any specific regions within the TNS1 IDR preferentially promoting phase separation, we generated three mutants with sequential deletions of approximately a third of the IDR sequence (ΔIDR1, ΔIDR2 and ΔIDR3) (Fig. 4A) and quantified the correlation between relative expression of the individual mutants and their propensity to form condensates. While we observed no significant difference between GFP-TNS1 WT and ΔIDR1, deletion of both IDR2 and IDR3 significantly reduced TNS1 condensation, although to a lesser extent than in the case of ΔIDR (Fig. 4B, C and Supplementary Fig. 9C). Taken together, we provide evidence that TNS1 phase separation is mediated by its IDR, with major contributions from the IDR2 and IDR3 segments.

### TNS1 condensate properties are determined by its IDR

A number of recent studies have highlighted the importance of IDR sequence in determining condensate properties and functions^47–51^. To further understand the role of the TNS1 IDR in mediating its phase separation, we performed a compositional analysis of the IDR sequence, including its individual segments (IDR1, IDR2 and IDR3) and the respective deletion mutants (ΔIDR1, ΔIDR2 and ΔIDR3). This analysis revealed stronger disorder-promoting hydrophobicity patterns in the sequence corresponding to IDR3, which exhibits the highest disorder propensity (〈*λ*〉) values, as well as higher values of sequence hydrophobicity disorder when compared to IDR1 and IDR2 (Supplementary Fig. 9D). Despite its high disorder tendency, IDR3 contains the lowest fraction of charged residues (FCR) among the three IDR subregions and is uniquely characterised by a positive net charge per residue (NCPR), suggesting that the disorder properties of IDR3 derive from compositional factors beyond simple charge repulsion. Furthermore, deletion of IDR3 (ΔIDR3) leads to a marked shift in properties of the remaining IDR sequence, including an increase in aromatic clustering (Ω_aro_) and a fundamental change in sequence charge decoration from strongly negative to positive, highlighting the distinctive disorder and compositional properties of this region (Supplementary Fig. 9D). Together, our sequence composition analysis supports our experimental data and suggests that a unique combination of high disorder tendency with specific compositional features of the individual IDR segments play a critical role in modulating the overall phase separation behaviour of TNS1.

To further explore the importance of the TNS1 IDR sequence in determining TNS1 condensate properties, we generated GFP-TNS1 mutants where sequences corresponding to IDR2 and IDR3 were either swapped (IDR132) or randomly scrambled (IDR2scr, IDR3scr and IDR23scr) and expressed them in U2OS TNS1 KO cells with GFP-TNS1 WT used as a control. While we observed no difference in condensation propensity between TNS1 WT and IDR132 and only a slight increase in the case of IDR2scr, TNS1 IDR3scr and IDR23scr exhibited substantially higher tendency to form condensates when compared to TNS1 WT (Fig. 4D, E). In addition, TNS1 IDR3scr and IDR23scr condensates were smaller in size and more abundant (Fig. 4F, G). We next performed FRAP of the individual GFP-TNS1 condensate variants in U2OS TNS1 KO cells to further characterise their properties. In line with the observed differences in condensation propensity, these experiments revealed significantly slower GFP-TNS1 recovery rates in IDR3scr and IDR23scr condensates when compared to WT, while there was no significant difference in recovery of IDR132 and IDR2scr condensates (Fig. 4H, I and Supplementary Fig. 9E). Together, our data highlight the importance of TNS1 IDR sequence in determining the properties of TNS1 condensates.

### The TNS1 IDR regulates FA dynamics, cell migration and invasion

**Fig. 5:**
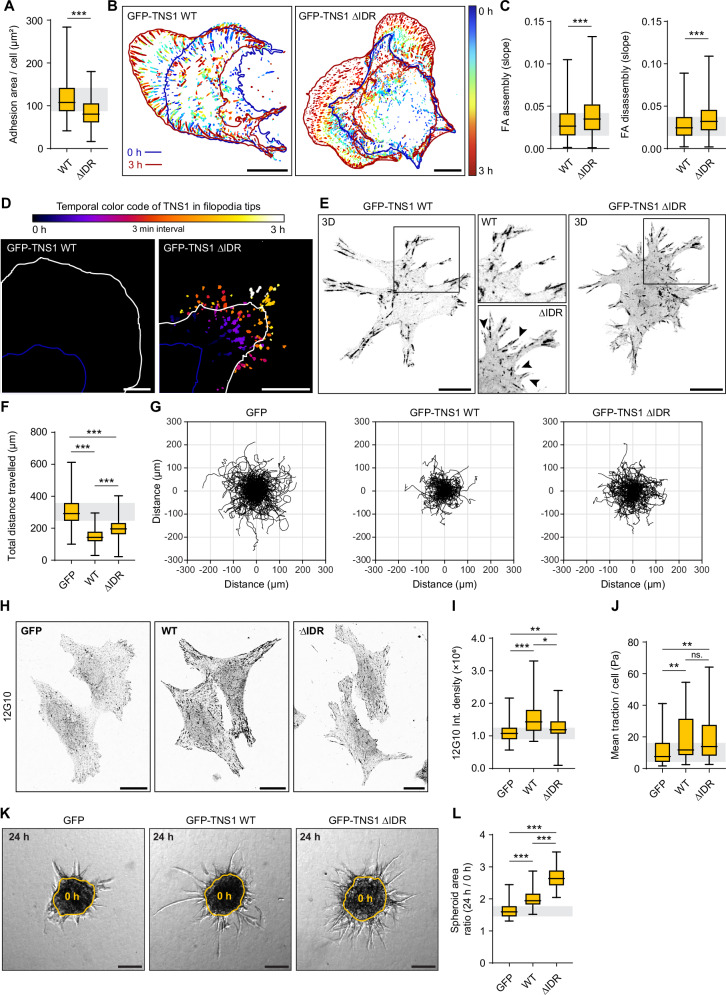
TNS1 IDR regulates FA dynamics, cell migration and invasion. **A** Quantification of the FA area from *n* = 165 (WT) and 154 (ΔIDR) cells per condition. **B** Representative images of FA dynamics in indicated cell lines. Time-lapse images acquired for 3 h at 3 min intervals, analysed by the Focal adhesion analysis server (FAAS)^103^. Colour-coded cell outlines from the initial (blue) and the last (red) timepoints are shown. Scale bars 20 μm. **C** Quantification of FA assembly (left) and disassembly (right) rates from the indicated cell lines performed using the FAAS^103^. Quantification was performed from *n* = 41 (WT) and 43 (ΔIDR) cells, analysing *n*(assembly) = 3334 (WT), 4594 (ΔIDR) and *n*(disassembly) = 3593 (WT), 5155 (ΔIDR) individual adhesions. Due to the technical limitations of FAAS, only cells with no condensates were analysed. **D** Colour-coded representative filopodia tip masks from time-lapse images of indicated cell lines migrating on FN-coated coverslips acquired as in Fig. 5B, C (images shown in Supplementary Fig. 10D). Outlines of cell protrusions at times *t* = 0 h (dark blue) and *t* = 3 h (white) are shown. Scale bars 10 μm. **E** Representative 3D projection of cells expressing indicated GFP-TNS1 variants embedded in 3D fibrillar rat tail collagen (multichannel images shown in Supplementary Fig. 10E). Arrowheads are indicating short TNS1-rich cell protrusions. Scale bars 10 μm. **F** Quantification of total distance travelled of *n* = 170 (GFP), 159 (GFP-TNS1 WT) and 161 (GFP-TNS1 ΔIDR) cells over the period of 10 h. **G** Cell migration trajectories of cells analysed in Fig. 5F. **H** Representative images of indicated cell lines stained with 12G10 antibody (active integrin β1). Scale bars 20 μm. **I** Quantification of the integrated density of the indicated cell lines stained with 12G10 antibody. Quantification was performed from *n* = 94 (GFP), 81 (GFP-TNS1 WT) and 88 (GFP-TNS1 ΔIDR) cells. **J** Quantification of mean traction force per cell from *n* = 45 (GFP), 51 (GFP-TNS1 WT) and 66 (GFP-TNS1 ΔIDR) cells. Representative images are shown in Supplementary Fig. 10L. **K** Representative images of 3D spheroid invasion assays using the indicated cell lines. Images at 24 h timepoint are shown with the spheroid outline at *t* = 0 h shown in yellow. Scale bars 100 μm. **L** Quantification of spheroid area ratio at 24 and 0 h from *n* = 62 (GFP), 65 (GFP-TNS1 WT) and 71 (GFP-TNS1 ΔIDR) spheroids. Statistical analysis was performed using two-tailed Mann–Whitney *U* test (**A**, **C**) and Kruskal–Wallis test with Dunn’s multiple comparisons test (**F**, **I**, **J**, **L**). *p* < 0.05 (*); *p* < 0.01 (**); *p* < 0.001 (***); ns. not significant. Source data, including exact *p*-values are provided as a Source data file.

We next assessed the functional roles of the TNS1 IDR in adhesion dynamics and cell behaviour. To this end, we generated TNS1 KO U2OS cell lines with inducible expression of either GFP alone, GFP-TNS1 WT or GFP-TNS1 ΔIDR, ensuring comparable expression levels between individual cell lines (Supplementary Fig. 10A). First, we assessed the characteristics of TNS1-containing FAs in cells expressing GFP-TNS1 WT or ΔIDR seeded on FN. While we observed no difference in average FA size between TNS1 WT and ΔIDR (Supplementary Fig. 10B), there was a significant reduction in FA area in cells expressing TNS1 ΔIDR (Fig. 5A), with a notable, although not statistically significant, increase in FA localisation towards the cell periphery (Supplementary Fig. 10C). In addition, the TNS1 ΔIDR-expressing cells exhibited higher FA assembly and disassembly rates when compared to TNS1 WT cells (Fig. 5B, C), suggesting a role for the IDR in the regulation of FA stability. In line with increased FA dynamics, TNS1 ΔIDR promoted short-lived filopodia tip adhesions and filopodia formation in cell protrusions, localising into filopodia tips in migrating cells (Fig. 5D and Supplementary Fig. 10D). Although compositionally different from FAs, filopodial tip adhesions have been previously shown to probe the cell environment and promote directed cell migration and invasion by maturation into nascent and focal adhesions^52–54^. Similarly, when embedded in 3D fibrillar collagen I, cells expressing TNS1 ΔIDR exhibited increased number of short protrusions emanating from the cell body (Fig. 5E and Supplementary Fig. 10E). Further characterisation of cells expressing GFP, GFP-TNS1 WT or GFP-TNS1 ΔIDR, revealed no significant differences in cell area (Supplementary Fig. 10F) or cell shape (Supplementary Fig. 10G), and functional experiments did not show any differences in cell proliferation (Supplementary Fig. 10H), or cell spreading monitored as a change in impedance on FN-coated xCELLigence plates (Supplementary Fig. 10I). Expression of both TNS1 WT and ΔIDR led to a substantial decrease in cell migration when compared to GFP-expressing cells, however, ΔIDR was able to partially revert the inhibitory effect of TNS1 WT on cell migration as evidenced by an increase in both migrated distance (Fig. 5F, G) and mean cell speed (Supplementary Fig. 10J), with an overall increase in migration directionality (Supplementary Fig. 10K). This correlated with a significantly increased amount of active integrin β1 (Fig. 5H, I), in line with previously described TNS1 roles in regulating integrin activity^25–28^. In addition, cells expressing either GFP-TNS1 WT or ΔIDR exerted higher traction forces on 10 kPa polyacrylamide gels than the GFP-expressing cells, but there was no significant difference in traction between the TNS1 variants (Fig. 5J and Supplementary Fig. 10L). Finally, expression of both GFP-TNS1 WT and ΔIDR led to a significant increase in cell invasion into 3D collagen I gels when compared to GFP-expressing cells, with ΔIDR invading substantially more than WT (Fig. 5K, L). Taken together, deletion of the IDR resulted in a gain-of-function phenotype characterised by enhanced FA dynamics, and increased cell protrusion, migration and invasion, reflecting either a direct role of the IDR or altered distribution of FA components upon IDR deletion.

### TNS1 condensation is regulated by stress-mediated phosphorylation

**Fig. 6:**
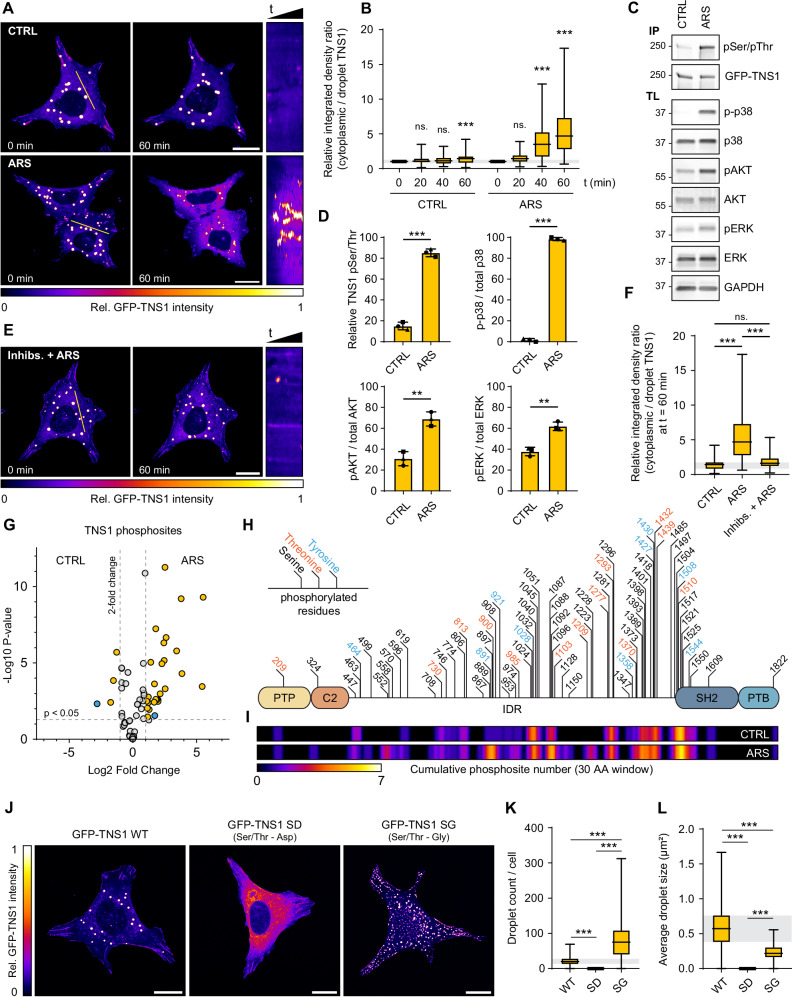
TNS1 condensation is regulated by stress-mediated phosphorylation. **A** Representative timelapse images (acquired for 60 min at 1 min intervals) of non-treated U2OS cells expressing GFP-TNS1 (CTRL) or cells treated with sodium arsenite (ARS). Images at *t* = 0 min and *t* = 60 min are shown, along with a kymograph of the whole time-lapse along the white line. Scale bars 20 μm. **B** Quantification of the ratio between the relative cytoplasmic and droplet TNS1 integrated densities at the indicated timepoints from *n* = 78 (CTRL) and 115 (ARS) cells. **C** Representative immunoblots from lysates of either CTRL or ARS-treated cells. GFP-TNS1 was immunoprecipitated to probe with anti-pSer/pThr antibody. Molecular weight markers are in kDa. **D** Quantification (*n* = 3) of relative TNS1 Ser/Thr phosphorylation and relative activity of p38, ERK and AKT kinases quantified as a ratio between phosphorylated and total protein. **E** Representative time-lapse images (acquired for 60 min at 1 min intervals) of U2OS cells expressing GFP-TNS1 pre-treated with 5 μM Doramapimod (p38), 5 μM Trametinib (MEK1/2) and 5 μM MK2206 (Akt) for 60 min before ARS treatment. Images at *t* = 0 min and *t* = 60 min are shown, along with a kymograph of the whole time-lapse along the white line. Scale bars 20 μm. **F** Quantification of the ratio between the relative cytoplasmic and droplet TNS1 integrated densities at *t* = 60 min from *n* = 78 (CTRL), 115 (ARS) and 82 (Inhibs.+ARS) cells. **G** Volcano plot of individual TNS1 phosphorylation sites as identified by mass spectrometry of GFP-TNS1 purified from CTRL or ARS-treated cells. Statistically enriched (>2-fold change at *p* < 0.05, unpaired t-test) Ser/Thr phosphosites (yellow) and Tyr phosphosites (blue) are indicated. **H** Schematic representation of localisation of the identified phosphosites within TNS1. **I** Cumulative phosphosite number over a 30-amino acid (AA) window within TNS1 purified from CTRL or ARS-treated cells. **J** Representative confocal images of U2OS TNS1 KO cell lines with inducible expression of indicated GFP-TNS1 variants. Scale bars 20  μm. **K** Quantification of TNS1 droplet count per cell from *n* = 339 (WT), 358 (SD) and 355 (SG) cells. **L** Quantification of average TNS1 droplet size per cell from *n* = 340 (WT), 358 (SD) and 353 (SG) cells. Statistical analysis was performed using the Friedman test with two-sided Dunn’s multiple comparisons test comparing the individual timepoints to *t* = 0 min (**B**), two-tailed unpaired t-test (**D**) and Kruskal–Wallis test with Dunn’s multiple comparisons test (**F**, **K**, **L**). *p* < 0.01 (**); *p* < 0.001 (***); ns. not significant. Source data, including exact *p*-values are provided as a Source data file.

Phosphorylation has been repeatedly shown to regulate protein condensation across organisms^16,55–60^. As our screen targeting canonical tyrosine phosphorylation-dependent integrin signalling pathways did not reveal any significant impact on TNS1 condensation, we next focused on Ser/Thr phosphorylation. Since a previous phosphoproteomic study demonstrated that TNS1 undergoes strong Ser/Thr phosphorylation in response to cell stress^61^, we next assessed the potential role of stress-mediated kinase activity in regulating TNS1 condensation using a well-established inducer of oxidative stress, sodium arsenite. Indeed, arsenite treatment of GFP-TNS1-expressing U2OS cells led to a significant translocation of TNS1 from the dense to the dilute phase (cytoplasm) (Fig. 6A, B and Supplementary Videos 7, 8), resulting in a substantial decrease in condensate size and area (Supplementary Fig. 11A, B). This correlated with a strong arsenite-induced increase in TNS1 Ser/Thr phosphorylation, as well as increased activation of major stress-activated kinases, including p38 and ERK mitogen-activated protein kinases (MAPKs) and AKT (Fig. 6C, D). To explore this further, we inhibited these kinases with a combination of specific inhibitors (Doramapimod (p38), Trametinib (MEK1/2, upstream of ERK) and MK2206 (AKT)) and observed completely impaired GFP-TNS1 translocation to the dilute phase upon arsenite treatment (Fig. 6E, F and Supplementary Video 9), confirming the role of these kinases in TNS1 phosphorylation. As controls, we used U2OS cell lines with inducible expression of either GFP-TNS2, a TNS1 paralog, or GFP fused to Pop-tag, a short protein domain driving cytoplasmic protein condensation^62^. In the case of GFP-TNS2, arsenite stimulation led to a small but significant translocation of the protein from the condensates to the dilute phase; however, its effect was strikingly lower when compared to GFP-TNS1 (Supplementary Fig. 11C–E). In contrast, arsenite treatment of cells expressing GFP-Pop-tag led to a significant increase in condensate formation (Supplementary Fig. 11C, D, F). To further support our findings, we assessed the effect of combined p38, ERK and AKT inhibition on endogenous TNS1 condensation. Indeed, treating the U2OS mGL-TNS1 cells seeded on FN- or PLL- coated coverslips with the combination of inhibitors as described above resulted in increased formation of TNS1 circular spots when compared to the respective DMSO-treated controls (Supplementary Fig. 11G). Finally, to extend our observations to a disease-relevant model, we examined GFP-TNS1 condensation in HUVECs treated with inflammatory stimuli, as TNS1 recruitment to adhesions has previously been demonstrated in endothelial responses during endotoxemia^33^. In line with our findings from U2OS cells, treatment of HUVECs expressing GFP-TNS1 with tumour necrosis factor (TNF)-α, a known activator of p38 MAPK^63^, resulted in a significant disassembly of GFP-TNS1 condensates (Supplementary Fig. 11H, I). Taken together, our results demonstrate that TNS1 condensation is negatively regulated by protein phosphorylation in response to different extracellular stimuli and that this mechanism is protein-specific, rather than a general characteristic of phase-separated proteins.

### TNS1 phosphorylation affects IDR charge patterning and prevents condensate assembly

To identify the phosphorylation sites involved in regulating TNS1 phase separation, we next performed a phosphoproteomic analysis of GFP-TNS1 purified from either control (CTRL, untreated) or arsenite-treated (ARS) cells (Fig. 6G and Supplementary Fig. 12A). In line with our previous results (Fig. 6C, D), we observed a substantial increase in Ser/Thr phosphorylation in GFP-TNS1 purified from arsenite-treated cells with 26 Ser/Thr sites being significantly enriched (>2-fold) when compared to control (Fig. 6G). Interestingly, most of the total of 50 Ser, 13 Thr and 9 Tyr phosphorylated residues identified in our phosphoproteomic data were localised within the TNS1 IDR (Fig. 6H), highlighting the importance of this region in TNS1 condensation in response to its phosphorylation state. Moreover, comparing the cumulative phosphosite number over a 30 AA window between control and arsenite-treated conditions revealed several phosphorylation 'hotspots' within the IDR (Fig. 6I), leading to a dramatic shift in net charge decoration in these regions (Supplementary Fig. 12B), which has previously been shown to affect phase separation in the context of nuclear condensates^64–66^.

We next assessed the condensation propensity of the different TNS1 phosphorylation states. Since our results suggest that TNS1 condensation is regulated by p38-, ERK- and AKT-mediated phosphorylation (Fig. 6E, F), we analysed the probability of the individual phosphosites to be phosphorylated by these kinases using the Kinase Library tool^67^ (Supplementary Fig. 12C). Based on the prediction, we selected 36 Ser/Thr sites among the constitutively phosphorylated and significantly enriched sites in arsenite-treated cells (Supplementary Fig. 12C, bottom heatmap) and substituted them for either phosphomimicking (aspartate, TNS1 SD) or unphosphorylatable (glycine, TNS1 SG) residues. Notably, the substitutions did not have any adverse effect on protein expression, and we were able to generate TNS1 KO U2OS cell lines with inducible expression of individual GFP-TNS1 variants at comparable levels (Supplementary Fig. 12D, E). Interestingly, expression of the GFP-TNS1 SD mutant completely impaired the formation of TNS1 condensates. In contrast, the expression of GFP-TNS1 SG led to a substantial increase in TNS1 condensate number and total area per cell, with a decrease in average condensate size when compared to WT (Fig. 6J–L and Supplementary Fig. 12F). Taken together, our results support the role of phosphorylation in the negative regulation of TNS1 condensation in a p38/ERK/AKT-dependent manner.

### TNS1 phosphorylation controls FA dynamics and cell migration

**Fig. 7:**
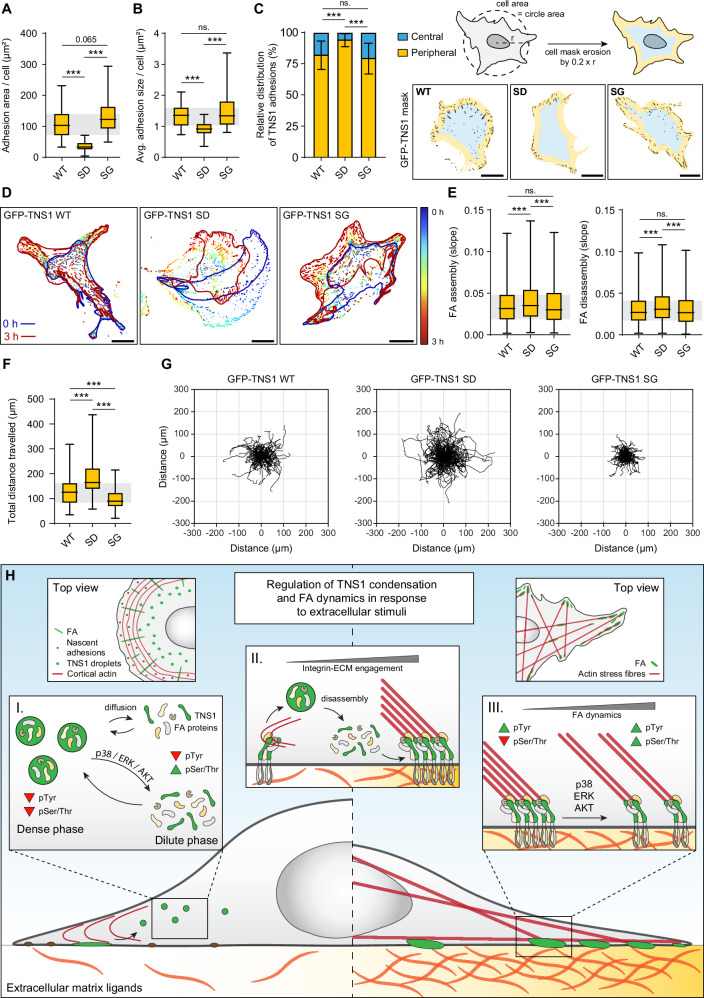
TNS1 phosphorylation controls FA dynamics. **A** Quantification of adhesion area per cell from *n* = 34 (WT), 31 (SD) and 42 (SG) cells. **B** Quantification of average adhesion size per cell from *n* = 34 (WT), 32 (SD) and 42 (SG) cells. **C** Quantification of the relative distribution of peripheral and central FAs from the indicated cell lines, error bars represent standard deviation. Analysis performed from *n* = 34 (WT), 30 (SD) and 42 (SG) cells by eroding the cell mask by the factor of 0.2 × radius (*r*) of a circle with area corresponding to the area of the cell. Representative images corresponding to central and peripheral areas with GFP-TNS1 adhesion masks are shown. Scale bars 20 μm. **D** Representative images of FA dynamics in indicated cell lines. Time-lapse images acquired for 3 h at 3 min intervals were analysed by the Focal adhesion analysis server (FAAS)^103^. Colour-coded cell outlines from the initial (blue) and the last (red) timepoints are shown. Scale bars 20 μm. **E** Quantification of FA assembly (left) and disassembly (right) rates performed using the FAAS^103^. Quantification was performed from *n* = 34 (WT), 32 (SD) and 42 (SG) cells, analysing *n*(assembly) = 1912 (WT), 1958 (SD), 1900 (SG) and *n*(disassembly) = 2234 (WT), 1439 (SD) and 1926 (SG) individual adhesions. **F** Quantification of total distance travelled of *n* = 136 (WT), 153 (SD) and 134 (SG) cells over the period of 10 h. **G** Cell migration trajectories of cells analysed in Fig. 7F. **H** Limited availability of integrin ECM ligands promotes formation of TNS1 condensates that selectively buffer the local concentration of unphosphorylated FA components. TNS1 condensates dynamically exchange material with the surrounding cytoplasm by passive diffusion or disassemble upon TNS1 phosphorylation by stress-activated kinases (I.). Increased integrin-ECM engagement leads to TNS1 condensate disassembly in proximity of newly forming adhesions, thereby increasing the adjacent cytoplasmic pool of adhesion proteins that can be recruited to FAs (II.). Activation of stress-induced kinases in cells on ECM-rich substrates leads to increased FA dynamics promoting directed cell migration (III.), likely mediated by increased phosphorylation of TNS1 in FAs and higher availability of FA components upon TNS1 condensate disassembly. Statistical analysis was performed using one-way (**A**) or 2-way (**C**) ANOVA with Holm–Šídák’s multiple comparisons test (**A**, **C**), Kruskal–Wallis test with Dunn’s multiple comparisons test (**B**, **E**, **F**). *p* < 0.001 (***); ns. not significant. Source data, including exact *p*-values are provided as a Source data file.

Finally, we characterised the effect of TNS1 phosphorylation on the regulation of FA dynamics and cell behaviour, using the TNS1 KO U2OS cells with inducible expression of either GFP-TNS1 WT, SD or SG as described above. Analysis of FA properties revealed a significant decrease in total FA area in cells expressing TNS1 SD, and a notable, although not statistically significant, increase in TNS1 SG-expressing cells (Fig. 7A). In addition, the average FA size was reduced in cells expressing TNS1 SD (Fig. 7B) and the FAs were significantly more localised at the cell periphery (Fig. 7C), without substantial changes in cell area (Supplementary Fig. 13A) or cell shape (Supplementary Fig. 13B), suggesting a potential role for TNS1 phosphorylation in regulating FA maturation and dynamics. Indeed, analysis of FA dynamics confirmed a significant increase in both FA assembly and disassembly rates in cells expressing TNS1 SD (Fig. 7D, E), similarly to the cells expressing TNS1 ΔIDR (Fig. 5B, C). Also, consistent with our observations following TNS1 ΔIDR expression, TNS1 SD cells exhibited an increase in cell migration distance (Fig. 7F, G) and speed (Supplementary Fig. 13C), with higher cell directionality (Supplementary Fig. 13D) when compared to WT and SG. In contrast, cells expressing TNS1 SG showed a marked reduction in cell migration distance (Fig. 7F, G) and speed (Supplementary Fig. 13C). Together, our data suggest a role of TNS1 phosphorylation in regulating FA dynamics and maturation, with direct implications for adhesion dynamics and cell migration.

## Discussion

In this study, we characterised the role of TNS1 condensation in regulating FA dynamics. Through comprehensive validation, we demonstrated that TNS1 undergoes concentration-dependent phase separation in cells, including at endogenous protein levels. Using proximity biotinylation proteomics, we identified the principal components of TNS1 condensates and validated their role in maintaining unphosphorylated FA protein pools in FA proximity. Furthermore, we identified the TNS1 IDR as a region driving TNS1 condensation and determining condensate properties and established its role in regulating TNS1-mediated biological functions. Finally, we demonstrated that TNS1 phosphorylation by stress-activated kinases negatively regulates TNS1 condensate formation, as well as FA dynamics and cell migration (Fig. 7H). Our data indicate that stress-signals can act as physiologically relevant triggers for the release of adhesion proteins from cytoplasmic reservoirs to rapidly support new adhesion formation in their close proximity.

The complexity of FA organisation and its dynamic regulation has prompted views that FAs should be characterised as phase-separated molecular assemblies^5,6^. Indeed, a number of individual FA components, including GIT, PIX^18^, FAK^7^, CAS^7,68^, LIMD1^16^, PXN^19^, TLN^5^, KANK1^17^, TNS1^15^ and TNS3^20^, have been observed to form condensates in vitro and/or in cells, either alone or in combination with other FA components. Intriguingly, we demonstrated that TNS1 condensates are selective in nature, recruiting a subset of FA components in their unphosphorylated state, rather than all components non-specifically. Considering the previous findings, our results suggest a potential hierarchical organisation within FA-derived condensates; however, systematic studies evaluating protein recruitment to individual condensates are required to address this aspect comprehensively.

We demonstrated that a local increase in endogenous TNS1 concentration upon FA disassembly or lack of integrin-ECM engagement results in the formation of TNS1 condensates. This is in line with the proposed functions of phase separation in buffering local biomolecule concentrations into compartmentalised systems^9,38,55,69^. The sequestering role of TNS1 condensates is also supported by our results, suggesting that TNS1 phase separation can acutely increase the amounts of recruited proteins, possibly by protecting them from degradation. Together, our observations address a longstanding question of how expression levels and local availability of FA components are maintained in the proximity of adhesion sites.

As part of our BioID screen validation, we described a close spatial association between TNS1 condensates and P-bodies, highly reminiscent of previously observed contacts between P-bodies and stress granules^70–72^. The highly dynamic stress granule-P-body contacts have been shown to enable exchange of messenger ribonucleoproteins to facilitate mRNA fate decision towards decay or translation reinitiation after release from polysomes^70,71^. While the absence of detectable polyA-mRNA in TNS1 condensates suggests different functional implications for the TNS1 condensate-P-body contacts, it is feasible to hypothesise a potential component exchange between the two compartments. For instance, phase separation of LIM domain-containing 1 (LIMD1) protein, with previously described P-body functions^73^, has recently been shown to regulate FA dynamics, highlighting the possibility of crosstalk between FA-derived condensates and P-bodies. Moreover, recent evidence that individual P-body components can modulate stress granule composition and their association with P-bodies^72^ raises intriguing questions about the mechanisms and regulation of P-body-TNS1 condensate interactions to be addressed in future studies.

We provide evidence that TNS1 condensation is driven by its IDR. In addition, the biophysical properties of TNS1 condensates are determined by the IDR sequence. This is particularly interesting in the context of differences between the individual tensin paralogs. While the structurally ordered domains at the N- and C- terminus of TNS1-3 are highly conserved, their IDRs share very little sequence homology^21^, implying their importance in regulating some of the distinct functions of the tensin family members^21,22^. Notably, in contrast to our findings that TNS1 condensates are regulated by stress-kinase signalling but not by substrate stiffness, a recent study by Li et al. reported mechanosensitive behaviour of TNS3 condensates dependent on TLN1 binding^20^. As the TLN-binding site within TNS1 is not fully homologous to that of TNS3, these differences can likely be attributed to distinct TLN1 binding. Similarly, potential differences in TLN1 binding are likely to underlie the distinct protein recruitment into TNS1 and TNS3 condensates. While we demonstrate that TLN1, VCL and KANK2 show very weak or limited recruitment to TNS1 condensates, they do localise to TNS3 condensates in a TLN1-dependent manner^20^. We also show that, in the case of TNS1, deletion of the IDR resulted in enhanced promigratory and protrusive phenotypes stimulated by the conserved domains. It remains a focus of future studies to determine whether the same regulation holds true also for the other tensin family members.

We further demonstrate that predominantly IDR-targeted TNS1 phosphorylation by stress-activated kinases p38, ERK and AKT regulates TNS1 condensation and FA dynamics. In contrast to Tyr phosphorylation largely associated with active integrin signalling, Ser/Thr phosphorylation of FA proteins is far less understood. Although tightly balanced levels of CDK1-mediated phosphorylation are essential for maintaining FA stability^74–76^, hyperphosphorylation has been widely linked to FA disassembly, e.g. by disrupting FAK/p130Cas interactions within FA complexes^77,78^ or by targeting phosphorylated kindlin for degradation before mitotic entry^79^. Interestingly, although TNS1 harbours 312 Ser/Thr residues, phosphomimetic substitution of only 36 of them had a striking effect on TNS1 condensation and its FA-mediated biological functions. Moreover, since p38/ERK MAPKs and CDK1 kinases exhibit a strong preference for Ser/Thr-Proline motifs, it is plausible to hypothesise that they share at least some of the phosphorylation sites within TNS1, providing an additional level of regulatory control in key biologically relevant processes, such as the essential steps of adhesion disassembly and assembly during cell division^80,81^.

It is important to note that the stress-responsive kinases are activated by diverse physiological stimuli that, among other processes, drive EMT and cancer progression. For instance, TGFβ acts as a potent activator of both p38 and ERK MAPKs, as well as AKT^82,83^. In line with the role of TGFβ in promoting EMT and cell migration^84^, expression of the TNS1 mutant mimicking phosphorylation by these kinases resulted in increased FA dynamics and migratory cell behaviour consistent with a more mesenchymal phenotype. Therefore, phosphorylation-controlled TNS1 condensates acting as FA protein reservoirs may represent a previously unknown level of control of cell morphology changes linked to EMT. Beyond mesenchymal cells and cancer, TNS1 is implicated in increasing vascular barrier permeability in response to inflammatory cytokines, such as TNF-α, in endotoxemia^33^. We show here that acute TNF-α treatment results in rapid loss of TNS1 condensates in endothelial cells. TNS1 is also enriched in neutrophils and monocytes (Human Protein Atlas). While its role in these cells remains largely unexplored, our data may explain early observations of TNF-mediated p38 activation acting as a stop signal that promotes firm neutrophil adhesion and inhibits chemotaxis towards interleukin-8^85^. Furthermore, kinase-dependent regulation of TNS1 could contribute to the crosstalk between integrin and MAPK signalling that has been demonstrated in multiple physiological contexts. Acute integrin β1-dependent p38 activation was observed in human osteosarcoma cells^86^ and in cardiomyocytes subjected to mechanical stress^87,88^. In addition, interleukin-8 production in natural killer cells has been shown to be dependent on integrin β1-mediated p38 activation^89^. Our results therefore support the existence of a regulatory feedback loop in which acute integrin-mediated activation of stress-associated kinases leads to phosphorylation of FA components, thereby increasing FA dynamics and promoting cell migration.

## Methods

### Plasmids and cloning

The plasmids encoding pEGFP-TNS1 WT, pEGFP-p130CAS and mKate2-PXN have been described previously^29,40,52^. For simplicity, the abbreviation GFP instead of EGFP is used throughout the study, except in the plasmid names. The TNS1 isoform used in this study corresponds to the canonical UniProt entry Q9HBL0-3, missing the amino acids 1–125. In case of phosphorylation sites, we always use the numbering corresponding to the full Q9HBL0-3 isoform as annotated on phosphosite.org.

Individual TNS1 mutants (ΔPTP, ΔC2, ΔIDR, ΔSH2, ΔPTB, ΔIDR1, ΔIDR2 and ΔIDR3) were generated by a whole plasmid synthesis approach using homemade *Pfu-X7* high-fidelity polymerase^90^, pEGFP-TNS1 WT as a template and the respective primers (listed in Supplementary Data 1). PCR was carried out for 18 cycles, and the reactions were purified using KAPA Pure Beads (Roche), digested with *DpnI* (Thermo Fisher Scientific) and transformed into *E. coli* DH5α competent bacteria. pEGFP-TNS1 SD and SG were generated by NEBuilder® HiFi DNA Assembly (New England Biolabs) of the commercially synthesised coding sequences (TNS1 SD_1/2, TNS1 SG_1/2, sequences listed in Supplementary Data 1, GenScript) and the backbone of pEGFP-TNS1 WT cleaved by *XhoI*/*SalI*. pEGFP-TNS1 IDR132, IDR2scr, IDR3scr and IDR23scr were generated by NEBuilder® HiFi DNA Assembly (New England Biolabs) of the commercially synthesised coding sequences (IDR132_1/2, IDR2scr_1/2, IDR3scr_1/2, IDR23scr_1/2 and TNS1 C-term, sequences listed in Supplementary Data 1, GenScript) and the backbone of pEGFP-TNS1 WT cleaved by *EcoRI/SalI*. The sequences corresponding to IDR2 and IDR3 were scrambled using a custom MATLAB script with random permutation of characters (available upon request).

pSB EGFP-TNS1 WT/ΔIDR/SD/SG constructs were generated by cloning from pEGFP-TNS1 WT/ΔIDR/SD/SG into the SleepingBeauty (SB) transposon vector (pSB MCS) via *AgeI*/*XhoI* and *AgeI*/*SalI* sites, respectively.

TNS1 BioID construct was generated by NEBuilder® HiFi DNA Assembly (New England Biolabs). TNS1 WT cDNA was PCR-amplified from pEGFP-TNS1 WT using *Pfu-X7* polymerase^90^ with the respective primers (TNS1 Hifi F/R) and assembled into pPB-BirA* vector^91^ cleaved with *BspEI*.

All the constructs were verified by analytical digestion and sequencing.

### Cell lines, cell culture and transfection

Human osteosarcoma cell line, U2OS (ATCC), has been used throughout the study unless indicated. The cells were cultured in Dulbecco’s Modified Eagle’s Medium supplemented with 10% fetal bovine serum (FBS), 100 U/ml penicillin, 0.1 mg/ml streptomycin and 2 mM L-glutamine. The cells were kept in a humidified incubator with 5% CO_2_ at 37 °C. Cell transfection was performed using Lipofectamine 3000 (Invitrogen), and siRNA was transfected using Lipofectamine RNAiMAX (Invitrogen) following the manufacturer’s instructions.

U2OS cell lines with inducible expression of individual GFP-TNS1 variants were generated by co-transfecting the cells with pSB EGFP-TNS1 (WT/ΔIDR/SD/SG) plasmid together with a vector encoding hyperactive SB transposase (pSB100X)^92^ in a 5:1 ratio. Ten days after transfection, expression of GFP-TNS1 was induced by adding 0.25 μg/ml doxycycline for 48 h, and the cells were sorted using SONY SH800S Cell Sorter for GFP fluorescence. After expansion, the cells were sorted one more time to achieve a cell population with uniform expression. Before each experiment, unless indicated, GFP-TNS1 expression was induced for at least 48 h by 0.25 μg/ml doxycycline.

U2OS cell lines stably expressing TNS1-BioID were generated by co-transfecting the cells with pPB TagBFP-T2A-TNS1-Myc-BioID plasmid together with a vector encoding a hyperactive transposase (pCMV-hyPBase)^93^ in a 4:1 ratio. Ten days after transfection, the cells were sorted based on TagBFP fluorescent signal into two populations with either low or high expression. This correlated with TNS1-Myc-BioID expression as validated by IF and western blot.

Commercially available primary pooled donor HUVECs (PromoCell) were amplified for 3 passages to generate a stock of vials, ensuring reproducible experimental conditions in ECGM basal medium supplemented with Supplemental mix (PromoCell) and 1% penicillin-streptomycin (Sigma). For each experiment, a fresh vial of HUVECs was thawed on a 10 cm plate. After 2 days, cells were nucleofected with pEGFP-TNS1 WT plasmid using the Neon Transfection System (Thermo Fisher Scientific) and the Neon Transfection System 10 μl Kit (Thermo Fisher Scientific), used according to the manufacturer’s instructions (30 ms pulse, 1.35 V). After nucleofection, the cells were seeded directly on fibronectin-coated µ-Slide 8 Well (Ibidi) and left in complete media for 2 days.

### Generation of TNS1 knock-out cell line

The CRISPR/Cas9 guide sequence (TGAGAGCGGAACATACCGCT) targeting Exon 17 of TNS1-201 transcript (Transcript ID-ENST00000171887.8) was designed using CRISPOR^94^ and cloned into pX330 U6-Chimeric hSpCas9-Venus plasmid^95^ via *BbsI* (New England Biolabs) sites. U2OS cells were transfected with the corresponding plasmid and, 48 h after transfection, Venus-positive cells were sorted using SONY SH800S Cell Sorter. Single cell clones were raised from the sorted population, and knock-out efficiency was determined using an immunoblot with anti-TNS1 antibody. For further validation, genomic DNA from 5 TNS1 knock-out (KO) clones and 5 clones with unaffected TNS1 expression levels was isolated and used as a template to PCR-amplify a 566 bp-long fragment surrounding the cleavage site (F: GGGACACTCTGGGACTTGTG; R: GTCCTACCCCATGGAGCCTA) that was sequenced. Validated clones were pooled into TNS1 KO and TNS1 ctrl populations.

### TNS1 endogenous tagging

U2OS cells with endogenously tagged TNS1 were generated using homology-directed repair-mediated (HDR) CRISPR/Cas9 targeting Exon 6 of TNS1-201 transcript (Transcript ID - ENST00000171887.8). The corresponding guide sequence (CATGAGTGTGAGCCGGACCA) was cloned into pXPR_001 lentiCRISPR v1 plasmid (Addgene #49535), cleaved with *BsmBI* (NEB). Final construct (pXPR_001-sgTNS1) was verified by sequencing. Template for HDR was designed based on a double-cut dsDNA donor approach^96^. The sequence of a bright monomeric fluorescent protein, mGreenLantern^39^, followed by a short linker in frame with the TNS1 open reading frame, was surrounded by ca. 850 base pairs-long homology arms. The whole cassette was flanked with sequences recognised by the TNS1 Cas9 guide to generate a double-cut dsDNA donor for the HDR. The sequence was synthesised into the pUC57mini cloning vector (pUC57-TNS1 HDR, sequence listed in Supplementary Data 1, GenScript). U2OS cells were co-transfected with pXPR_001-sgTNS1 and pUC57-TNS1 HDR plasmids in 1:4 ratio using Lipofectamine 3000 (Invitrogen). Five days after transfection, the cells were sorted using SONY SH800S Cell Sorter for green fluorescence and re-sorted again after expansion to obtain a cell population with the highest fluorescent signal. The specificity of TNS1 endogenous tagging was verified using IF with a TNS1-specific antibody and siRNA knockdown of TNS1 (Supplementary Fig. 2B–D).

### Immunofluorescence

Cells were seeded on ECM-coated glass coverslips either for 3-4 h or overnight as indicated and fixed for 10 min in pre-warmed 3% PFA (Thermo Fisher Scientific) in PBS at 37 °C. ECM components and coatings used in this study were FN (10 μg/ml, bovine, Sigma), VTN (10 μg/ml, recombinant human, Gibco), LMN (10 μg/ml, recombinant human LMN-521, BioLamina), COL I (10 μg/ml, in-house, rat tail), COL IV (10 μg/ml, mouse, Cultrex) and PLL (12.5 μg/ml, Sigma). Fixed cells were washed with PBS three times for 5 min at RT. When needed, cells were permeabilised using a permeabilization buffer containing 10% horse serum (Gibco) and 0.3% Triton X-100 Surfact-Amps (Thermo Fisher Scientific) in PBS for 10 min at RT, followed by three 5 min washes with PBS. Primary and secondary antibodies were incubated in 4% horse serum (Gibco) in PBS for 1 h at RT, with three washes with PBS between and after the respective incubations. For immunofluorescence of cells in 3D fibrillar collagen matrices, cells were embedded in a mixture of in-house rat-tail collagen I (final concentration ca. 1.5 mg/ml) and collagen I conjugated with Atto647N (conjugated in-house as described before^97^) in 1× DMEM (Thermo Fisher Scientific) supplemented with 1% FBS, 2.7 mg/ml sodium bicarbonate (Sigma), and 20 mM HEPES (Sigma). Collagen was polymerised for 15 min at RT and overlayed with culture medium. After 3 h, cells were fixed and permeabilized as described above. HUVEC cells were fixed in 4% PFA (Thermo Fisher Scientific) and stained with DAPI (Life Technologies) and Phalloidin Atto647N (Sigma). To assess the role of TNF-α on TNS1 condensation, transfected HUVECs were grown to monolayers and treated with control medium or medium supplemented with 5 ng/ml TNF-α (RnD Systems). After 2.5 h of treatment, cells were fixed and stained as described above. The antibodies used for IF were as follows: anti-TNS1 (Sigma, HPA036089); anti-FAK (BD Transduction Laboratories, 610088); anti-p130CAS (Cell Signalling Technologies (CST), 13846S); anti-ZYX (Abcam, ab109316); anti-KANK2 (Sigma, HPA015643); anti-TLN1 (Novus Biologicals, NBP2-50320); anti-TLN2 (BioRad, MCA4771GA); anti-VCL (Sigma, V9131); anti-PXN (GeneTex, GTX125891); anti-DCP1B (CST, 13233S); anti-DDX6 (Bethyl Laboratories, A300-460A); anti-EDC3 (Abcam, ab168851); anti-pY (BD Transduction Laboratories, 610000); anti-pCAS Y410 (CST, 4011S); anti-pFAK Y397 (CST, 8556S); anti-pPXN Y118 (CST, 2541S); anti-pZYX S142/143 (CST, 4863S); anti-active ITGB1 clone 12G10 (isolated from hybridoma cells, AB_928074); anti-Myc-Tag (CST, 2276S); anti-TNS3 (Rb33; gift from K. Clark, University of Leicester, Leicester, England, UK^22^). Streptavidin conjugated with AlexaFluor680 was used to detect protein biotinylation. DAPI (Life Technologies) was used to detect cell nuclei, and either Phalloidin Atto647N (Sigma) or Spy-FastAct (Spirochrome) probes were used to stain Actin. WGA AlexaFluor647 was used to stain cell membranes. Alexa Fluor–conjugated secondary antibodies (Alexa Fluor 488-, 555-, 568- and 647-conjugated anti-mouse and anti-rabbit; Thermo Fisher Scientific) were used.

### Image acquisition

Fixed samples were imaged using Marianas spinning disk confocal microscope with a Yokogawa CSU-W1 scanning unit and equipped with an Orca Flash4 sCMOS camera (Hamamatsu Photonics). The objectives used were 40×/1.1 NA W LD C- Apochromat (Zeiss) and 63×/1.4 NA O PLAN-Apochromat (Zeiss). Images were acquired using SlideBook 6 software (Intelligent Imaging Innovations). For imaging of live cells, the samples were maintained at 37 °C and 5% CO_2_ in a stage top humidified incubator (Okolab). Cells embedded in 3D collagen were imaged using a Leica Stellaris 8 Falcon FLIM microscope with HC PL APO 86×/1.20 NA W motCORR objective. Images were acquired using LAS X software. 3D invasion assays were imaged using Leica Thunder equipped with a 5×/0.12 NA N PLAN objective and Leica K8 camera. Images were acquired using LAS X software with Navigator. Cell migration assays were imaged on Nikon Eclipse Ti2-E equipped with a 10×/0.3 NA Nikon CFI PLAN-Fluor objective and Hamamatsu sCMOS Orca Flash 4.0 camera. Images were acquired using NIS-Elements AR 5.11.01 software. The samples were maintained at 37 °C and 5% CO_2_ in a stage top humidified incubator (Okolab).

Super-resolved 3D structural illumination microscopy (SR-SIM) was performed using Deltavision OMX V4 (Applied Precision, GE Healthcare) equipped with a 60×/1.42 NA SIM PLAN Apo N (Olympus) objective and a 3× PCO edge Front illuminated sCMOS camera. Immersion oil with refraction index *n*_d_ = 1.518 and Abbe number *ν*_d_ = 41 (Olympus) was used. Images were acquired using OMX Acquisition v3.70 software, and SIM reconstruction was performed in softWoRx Deconvolution v7.0.0. Chromatic shift of the reconstructed SIM images was corrected using Chromagnon v0.91^98,99^ based on 0.2 μm TetraSpeck microsphere (Invitrogen) data.

### Western blotting

Protein extracts were resolved using SDS-PAGE (4-20% gradient, BioRad) under denaturing conditions, and proteins were transferred to low fluorescence Immobilon®-FL PVDF membrane (Milipore). Membranes were blocked using AdvanBlock-Fluor blocking solution diluted with PBS in a 1:1 ratio. Antibodies were diluted in the blocking solution at indicated concentrations, primary antibodies were incubated overnight at 4 °C, and secondary antibodies were incubated for 1 h at RT. Primary antibodies used were as follows: anti-GFP (1:5000, Abcam, ab290); GAPDH (1:4000, HyTest, 5G4 MAb 6C5); anti-TNS1 (1:1000, Sigma, SAB4200283); anti-ZYX (1:1000, Abcam, ab109316); anti-PXN (1:1000, GeneTex, GTX125891); anti-TLN1 (1:1000, Novus Biologicals, NBP2-50320); anti-VCL (1:1000, Sigma, V9131); anti-Myc-Tag (1:1000, CST, 2276S); anti-ITGB1 (1:1000, BD Transduction Laboratories, 610468); anti-ITGA5 (1:1000, Invitrogen, PA5-82027); anti-pSer/Thr (1:1000, BD Transduction Laboratories, 612549); anti-p38 (1:1000, CST, 9212S); anti-p-p38 (1:1000, CST, 9216S); anti-AKT (1:1000, CST, 2920S); anti-p-AKT (1:1000, CST, 9275S); anti-ERK (1:1000, CST, 4696S); anti-p-ERK (1:1000, CST, 4370S). Streptavidin AlexaFluor680 was used to detect protein biotinylation. Relevant AzureSpectra Fluorescent Secondary antibodies with 490, 650 and 800 fluorophores were used at 1:3000 dilution throughout the study. Membranes were washed using TBST in between and after antibody incubations and scanned using Sapphire FL Biomolecular Imager (Azure Biosystems).

### Compound screen

U2OS mGL-TNS1 cells were seeded at FN-coated glass-bottom 24-well plates, left to adhere overnight and treated with either DMSO or indicated compounds as follows: blebbistatin (25 μM, 60 min, StemCell Technologies, 72402); calpeptin (50 μM, 30 min, Selleckchem, S7396); H1152 (5 μM, 120 min, MedChemExpress, HY-15720); lapatinib (0.5 μM, 120 min, MedChemExpress, HY-50898); LY294002 (10 μM, 60 min, Selleckchem, S1105); MG132 (10 μM, 4 h, MedChemExpress, HY-13259); nocodazole (10 μM, 4 h, MedChemExpress, HY-13520); Ro3306 (20 μM, 60 min, MedChemExpress, HY-12529); saracatinib (1 μM, 60 min, Selleckchem, S1006); vanadate (1 mM, 30 min, Sigma, 5086050004); Y27632 (10 μM, 60 min, MedChemExpress, HY-10071). The cells were fixed and stained using WGA and DAPI to detect the cell membrane and nuclei, respectively.

### Fluorescence recovery after photobleaching (FRAP)

U2OS cells expressing the indicated GFP-TNS1 variants were imaged using a 3i spinning disk microscope with 1 s interval between timeframes. A z-stack of 9 images (0.27 μm step) was acquired for 9 s before and for 100–110 s after photobleaching one GFP-TNS1 droplet per cell. Bleaching was performed with 1 ms laser burst across all z-stacks. The acquired time-lapse images were analysed in Fiji (ImageJ). First, the maximum intensity projection of the whole field of view was corrected for bleaching (histogram matching), and the individual photobleached droplets were then analysed separately in a cropped ROI. The individual timepoints were aligned using the droplet as a landmark to account for droplet movement during analysis. The intensity spectra of photobleached droplets were combined, normalised to min-max values and a batch FRAP fit was applied to all spectra (Fiji plugins by Jay Unruh, Stowers Institute for Medical Research in Kansas City, MO).

### Correlative light-electron microscopy (CLEM)

U2OS pSB GFP-TNS1 cells induced with doxycycline for 48 h were seeded on fibronectin-coated gridded glass bottom dish (MatTek) and left to adhere overnight. Cells were fixed with warm 4% PFA (Thermo Fisher Scientific) in 0.2 M HEPES (pH 7.4) for 10 min at 37 °C and washed twice with 0.2 M HEPES. Cells were stained with DAPI, and a confocal z-stack was acquired using a 3i CSU-W1 spinning disk microscope with 63× objective as described above. Subsequently, the cells were fixed with 2% glutaraldehyde (Sigma) in 0.2 M HEPES for 2 h at RT and washed twice with 0.2 M HEPES. The samples were than post-fixed with 1% osmium tetroxide and 1.5% potassium ferrocyanide solution, dehydrated in Ethanol and embedded in Epon resin. Thin sections (70 nm) were cut on an ultramicrotome, collected on single-slot electron microscopy copper grids, and stained with 1% uranyl acetate and 0.3% lead citrate solutions. The samples were imaged at 80 kV on a Jeol JEM-1400 Plus transmission electron microscope. 19 electron micrographs of the cell of interest at ×4000 magnification were stitched together using the TrakEM2 plugin in Fiji (ImageJ). The resulting montage was overlaid with maximum intensity projection of 3 bottom slices (z-step 0.27 μm) of the confocal stack using the ec-CLEM plugin in Icy (https://icy.bioimageanalysis.org/). The images were correlated with non-rigid transformation using GFP-TNS1 condensates as landmarks. High magnification images of GFP-TNS1 condensates were imaged at 20000×.

### Immunoprecipitation

U2OS cells expressing GFP-tagged proteins were seeded on one 10 cm plate per condition (15 cm for MS). Cells were washed twice with ice-cold PBS, scraped into residual PBS, transferred into 1.5 ml tube, and centrifuged at 400 × *g* for 2 min at 4 °C. Cell pellet was resuspended in 500 μl of lysis buffer containing 75 mM Tris (pH 7.4), 200 mM NaCl, 2 mM MgCl_2_, 1.5% Triton X-100 and 0.75% NP-40, supplemented with Complete protease inhibitors mix (Roche), PhosStop phosphatase inhibitors mix (Roche), and GENIUS Nuclease (Santa Cruz) in recommended concentrations. The lysates were incubated on ice for 20 min with occasional gentle vortexing to facilitate cell lysis and centrifuged at 13,000 × *g* for 10 min at 4 °C. Part of the cleared lysates was heated with 8× reducing sample buffer to generate total lysate samples. The remaining lysate was incubated for 2 h at 4 °C with GFP-Trap Magnetic Agarose (Chromotek) previously washed twice with a wash buffer containing 50 mM Tris pH 7.4, 150 mM NaCl and 1% NP-40. After the incubation, the beads were washed once with lysis buffer, twice with wash buffer, eluted into 2× reducing sample buffer and analysed by SDS-PAGE and immunoblot. Samples for MS analysis were washed an additional three times with TBS (50 mM, Tris pH 7.4, 150 mM NaCl) and stored dry at −80 °C until further processed.

### BioID sample preparation

The sample preparation for BioID was carried out as described previously^100^. The U2OS cell lines stably expressing Myc-BioID only, and with low and high expression of TNS1-Myc-BioID, were seeded on two 10 cm plates and supplemented with biotin to a final concentration of 50 μM for at least 18 h. After labelling, the cells were washed twice with PBS and harvested into lysis buffer containing 50 mM Tris (pH 7.4), 500 mM NaCl, 0.2% SDS, 1 mM DTT and protease inhibitors (Roche). Lysates were transferred to a tube containing 20% TritonX-100 (final concentration 2%), passed four times through a 21 G needle, and 50 mM Tris pH 7.4 (360 μl per plate) was added to the tubes. Subsequently, the lysates were passed additional four times through a 27 G needle and centrifuged at 16,000 × *g* for 10 min at 4 °C. Biotinylated proteins were captured from the cleared lysates by overnight rotation with magnetic Streptavidin beads (ReSyn Biosciences). After incubation, the beads were washed (5 min at RT per wash) twice with 2% SDS, once with a buffer containing 0.1% sodium deoxycholate, 1% Triton X-100, 500 mM NaCl, 1 mM EDTA and 50 mM HEPES (pH 7.5) and once with a buffer containing 0.5% NP-40, 0.5% sodium deoxycholate, 1 mM EDTA and 10 mM Tris. Cl (pH 7.4). Proteins were eluted in 2× reducing sample buffer saturated with biotin, heated at 70 °C for 10 min and subjected to SDS-PAGE, followed by reduction, alkylation and in-gel digestion. Samples from four independent biological replicates were analysed by MS.

### Mass spectrometry and data analysis

The LC-ESI-MS/MS analyses were performed on a nanoflow HPLC system (Easy-nLC1200, Thermo Fisher Scientific) coupled to the Exploris 480 mass spectrometer (Thermo Fisher Scientific) equipped with a nano-electrospray ionisation source and FAIMS interface. Digested peptide samples were dissolved in 0.1% formic acid and injected for analysis. Wash runs were submitted between samples to reduce peptide carry-over. Peptides were first loaded on a trapping column and subsequently separated inline on a 15 cm C18 column (75 μm × 15 cm, ReproSil-Pur 3  μm 120 Å C18-AQ, Dr. Maisch HPLC GmbH). The mobile phase consisted of water with 0.1% formic acid (solvent A) and acetonitrile/water (80:20 (v/v)) with 0.1% formic acid (solvent B).

For BioID samples, a 60 min non-linear gradient was used to elute peptides (50 min from 5% to 21% solvent B, followed by 22 min from 21 % to 36 min solvent B and in 5 min from 36% to 100% of solvent B, followed by 5 min wash stage with solvent B). MS data were acquired automatically using Thermo Xcalibur 4.1 software (Thermo Fisher Scientific). A data dependent acquisition method consisted of repeated cycles of one MS1 scan covering a range of m/z 300–1750 plus a series of HCD fragment ion scans (MS2 scans) for the most intense peptide ions from the MS1 scan. Raw data files were analysed using MaxQuant software (version 2.0.3.0) and the MS/MS spectra were searched against a human database (Uniprot-SwissProt, version 230427) using Andromeda search engine. Search parameters were set to trypsin digestion with two missed cleavages, carbamidomethyl (C) as a fixed modification, and oxidation (M), acetyl (protein N-term), biotinylation (K) and biotinylation (protein N-term) as variable modifications. The identified peptides were filtered based on FDR 0.01. Unique peptide counts identified for each protein group were subjected to statistical analysis using SAINTexpress (Significance Analysis of INTeractome)^42^ in SPC mode with default settings (n-iter 4000, lowMode off, minFold on, normalise on) and the results were visualised using ProHits-viz^101^. Raw MS data have been deposited to the ProteomeXchange Consortium via the PRIDE partner repository^102^ (PXD069837) and to Zenodo (10.5281/zenodo.17426146). Processed data are available as a supplementary file (Supplementary Data 2).

For GFP-TNS1 IP samples, a 120 min gradient was used (62 min from 5 to 21% solvent B, followed by 48 min from 21 to 36% solvent B and in 5 min from 36 to 100% of solvent B, followed by 5 min wash stage with solvent B). Samples were analysed by a data-independent acquisition (DIA) LC-MS/MS method. MS data were acquired automatically using Thermo Xcalibur 4.6 software (Thermo Fisher Scientific). In the DIA method, a duty cycle contained a full scan (m/z 395–m/z 1005) and 30 DIA MS/MS scans covering the m/z range of 400–1000 with variable-width isolation windows. Raw data from 4 independent replicates were analysed in Spectronaut (Biognosys, version 19.2.). The DirectDIA approach was used to identify proteins against the human protein database (Uniprot-SwissProt, version 2409), and label-free quantifications were performed using MaxLFQ. Fixed modifications were set to carbamidomethyl (C), variable modifications were set to oxidation (M), acetyl (protein N-term) and phospho (STY). The identified peptides were filtered based on FDR 0.01, and phosphopeptides were filtered based on localisation probability set to 0.7. Normalisation on GFP-TNS1 quantity was performed, and the differential abundance of the identified phosphosites was tested using an unpaired t-test. Raw MS data have been deposited to the ProteomeXchange Consortium via the PRIDE partner repository^102^ (PXD069806) and to Zenodo (10.5281/zenodo.17408117). Processed data are available as a Supplementary file (Supplementary Data 3).

### Preparation of hydrogels

Thirty-five millimetre glass-bottom dishes (Cellvis) were treated with 500 µl bind-silane solution (7.14% bind-silane (Sigma) and 7.14% acetic acid in absolute EtOH) for 1 h at room temperature and washed twice with 2 ml absolute EtOH before being allowed to air-dry. To get the desired hydrogel stiffness (0.5 kPa, 2 kPa, 10 kPa, or 20 kPa), 40% (w/v) acrylamide (Sigma) and 2% *N,N’*-methylenebisacrylamide solution (Sigma) were mixed in PBS with defined ratios (Supplementary Table 1) and briefly vortexed. For TFM gels, 1.7 µl 0.2 μm fluorescent beads (FluoSpheres, Invitrogen) were sonicated (7 min, 30 s on, 30 s off) and added to 500 µl hydrogel mix. Polymerisation of the hydrogels was initiated through the addition of 10% ammonium persulfate (final concentration 0.1% (v/v), Bio-Rad) and *N,N,N’N’*-tetramethylethylenediamine (final concentration 0.2% (v/v), Sigma) and the mixture was rapidly vortexed. 11.8 µl was immediately added to the glass-bottom dishes, and a clean 13 mm glass coverslip was placed on top. After 1 h incubation at RT to allow polymerisation, PBS was added, and coverslips were carefully removed. The hydrogel surface was activated by a 30 min incubation with 500 µl 0.2 mg/ml Sulfo-SANPAH (Sigma) and 2 mg/ml *N*-(3-dimethylaminopropyl)-*N*′-ethylcarbodiimide hydrochloride (Sigma) in 50 mM HEPES with gentle agitation, followed by irradiation with UV light for 10 min. Hydrogels were washed four times with PBS and coated with 10 μg/ml FN (Sigma) for 1 h at 37 °C.

### Real-time quantitative PCR

RNA extracts were prepared using the NucleoSpin RNA kit (Macherey Nagel) following the manufacturer’s instructions. cDNA synthesis was performed using the High-Capacity cDNA Reverse Transcription Kit (Applied Biosystems). Real-time quantitative PCR was performed on QuantStudio 3 (ThermoFisher Scientific) using pre-designed TaqMan gene expression assays as indicated (GAPDH Hs02786624_g1; ZYX Hs00170299_m1; PXN Hs01104424_m1). Data analysis was performed using QuantStudio Design & Analysis Software 1.5.2.

### Functional assays

#### Proliferation

The indicated cell lines were seeded in a technical triplicate at 8000 cells/96-well plate and allowed to grow for 48 h. Proliferation was assessed using the AlamarBlue HS reagent (Thermo Fisher Scientific) according to the manufacturer’s instructions. Three independent biological replicates were performed.

#### Cell spreading

Cell spreading was assessed using xCELLigence RTCA eSight (Agilent). 15,000 cells per well were seeded in technical triplicate into a fibronectin-coated E-Plate View 96 (Agilent). Changes in impedance were monitored for 1 h after seeding the cells at 10 min intervals and normalised to the first time point. Three independent biological replicates were performed.

#### Focal adhesion dynamics

Cells were seeded on fibronectin-coated µ-Slide 8 Well (Ibidi) and allowed to adhere for 3 h. Individual cells were imaged for 3 h at 3 min intervals, acquiring a z-stack of 5 stacks with 0.5 μm spacing. During image acquisition, the cells were maintained in FluoroBrite DMEM (Gibco) supplemented with 10% FBS and 2 mM L-glutamine. Adhesion dynamics were analysed using the Focal Adhesion Analysis Server (FAAS)^103^ with the following settings: a minimum adhesion size of 20 pixels, a minimum adhesion phase length of 3 images, and a detection threshold for segmentation of 1.5 for GFP-TNS1 SG, 2.0 for WT and ΔIDR and 4.0 for SD. All the remaining settings were kept at their default values.

#### Spheroid invasion

MicroTissue 3D Petri Dishes (Sigma) were used to prepare 2% agarose moulds for spheroid formation. In each mould, 100,000 cells were seeded and incubated for 48 h. The spheroids were then embedded in a mixture of in-house rat-tail collagen (final concentration ca. 1.5 mg/ml) in 1× DMEM (Thermo Fisher Scientific) supplemented with 1% FBS, 2.7 mg/ml sodium bicarbonate (Sigma), and 20 mM HEPES (Sigma). After polymerisation, the collagen gels were overlaid with cell culture medium, and the spheroids were imaged at the indicated time points using a Leica Thunder microscope equipped with a 5× objective (as described above).

#### Dynamic release

Glass-bottom μ-slide 8-wells (Ibidi) were coated with a combination of PLL(20)-g[3.5]-PEG(2)/PEG(3.4)-biotin(50%) (25 μg/ml, SuSoS) and PLL (25 μg/ml, Sigma) in 10 mM HEPES pH 7.4 for 1 h at RT. Cells were seeded for 3 h and, after acquiring the initial time point, the medium was supplemented with streptavidin-conjugated FN (750 ng/cm^2^, conjugated in-house as described before^43^).

#### Cell migration

Cells were seeded on FN-coated µ-Slide 8 Well (Ibidi) and allowed to adhere for 3 h. Time-lapse imaging was performed over 10 h with 10 min intervals in three independent replicates using Nikon Eclipse Ti2-E equipped with a 10× objective as described above. Individual cells were tracked using either TrackMate^104^ with pretrained Cellpose 2.0 segmentation^105^ or manual tracking, and the resulting tracks were visualised using a custom MATLAB script (available upon request).

#### Traction force microscopy (TFM)

Cells were seeded on 10 kPa acrylamide gels with 0.2 μm fluorescent beads (FluoSpheres, Invitrogen) prepared as described above and left to adhere for 3 h. One hour before the beginning of the experiment, the cells were incubated in cell culture medium supplemented with 20 mM HEPES, 1 μg/ml Hoechst 33342, and 1 μg/ml WGA. Images were acquired using a spinning disk confocal microscope equipped with 40× water immersion objective as described above. Prior to imaging the relaxed gels, the cells were removed by adding 20% SDS into the media to a final concentration of 0.1%. Alignment of 'before' and 'after' images across all channels was performed using the Fast4DReg^106^ plugin in Fiji (ImageJ). The displacement field and traction forces were calculated in MATLAB (v. R2023a) using the TFM package^107^ with subpixel correlation via image interpolation and the FTTC (Fourier Transform Traction Cytometry) method with regularisation parameter set to 0.0001, respectively. Traction forces underneath the individual cell masks were extracted using a custom R script (available upon request) and used for quantification.

### Statistics, reproducibility and data visualisation

All experiments were performed in at least three independent biological replicates, unless otherwise indicated. For imaging data, micrographs shown are representative of at least three independent replicates. For experiments with less than three independent replicates, sufficient sample sizes (cells, measurements, etc.) were used to ensure the scientific relevance of the results. Statistical analyses and data visualisation were performed using GraphPad Prism 8. Outliers were identified by the ROUT method (*Q* = 0.2%) and excluded from subsequent analyses. Datasets were assessed for normal distribution to determine the appropriate statistical test. Statistical tests used are specified in the corresponding figure legends, and exact *p*-values are included in the Source data files. Throughout the whole study, boxplots represent median (middle line) and Q1–Q3 range (box) with whiskers extending to minimum and maximum values.

### Reporting summary

Further information on research design is available in the Nature Portfolio Reporting Summary linked to this article.

## Supplementary information

Supplementary Information

Description of Additional Supplementary Files

Supplementary Data 1

Supplementary Data 2

Supplementary Data 3

Supplementary Video 1

Supplementary Video 2

Supplementary Video 3

Supplementary Video 4

Supplementary Video 5

Supplementary Video 6

Supplementary Video 7

Supplementary Video 8

Supplementary Video 9

Reporting Summary

Transparent Peer Review File

## Source data

Source Data

## Data Availability

Data supporting the findings of this study are available within the paper and its source data, Supplementary information files. Statistical source data and uncropped and unprocessed blots are provided for all figures. Proteomic datasets have been deposited to ProteomeXchange Consortium via the PRIDE partner repository (PXD069837 [http://proteomecentral.proteomexchange.org/cgi/GetDataset?ID=PXD069837], PXD069806) and to Zenodo (10.5281/zenodo.17426146, 10.5281/zenodo.17408117). Source data are provided with this paper (Source data). Source data are provided with this paper.
