# Supplementary material for: Adhesion-derived condensates control component availability to regulate adhesion dynamics
Source: Nat Commun. 2026 Jun 5;17:7222. doi: 10.1038/s41467-026-74001-3 (PMC13396368; doi:10.1038/s41467-026-74001-3)
Supplement: Supplementary file 1 — Supplementary Information [file 41467_2026_74001_MOESM1_ESM.pdf]

Supplementary information

Adhesion-derived condensates control component availability to regulate adhesion dynamics

Michal Dibus[1,2,*], Megan R. Chastney[1,2,#], Giray Enkavi[3,#], Gautier Follain[1,2,4,5], Omkar Joshi[1,2], Ilpo Vattulainen[3], Johanna Ivaska[1,2,6-8,*]

[1]Turku Bioscience Centre, University of Turku and Åbo Akademi University, FI-20520 Turku, Finland
[2]InFLAMES Research Flagship, University of Turku, FI-20014 Turku, Finland
[3]Department of Physics, University of Helsinki, P.O. Box 64, FI-00014 Helsinki, Finland
[4]Faculty of Science and Engineering, Cell Biology, Åbo Akademi University, FI-20520 Turku, Finland
[5]Turku Collegium for Science, Medicine and Technology, TCSMT, University of Turku, FI-20520, Turku, Finland
[6]Department of Life Technologies, University of Turku, FI-20520 Turku, Finland
[7]Western Finnish Cancer Center (FICAN West), University of Turku, FI-20520 Turku, Finland
[8]Foundation for the Finnish Cancer Institute, Tukholmankatu 8, FI-00014 Helsinki, Finland
#These authors contributed equally to this work
*Correspondence: michal.dibus@utu.fi (M.D.), joivaska@utu.fi (J.I.)

Supplementary Figures 1–13

Supplementary Table 1.

Supplementary Figures

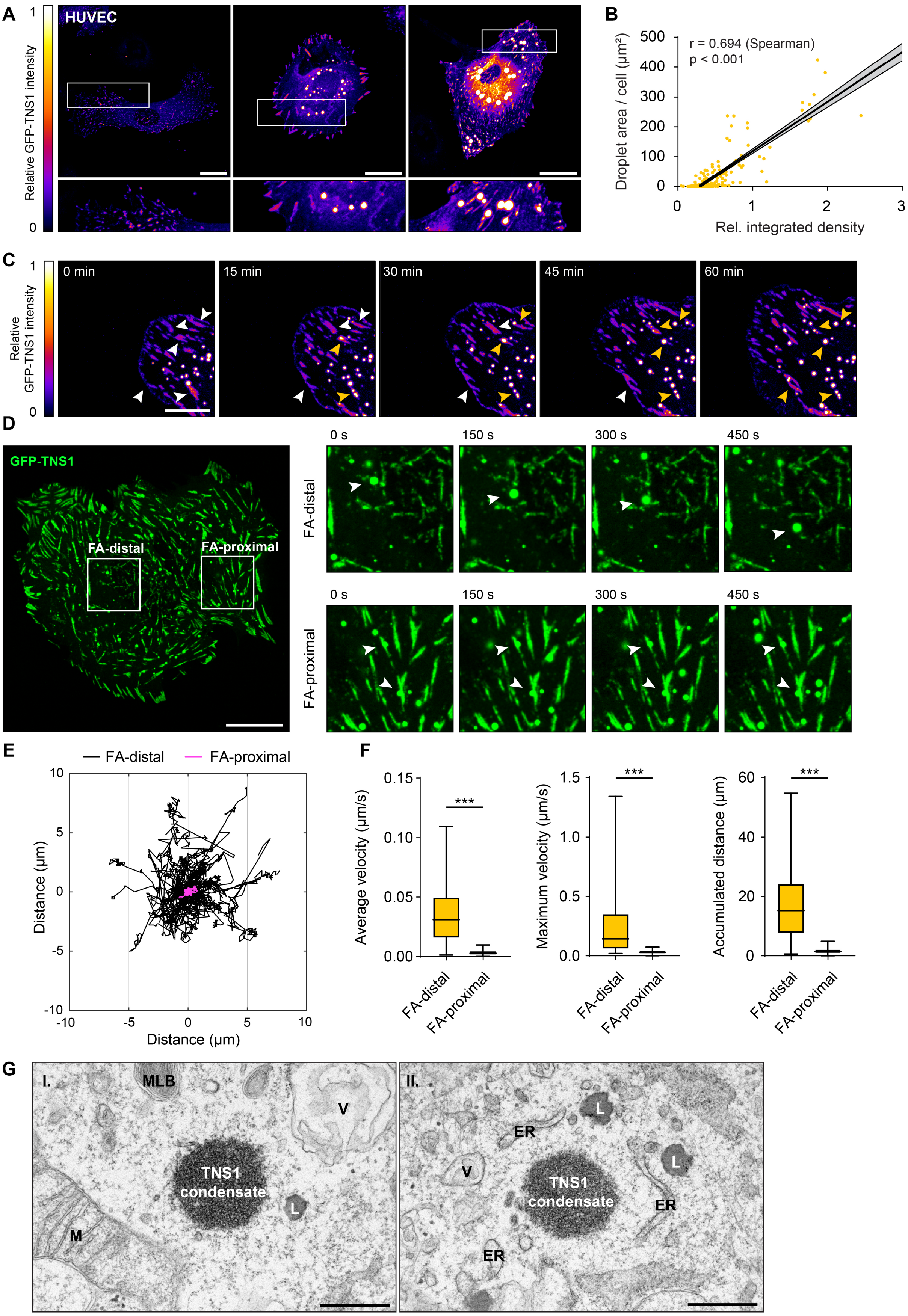

Supplementary Fig. 1.

- **A** Representative confocal images of HUVEC cells with varying expression of GFP-TNS1. Scale bar 20 μm.
- **B** Correlation between relative expression of GFP-TNS1 in HUVEC cells and the total condensate area per cell, represented as linear regression with 95% confidence intervals. Quantified from n = 236 cells across three biological replicates.
- **C** Representative confocal time-lapse images of TNS1 condensate formation upon focal adhesion (FA) disassembly (Supplementary Video 3). White arrows indicate FAs, yellow arrows indicate the appearance of TNS1 condensates from the respective FAs. Images acquired for 60 min at 3 min intervals. Scale bar 10 μm.
- **D** Time-lapse images from Supplementary Video 4. Closeups illustrate behaviour of GFP-TNS1 condensates either proximal or distal to focal adhesions as indicated by arrowheads. Images acquired for 495 s with 5 s intervals. Scale bar 20 μm.
- **E** Plot of trajectories tracking individual FA-distal (n = 74) or FA-proximal (n = 64) GFP-TNS1 condensates in U2OS cells.
- **F** Quantification of average velocity, maximum velocity and accumulated distance of FA-distal (n = 74) and FA-proximal (n = 64) GFP-TNS1 condensates tracked in Supplementary Fig. 1E. Statistical analysis was performed with two-tailed Mann-Whitney U test. $p < 0.001$ (***).
- **G** Annotation of subcellular structures in electron micrographs shown in Fig. 1F. MLB – multilamellar body; V – vesicle; M – mitochondria; ER – endoplasmic reticulum; L – lipid droplet. Scale bar 500 nm.

Source data including exact p values are provided as a Source Data file.

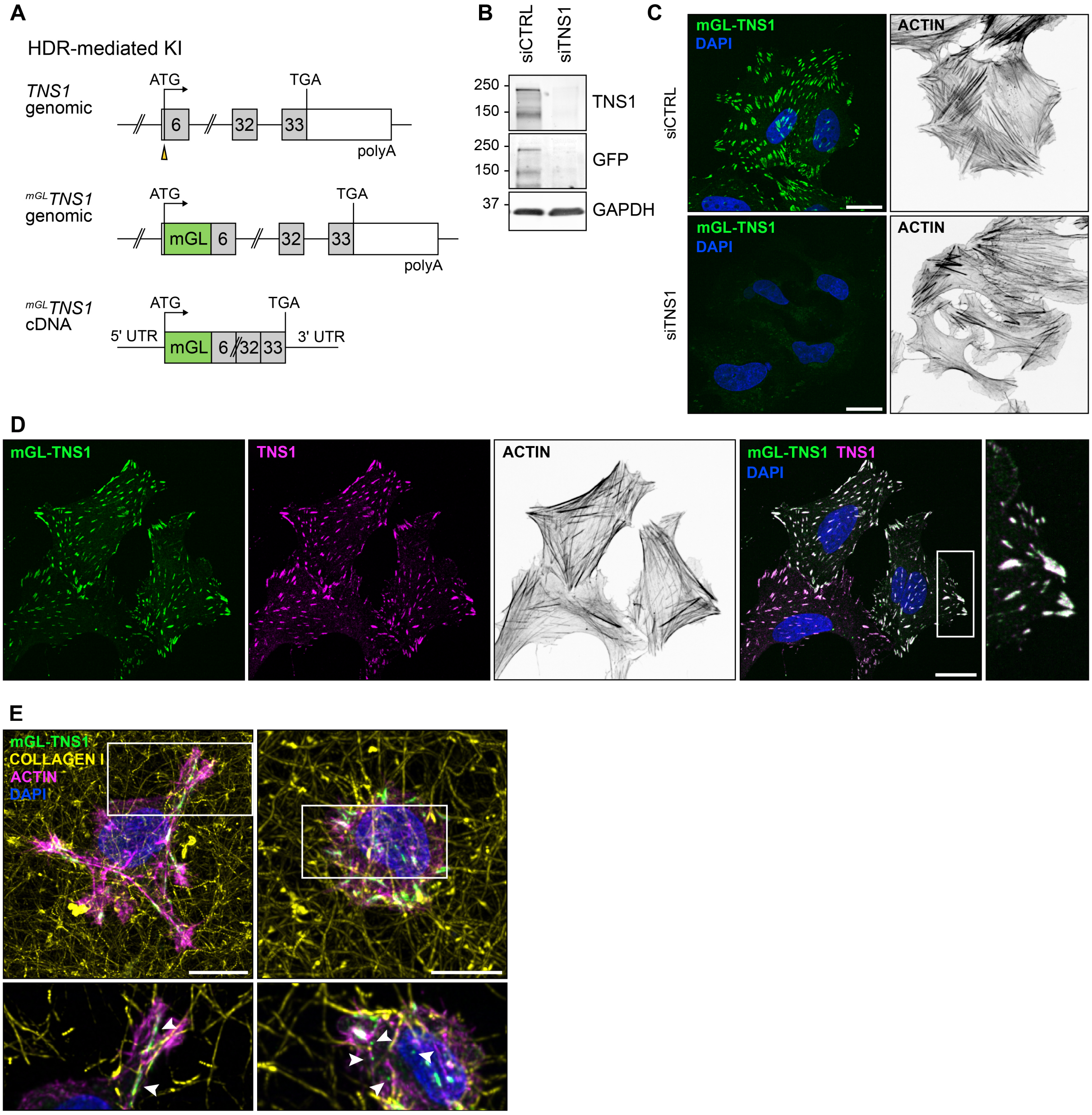

Supplementary Fig. 2.

- **A** Schematic representation of CRISPR/Cas9-mediated knock-in of mGreenLantern (mGL) cDNA into Exon6 of the TNS1 gene. The knock-in resulted in production of N-terminally tagged TNS1.
- **B** Expression of endogenous TNS1 in U2OS mGL-TNS1 cells was silenced using siRNA to confirm specificity of the knock-in. Immunoblots with anti-TNS1, anti-GFP (recognizes mGL) and anti-GAPDH antibodies are shown. Molecular weight markers are in kDa.
- **C** Representative confocal images of U2OS mGL-TNS1 cells transfected with control or TNS1-specific siRNAs. Scale bars 20 µm.
- **D** Representative confocal images of U2OS mGL-TNS1 cells stained with anti-TNS1 antibody. Actin was stained using Phalloidin. Scale bar 20 µm.
- **E** Representative confocal images of U2OS mGL-TNS1 cells embedded in 3D fibrillar rat tail collagen I conjugated with Atto647N. Cells were stained for F-Actin and DAPI. Maximum 3D projections are shown, closeups represent a maximum projection of 4 stacks at 0.72 µm. Arrows in the closeups indicate either FAs in contact with the underlying collagen fibres (left) or mGL-TNS1 condensates (right). Scale bars 10 µm.

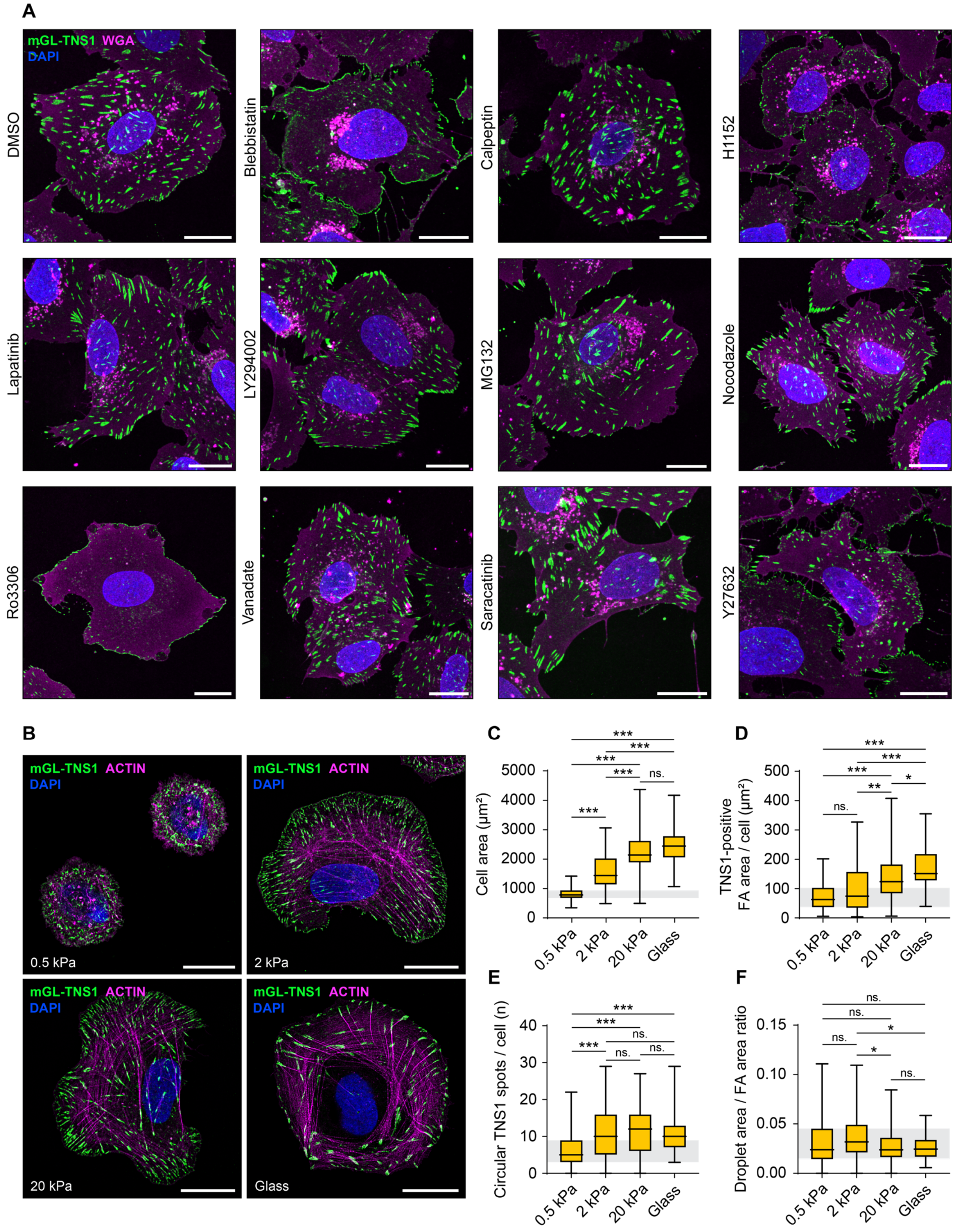

Supplementary Fig. 3.

- **A** Representative confocal images of U2OS mGL-TNS1 cells treated with the indicated compounds. Cells were stained with WGA and DAPI. Scale bars 20 μm.
- **B** Representative confocal images of U2OS mGL-TNS1 cells seeded on substrates with indicated stiffness. Scale bars 20 μm.
- **C** Quantification of cell area from n = 99 (0.5 kPa), 99 (2 kPa), 74 (20 kPa) and 67 (glass) cells as in Supplementary Fig. 3B.

D Quantification of mGL-TNS1-positive FA area from n = 103 (0.5 kPa), 99 (2 kPa), 75 (20 kPa) and 67 (glass) cells (based on particle circularity 0-0.9) as in Supplementary Fig. 3B.

E Quantification of number of mGL-TNS1-positive circular spots from n = 103 (0.5 kPa), 99 (2 kPa), 75 (20 kPa) and 65 (glass) cells (based on particle circularity 0.9-1) as in Supplementary Fig. 3B.

F Quantification of the ratio between the area of mGL-TNS1-positive circular spots and FA area from n = 100 (0.5 kPa), 95 (2 kPa), 75 (20 kPa) and 67 (glass) cells as in Supplementary Fig. 3B.

Statistical analysis was performed using Kruskal-Wallis test with Dunn's multiple comparisons test (**C**-**F**). $p < 0.05$ (*); $p < 0.01$ (**); $p < 0.001$ (***); ns. – not significant. Source data including exact p values are provided as a Source Data file.

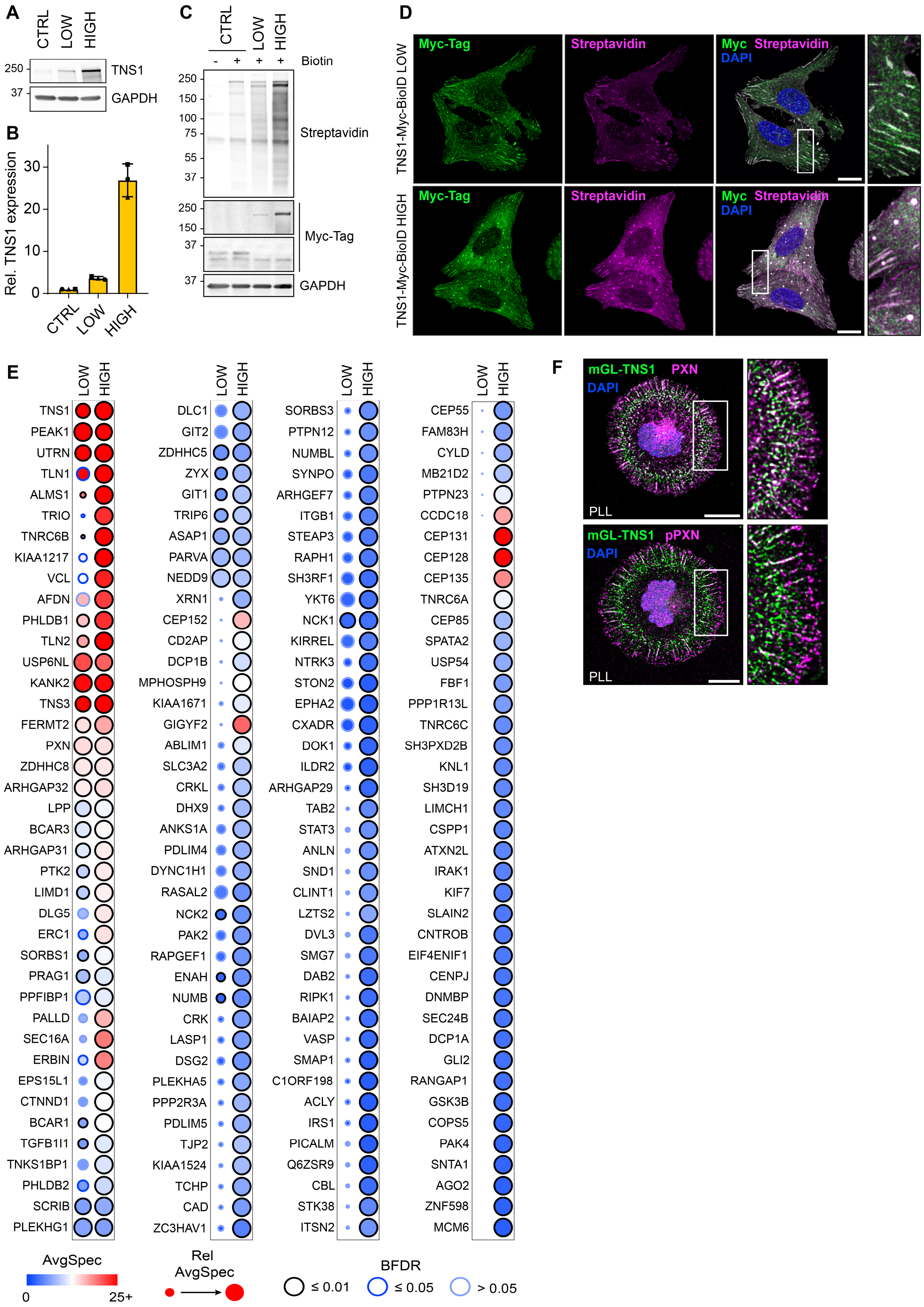
A
CTRL
LOW
HIGH
250
TNS1
37
GAPDH
B
Rel. TNS1 expression
30
20
10
0
CTRL
LOW
HIGH
C
CTRL
LOW
HIGH
-
+
+
+
Biotin
250
150
100
75
55
37
Streptavidin
250
150
37
Myc-Tag
37
GAPDH
D
TNS1-Myc-BioID LOW
TNS1-Myc-BioID HIGH
Myc-Tag
Streptavidin
Myc Streptavidin
DAPI
E
LOW
HIGH
TNS1
PEAK1
UTRN
TLN1
ALMS1
TRIO
TNRC6B
KIAA1217
VCL
AFDN
PHLDB1
TLN2
USP6NL
KANK2
TNS3
FERMT2
PXN
ZDHHC8
ARHGAP32
LPP
BCAR3
ARHGAP31
PTK2
LIMD1
DLG5
ERC1
SORBS1
PRAG1
PPFIBP1
PALLD
SEC16A
ERBIN
EPS15L1
CTNND1
BCAR1
TGFB1I1
TNKS1BP1
PHLDB2
SCRIB
PLEKHG1
DLC1
GIT2
ZDHHC5
ZYX
GIT1
TRIP6
ASAP1
PARVA
NEDD9
XRN1
CEP152
CD2AP
DCP1B
MPHOSPH9
KIAA1671
GIGYF2
ABLIM1
SLC3A2
CRKL
DHX9
ANKS1A
PDLIM4
DYNC1H1
RASAL2
NCK2
PAK2
RAPGEF1
ENAH
NUMB
CRK
LASP1
DSG2
PLEKHA5
PPP2R3A
PDLIM5
TJP2
KIAA1524
TCHP
CAD
ZC3HAV1
SORBS3
PTPN12
NUMBL
SYNPO
ARHGEF7
ITGB1
STEAP3
RAPH1
SH3RF1
YKT6
NCK1
KIRREL
NTRK3
STON2
EPHA2
CXADR
DOK1
ILDR2
ARHGAP29
TAB2
STAT3
ANLN
SND1
CLINT1
LZTS2
DVL3
SMG7
DAB2
RIPK1
BAIAP2
VASP
SMAP1
C1ORF198
ACLY
IRS1
PICALM
Q6ZSR9
CBL
STK38
ITSN2
CEP55
FAM83H
CYLD
MB21D2
PTPN23
CCDC18
CEP131
CEP128
CEP135
TNRC6A
CEP85
SPATA2
USP54
FBF1
PPP1R13L
TNRC6C
SH3PXD2B
KNL1
SH3D19
LIMCH1
CSPP1
ATXN2L
IRAK1
KIF7
SLAIN2
CNTROB
EIF4ENIF1
CENPJ
DNMBP
SEC24B
DCP1A
GLI2
RANGAP1
GSK3B
COPS5
PAK4
SNTA1
AGO2
ZNF598
MCM6
AvgSpec
0
25+
Rel AvgSpec
BFDR
≤ 0.01
≤ 0.05
> 0.05
F
mGL-TNS1 PXN
DAPI
PLL
mGL-TNS1 pPXN
DAPI
PLL

Supplementary Fig. 4.

A Representative immunoblots of TNS1-Myc-BioID expression in low- and high-expressing U2OS cells. Parental U2OS cells (CTRL) were used as control to assess endogenous TNS1 levels. Indicated molecular weight markers are in kDa.

B Quantification of relative TNS1 expression compared to control U2OS cells as in Supplementary Fig. 4A was performed from n = 3 independent replicates.

C Representative immunoblots from lysates of U2OS cells expressing BioID only, and either low or high amounts of TNS1-Myc-BioID. Cells were treated with biotin for 18 h before lysis. Indicated molecular weight markers are in kDa.

D Representative confocal images validating correct localisation of TNS1-Myc-BioID in U2OS cells with either low or high expression of TNS1-Myc-BioID. Cells were treated with biotin for 18 h, fixed and stained with fluorescently-conjugated streptavidin, anti-Myc-Tag antibody and DAPI. Scale bars 20 μm.

E High-confidence hits identified in TNS1 BioID screen. Data was visualised using ProHits Viz. Color-coding of individual spots represents the average number of unique peptides identified (AvgSpec), size of the spots reflects the relative abundance between the two conditions (Rel AvgSpec), and the color-coding of the spot outlines represents Bayesian false discovery rate (BFDR) at statistical levels as indicated.

F Representative confocal images of U2OS mGL-TNS1 cells seeded on poly-L-Lysine (PLL) and stained either with anti-PXN or anti-pPXN antibodies. Nuclei were stained using DAPI. Scale bars 20 μm.

Source data are provided as a Source Data file.

A

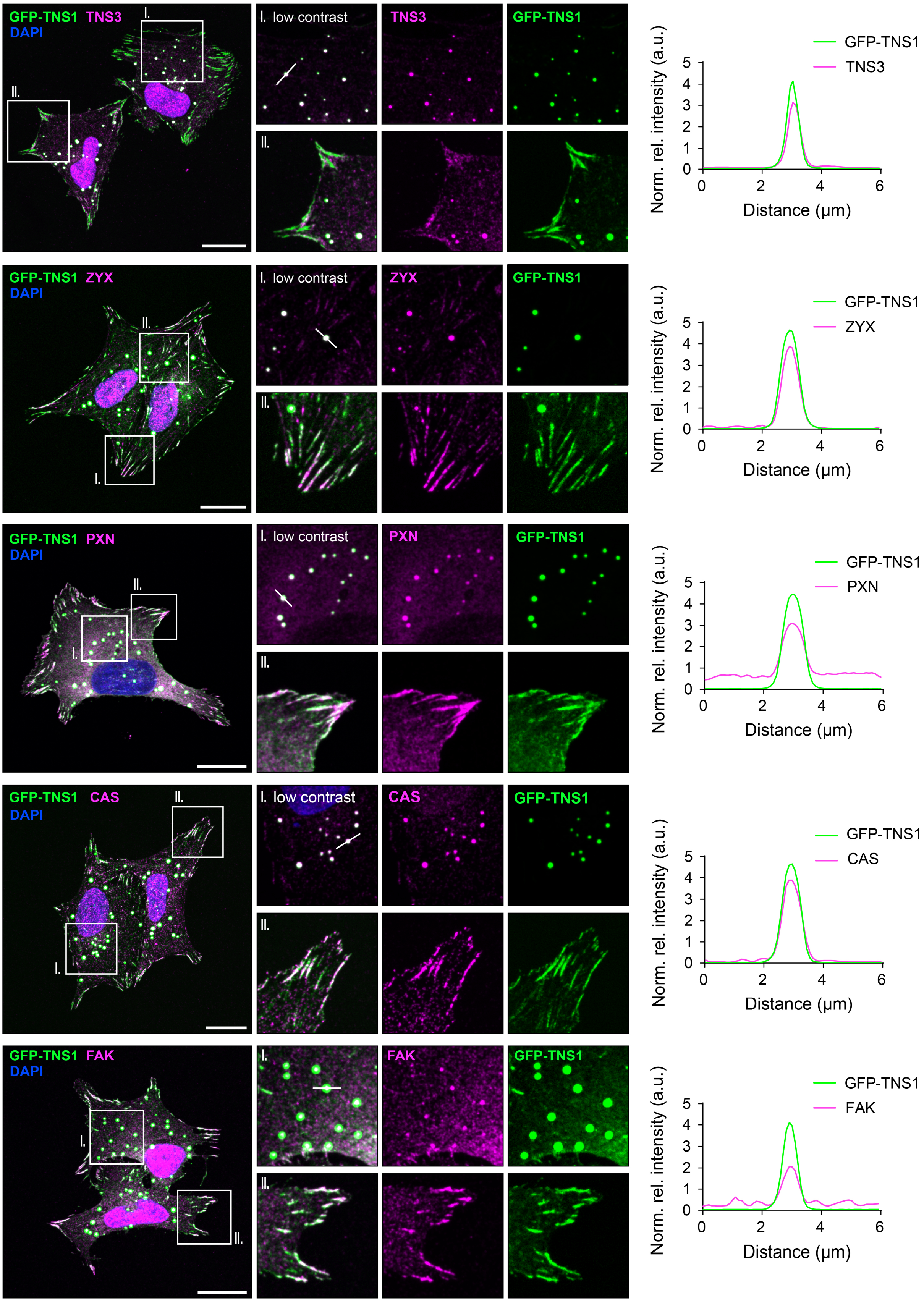

GFP-TNS1 TNS3
DAPI
I.
II.
I. low contrast
TNS3
GFP-TNS1
II.
Norm. rel. intensity (a.u.)
Distance (µm)
GFP-TNS1
TNS3
GFP-TNS1 ZYX
DAPI
I. low contrast
ZYX
ZYX
GFP-TNS1 PXN
DAPI
I. low contrast
PXN
PXN
GFP-TNS1 CAS
DAPI
I. low contrast
CAS
CAS
GFP-TNS1 FAK
DAPI
FAK
FAK

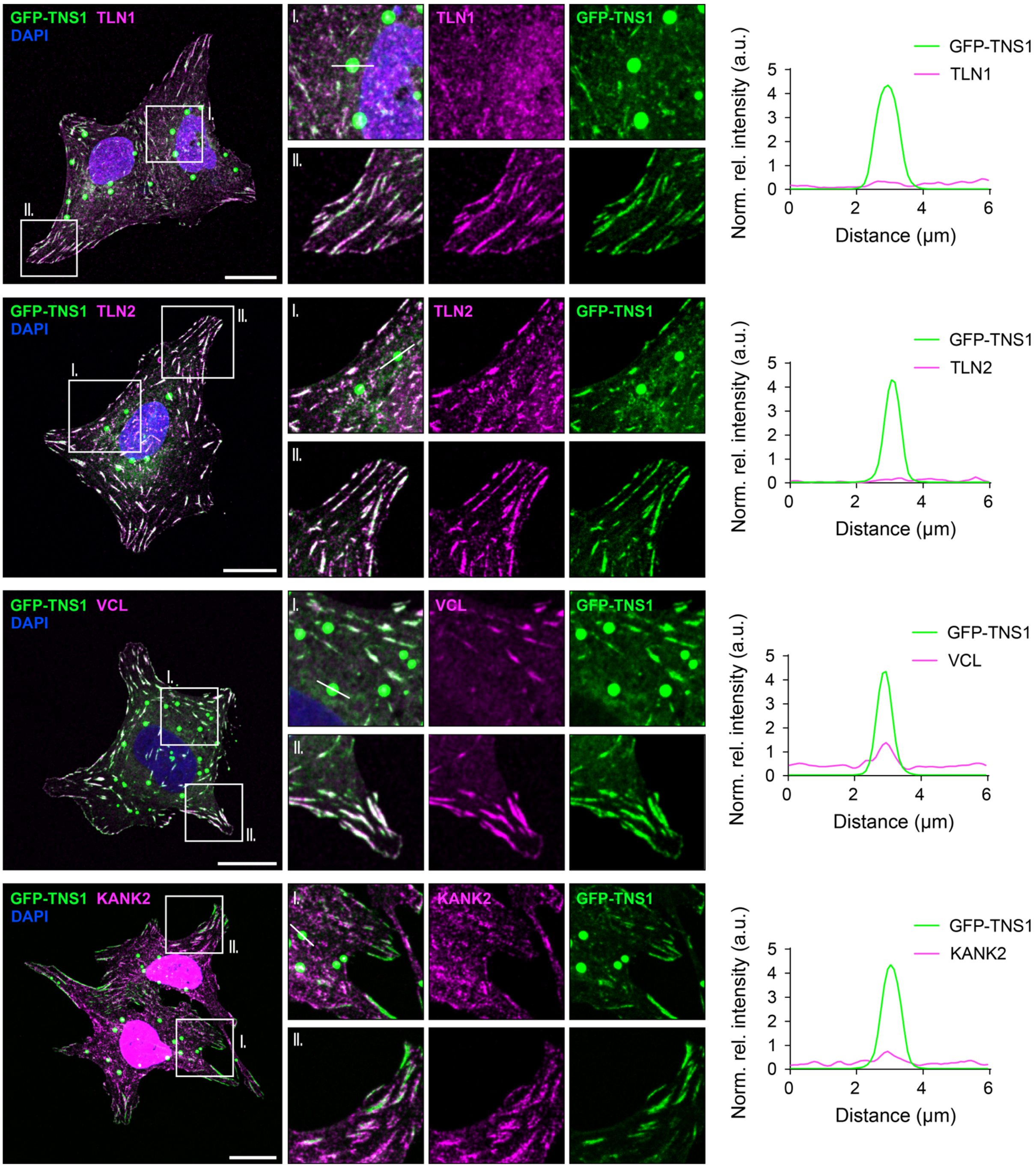
GFP-TNS1 TLN1
DAPI
I.
II.
TLN1
GFP-TNS1
Norm. rel. intensity (a.u.)
Distance (μm)
GFP-TNS1 TLN2
DAPI
TLN2
GFP-TNS1 VCL
DAPI
VCL
GFP-TNS1 KANK2
DAPI
KANK2

B

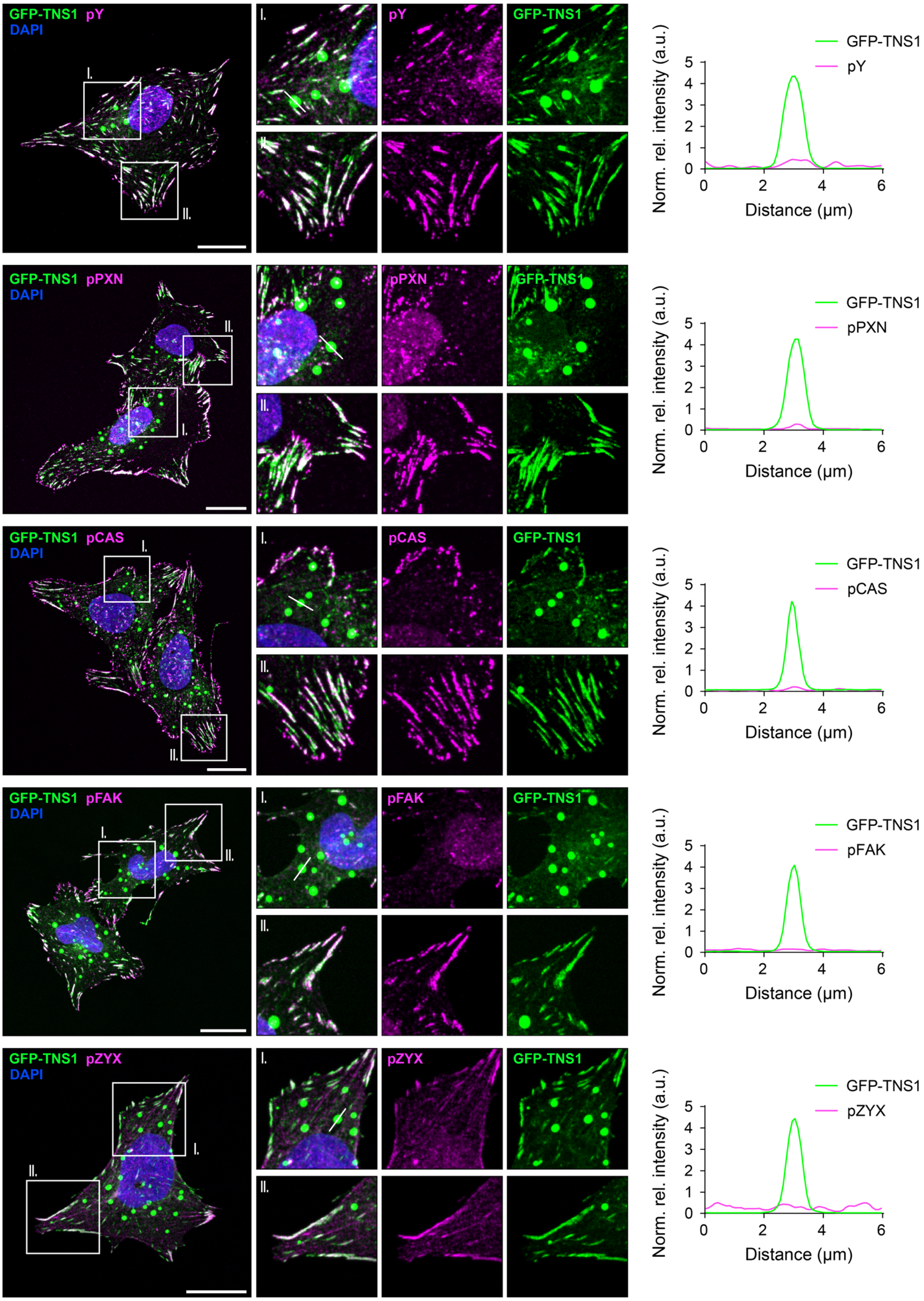

GFP-TNS1 pY
DAPI
I.
II.
pY
GFP-TNS1
GFP-TNS1
pY
Norm. rel. intensity (a.u.)
Distance (µm)
GFP-TNS1 pPXN
DAPI
pPXN
GFP-TNS1
pPXN
GFP-TNS1 pCAS
DAPI
pCAS
GFP-TNS1
pCAS
GFP-TNS1 pFAK
DAPI
pFAK
GFP-TNS1
pFAK
GFP-TNS1 pZYX
DAPI
pZYX
GFP-TNS1
pZYX

Supplementary Fig. 5.

- **A** Representative confocal images of U2OS cells expressing GFP-TNS1 stained with either anti-TNS3 (tensin 3), anti-ZYX (zyxin), anti-PXN (paxillin), anti-CAS, anti-FAK (focal adhesion kinase), anti-TLN1 (talin 1), anti-TLN2 (talin 2), anti-VCL (vinculin) or anti-KANK2 (KN motif and ankyrin repeat domains 2) antibodies. Cells were seeded overnight on FN-coated coverslips. Closeups I. focus on colocalization of GFP-TNS1 with the stained proteins in the condensates, closeups II. highlight the canonical localisation of the individual FA components in FAs. Profile plots illustrate the relative intensity distribution of individual proteins in the TNS1 condensates along the white lines indicated in closeups I. Nuclei were visualised using DAPI staining. Scale bars 20 μm. a.u. – arbitrary units.
- **B** Representative confocal images of U2OS cells expressing GFP-TNS1 stained with either anti-pY (general phosphoTyrosine), anti-pCAS, anti-pPXN, anti-pFAK or anti-pZYX antibodies. Cells were seeded overnight on FN-coated coverslips. Closeups I. focus on colocalization of GFP-TNS1 with the stained proteins in the condensates, closeups II. highlight the canonical localisation of the individual FA components in FAs. Profile plots illustrate the relative intensity distribution of individual proteins in the TNS1 condensates along the white lines indicated in closeups I. Nuclei were visualised using DAPI staining. Scale bars 20 μm. a.u. – arbitrary units.

Source data are provided as a Source Data file.

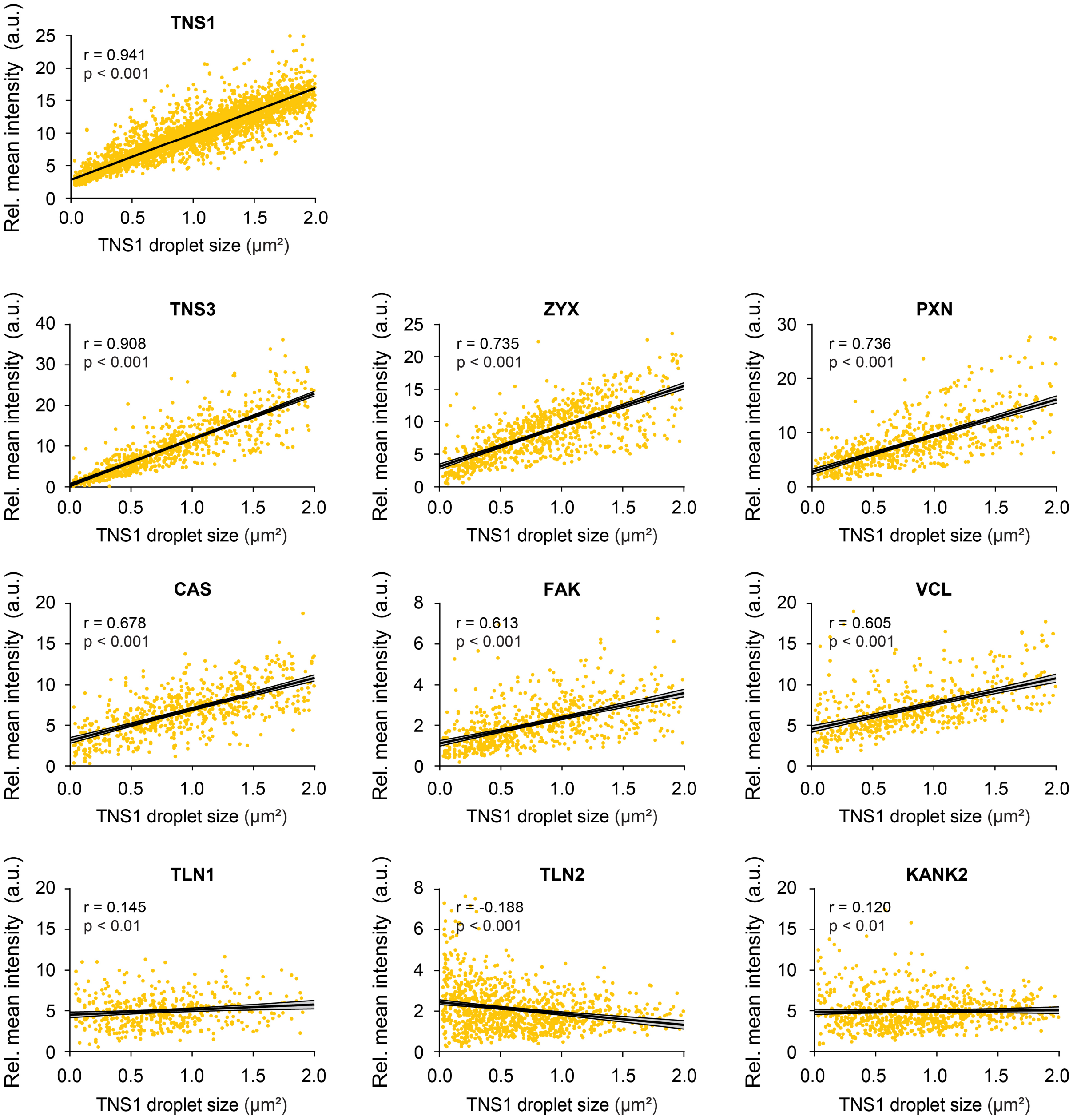

Supplementary Fig. 6.

Correlation between relative mean intensity of the indicated proteins in individual GFP-TNS1 condensates and condensate size. Quantification was performed from n = 860 (TNS3), 808 (ZYX), 649 (PXN), 636 (CAS), 676 (FAK), 527 (VCL), 442 (TLN1), 1036 (TLN2) and 717 (KANK2) individual condensates for the recruited proteins and n = 5314 condensates for TNS1. The centre line represents the fitted linear regression (mean prediction), 95% confidence bands are shown. Spearman correlation values are shown. a.u. – arbitrary units.

Source data including exact p values are provided as a Source Data file.

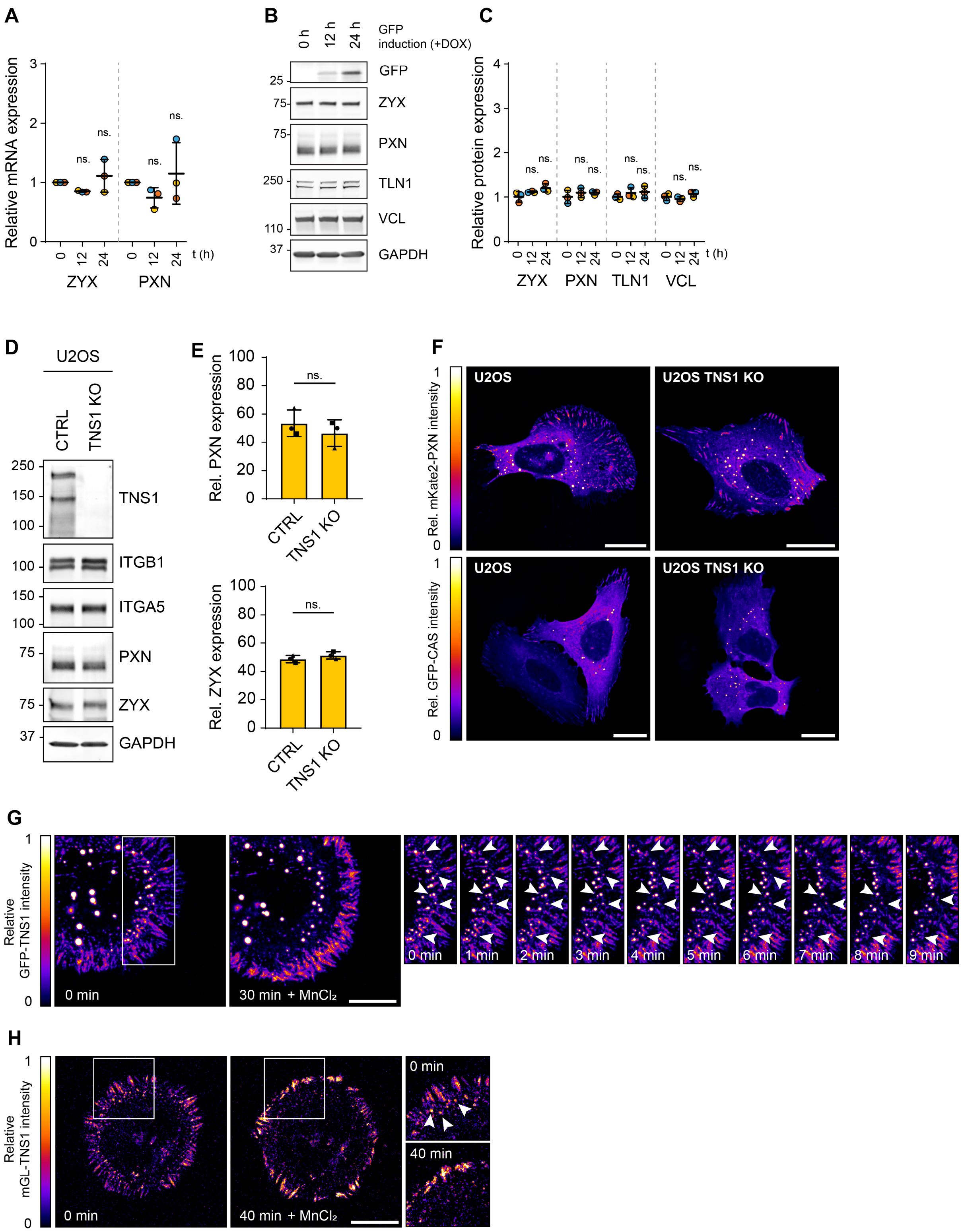

Supplementary Fig. 7.

A Relative transcription levels of ZYX and PXN in U2OS cells with induced expression of GFP-TNS1 for the indicated times. Data from 3 independent biological experiments (color-coded) are shown, normalized on GAPDH expression. Statistical analysis was performed on the ΔCt values using Friedman test with Dunn's multiple comparisons test. Indicated statistical differences are compared to protein expression at t = 0 h timepoint. Relative expression changes normalized to t = 0 h timepoint are plotted. ns. – not significant.

B Expression of GFP in U2OS cells was induced using doxycycline (DOX) for the indicated times. Representative immunoblots illustrate changes in expression levels of indicated proteins. Molecular weight markers are in kDa.

C Quantification of relative protein expression from immunoblots in Supplementary Fig. 7B, normalised to protein expression at t = 0 h. Quantification was performed from three independent experiments, data points corresponding to individual replicates are color-coded. Statistical analysis was performed using 2-way ANOVA with Holm-Šídák's multiple comparison test. Indicated statistical differences are compared to protein expression at t = 0 h timepoint. ns. – not significant.

D Representative immunoblots from lysates of control (CTRL) and TNS1 KO U2OS cells with indicated antibodies. Molecular weight markers are in kDa.

E Quantification of relative PXN (top) and ZYX (bottom) expression in U2OS CTRL and TNS1 KO cells from n = 3 independent replicates. Statistical analysis was performed using two-tailed Wilcoxon matched-pairs signed rank test. ns. – not significant.

F Representative confocal images of U2OS and U2OS TNS1 KO cells transfected with the indicated constructs. Scale bars 20 µm.

G Representative time-lapse confocal images of U2OS GFP-TNS1 cells seeded on PLL and monitored at 1 min intervals upon integrin activation by 3 mM $MnCl_2$. Some of the disassembling condensates are indicated by arrowheads. Scale bar 10 µm.

H Representative confocal images of U2OS mGL-TNS1 cells seeded on PLL-coated coverslips before (t = 0 min) and after (t = 40 min) administration of $MnCl_2$ (final concentration 3 mM). Arrowheads indicate mGL-TNS1 condensates. Scale bar 10 µm.

Source data including exact p values are provided as a Source Data file.

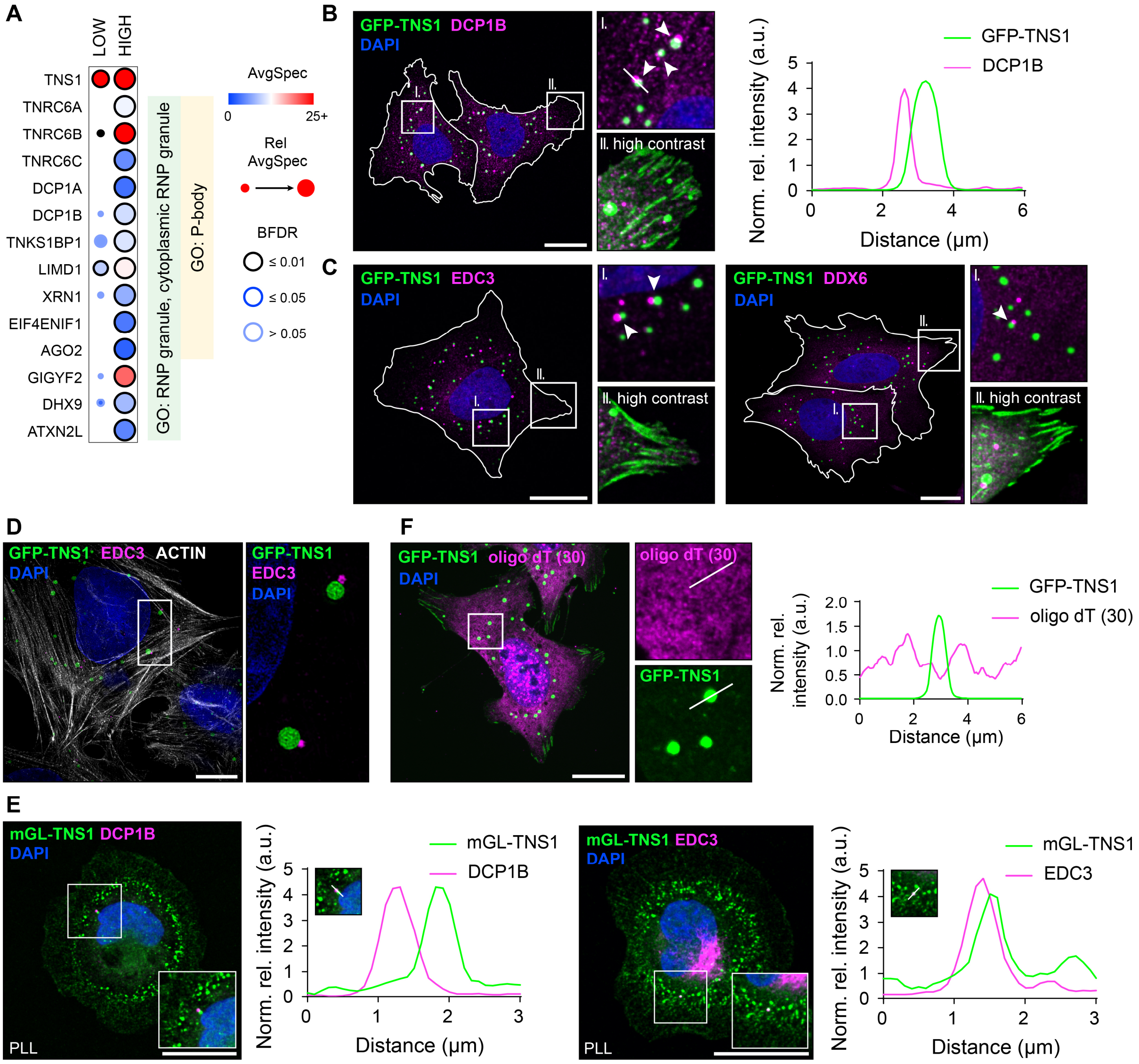

Supplementary Fig. 8.

- **A** High-confidence hits identified in TNS1 BioID screen related to RNP granule, cytoplasmic RNP granule (green) and P-body (yellow) GO terms. Data was visualised using ProHits Viz. Color-coding of individual spots represents the average number of unique peptides identified (AvgSpec), size of the spots reflects the relative abundance between the two conditions (Rel AvgSpec), and the color-coding of the spot outlines represents Bayesian false discovery rate (BFDR) at statistical levels as indicated.
- **B** Representative confocal image of U2OS cells expressing GFP-TNS1 stained with anti-DCP1B (mRNA-decapping enzyme 1B) antibody and DAPI. Closeup I. focused on association of TNS1 condensates with P-bodies (white arrowheads), closeup II. highlights the canonical TNS1 localisation in FAs. Profile plot (right) illustrates the relative intensity distribution along the white line indicated in closeup I. Scale bar 20 μm. a.u. – arbitrary units.
- **C** Representative confocal images of U2OS cells expressing GFP-TNS1. Fixed cells were stained with DAPI and either anti-EDC3 (left) or anti-DDX6 (right) antibodies. Closeups I. focus on association of TNS1 condensates with P-bodies (white arrowheads), closeups II. highlight the canonical TNS1 localisation in FAs. Scale bars 20 μm.
- **D** Representative superresolution image (SR-SIM) of U2OS cells expressing GFP-TNS1 stained with anti-EDC3 antibody, phalloidin (F-actin) and DAPI. Maximum intensity projection is shown. Closeup represents a single focal plane. Scale bar 10 μm.

E Representative confocal images of U2OS mGL-TNS1 cells seeded on PLL-coated coverslips. Cells were stained with anti-DCP1B (left) and -EDC3 (right) antibodies. Intensity plots across the respective lines are presented. Scale bars 20 µm. a.u. – arbitrary units.

F Representative confocal image of mRNA-FISH (fluorescence *in situ* hybridisation) with oligo-dT(30) probes in U2OS cells expressing GFP-TNS1. Nuclei were visualised using DAPI staining. Profile plot (bottom) illustrates the relative intensity distribution along the white line indicated in closeups. Scale bar 20 µm. a.u. – arbitrary units.

Source data are provided as a Source Data file.

A

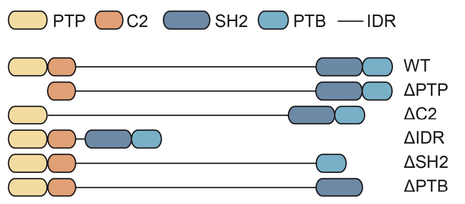

B

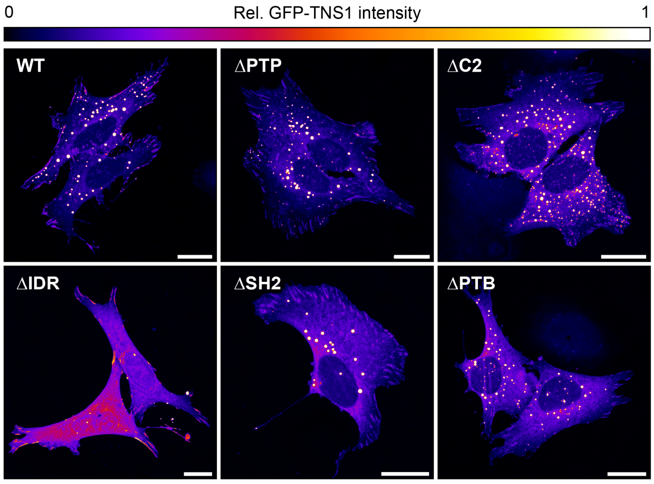

C

TNS1	Spearman r	*p*-value	slope	SE	covariance *p*-value (to WT)
WT	0.792	<0.001	47.94	1.187	N/A
ΔIDR	0.744	<0.001	9.16	0.654	<0.001
ΔIDR1	0.875	<0.001	45.29	1.178	0.128
ΔIDR2	0.816	<0.001	33.45	1.108	<0.001
ΔIDR3	0.670	<0.001	24.72	1.303	<0.001

D

Metric	Description	IDR	ΔIDR1	ΔIDR2	ΔIDR3	IDR1	IDR2	IDR3
f_k	Fraction of lysine residues	0.022	0.020	0.019	0.024	0.023	0.025	0.016
f_r	Fraction of arginine residues	0.054	0.050	0.053	0.054	0.057	0.049	0.049
f_e	Fraction of glutamic acid residues	0.050	0.042	0.043	0.067	0.047	0.068	0.016
f_d	Fraction of aspartic acid residues	0.030	0.022	0.035	0.034	0.047	0.022	0.023
f_{aro}	Fraction of aromatic residues	0.050	0.050	0.053	0.044	0.047	0.038	0.059
$\langle\lambda\rangle$	Average disorder tendency	0.445	0.451	0.450	0.431	0.432	0.430	0.468
SHD	Sequence hydrophobicity disorder	5.860	5.580	5.611	5.332	4.772	4.721	5.206
Ω_{aro}	Clustering of aromatic residues	1.992	1.923	1.821	2.139	1.795	2.005	1.550
SCD	Sequence charge decoration	-2.200	-0.943	-2.098	1.926	2.056	0.247	0.899
κ	Charge asymmetry	0.166	0.170	0.161	0.167	0.158	0.180	0.157
FCR	Fraction of charged residues	0.157	0.138	0.153	0.182	0.203	0.169	0.109
NCPR	Net charge per residue	-0.005	0.007	-0.005	-0.022	-0.036	-0.016	0.026

E

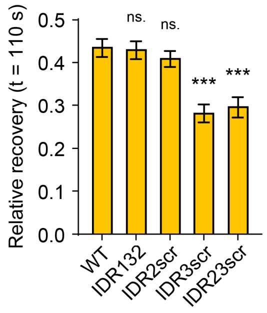

Supplementary Fig. 9.

- **A** Schematic representation of domain organisation of TNS1 WT and its individual deletion mutants.
- **B** Representative confocal images of U2OS TNS1 KO cells expressing indicated GFP-TNS1 variants. Scale bars 20 µm.
- **C** Analysis of data from n = 114 (WT), 134 (ΔIDR), 121 (ΔIDR1), 90 (ΔIDR2) and 168 (ΔIDR3) cells shown in Fig. 4C was performed using linear regression analysis. Slope and standard error (SE) for each TNS1 variant are shown. Spearman correlation (r) was calculated for each TNS1 variant, Spearman r and the respective p-values are shown. Statistical differences between slopes were assessed by covariance analysis comparing each of the deletion mutants to TNS1 WT.
- **D** Compositional analysis of the IDR sequence, its individual deletion mutants and subregions.

E Quantification of relative fluorescence recovery shown in Fig. 4H-I ($n = 40$ (WT), 41 (IDR132), 39 (IDR2scr), 40 (IDR3scr) and 31 (IDR23scr) condensates) at $t = 110$ s after photobleaching. Statistical analysis was performed using one-way ANOVA test with Tukey's multiple comparisons test against WT. $p < 0.001$ (***).

Source data including exact p values are provided as a Source Data file.

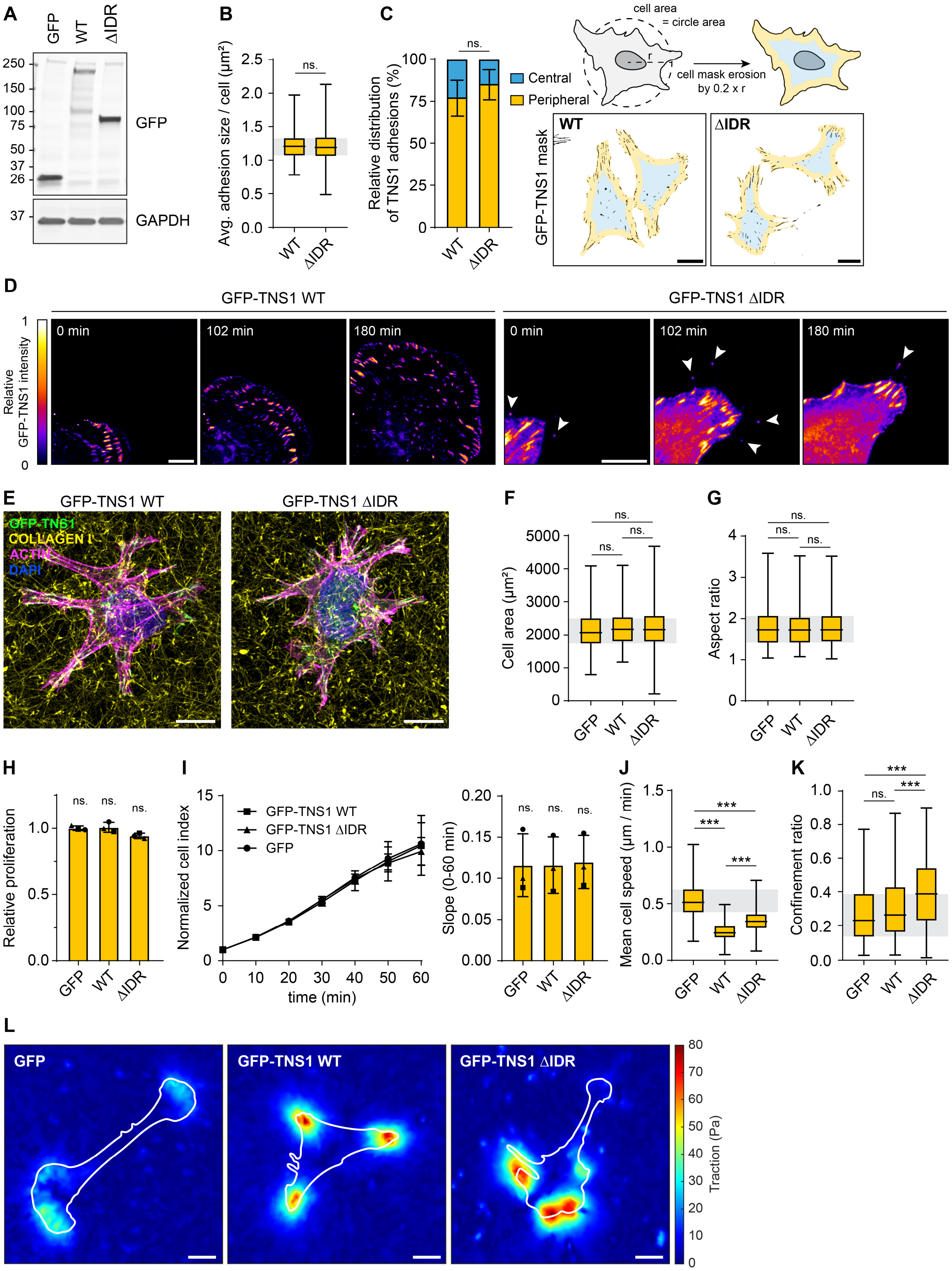

A
GFP
WT
ΔIDR
250
150
100
75
50
37
26
GFP
37
GAPDH
B
Avg. adhesion size / cell (µm²)
ns.
WT
ΔIDR
C
Relative distribution of TNS1 adhesions (%)
ns.
Central
Peripheral
cell area = circle area
r
cell mask erosion by 0.2 x r
GFP-TNS1 mask
WT
ΔIDR
D
GFP-TNS1 WT
GFP-TNS1 ΔIDR
Relative GFP-TNS1 intensity
0 min
102 min
180 min
E
GFP-TNS1 WT
GFP-TNS1 ΔIDR
GFP-TNS1
COLLAGEN I
ACTIN
DAPI
F
Cell area (µm²)
ns.
G
Aspect ratio
ns.
H
Relative proliferation
ns.
I
Normalized cell index
GFP-TNS1 WT
GFP-TNS1 ΔIDR
GFP
time (min)
Slope (0-60 min)
J
Mean cell speed (µm / min)
***
K
Confinement ratio
ns.
***
L
GFP
GFP-TNS1 WT
GFP-TNS1 ΔIDR
Traction (Pa)

Supplementary Fig. 10.

A Representative immunoblots of U2OS TNS1 KO cells with inducible expression of either GFP or the indicated GFP-TNS1 variants. Molecular weight markers are in kDa.

B Quantification of the average FA size per cell from n = 165 (WT) and 155 (ΔIDR) cells from three independent experiments.

C Quantification of relative distribution of peripheral and central FAs from the indicated cell lines. Analysis was performed from n = 137 (WT) and 117 (ΔIDR) cells by eroding the cell mask by the factor of 0.2 × radius (r) of a circle with area corresponding to the area of the cell. Representative images corresponding to central and peripheral areas with GFP-TNS1 adhesion masks are shown. Scale bars 20 μm.

D Representative time-lapse images of cell protrusions of indicated cell lines migrating on FN-coated coverslips. Images were acquired for 3 h at 3 min intervals. White arrowheads are indicating TNS1-containing filopodia tips. Scale bars 10 μm.

E Representative 3D projection of cells expressing indicated GFP-TNS1 variants embedded in 3D fibrillar rat tail collagen I conjugated with Atto647N. The cells were stained for F-actin and DAPI was used to stain cell nuclei. Scale bars 10 μm.

F Quantification of cell area of U2OS cells expressing the indicated proteins. Quantification was performed from n = 294 (GFP), 271 (WT) and 266 (ΔIDR) cells.

G Quantification of aspect ratio of U2OS cells expressing the indicated proteins. Quantification was performed from n = 290 (GFP), 271 (WT) and 262 (ΔIDR) cells.

H Quantification of relative proliferation of U2OS cells expressing indicated proteins from three independent experiments.

I Representative plot of normalized cell index as measured by xCELLigence (left). Mean values of 3 technical replicates with the corresponding standard deviations are shown. Slopes of each condition from three independent biological replicates are shown (right).

J Quantification of mean cell speed of cells analysed in Fig. 5F (n = 170 (GFP), 159 (WT) and 161 (ΔIDR)).

K Quantification of confinement ratio of cells analysed in Fig. 5F (n = 170 (GFP), 159 (WT) and 161 (ΔIDR)).

L Representative traction map images of indicated cells seeded on 10 kPa gels. Cell outlines are shown with white line. Scale bars 20 μm.

Statistical analysis was performed using two-tailed Mann-Whitney U test (**B**), 2-way ANOVA test (C), Kruskal-Wallis test with Dunn's multiple comparisons test (**F**-**H**, **J**-**K**) and Friedman test with Dunn's multiple comparisons test (**I**). $p < 0.001$ (***); ns. – not significant.
Source data including exact p values are provided as a Source Data file.

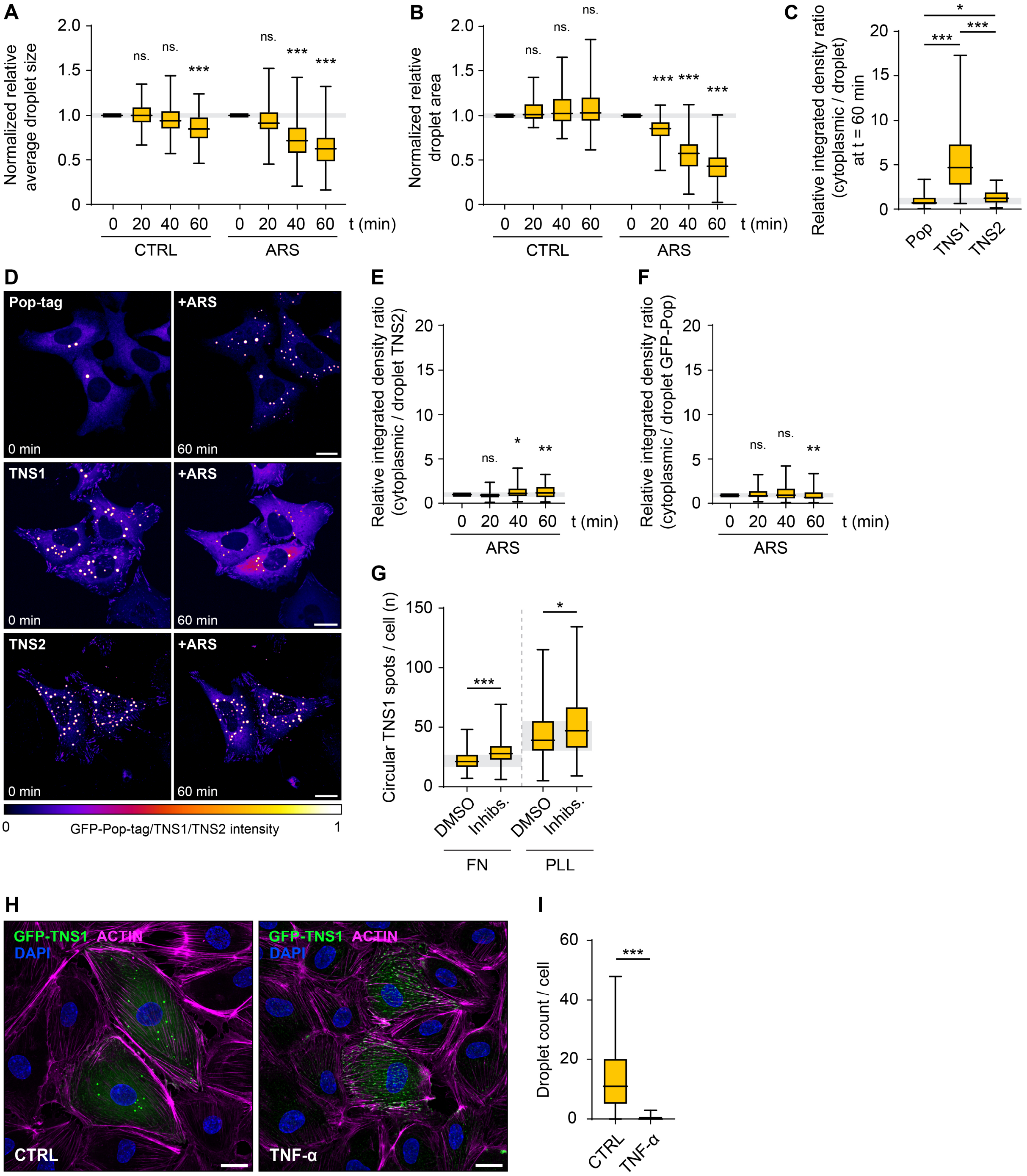

Supplementary Fig. 11.

- **A** Quantification of normalized relative average TNS1 droplet size from n = 81 (CTRL) and 119 (ARS) cells analysed in Fig. 6A-B.
- **B** Quantification of normalized relative TNS1 droplet area from n = 80 (CTRL) and 117 (ARS) cells analysed in Fig. 6A-B.
- **C** Quantification of the ratio between the relative cytoplasmic and droplet integrated densities of GFP-Pop, GFP-TNS1 and GFP-TNS2 at t = 60 min from n = 104 (Pop), 115 (TNS1) and 112 (TNS2) cells.

D Representative timelapse images of ARS-treated U2OS cells expressing either GFP-Pop, GFP-TNS1 or GFP-TNS2. Images were acquired for 60 min at 1 min intervals. Images at t = 0 min and t = 60 min are shown. Scale bars 20 μm.

E Quantification of the ratio between the relative cytoplasmic and droplet GFP-TNS2 integrated densities at the indicated timepoints from n = 112 cells.

F Quantification of the ratio between the relative cytoplasmic and droplet GFP-Pop integrated densities at the indicated timepoints from n = 104 cells.

G Quantification of the number of circular TNS1 spots from U2OS mGL-TNS1 cells seeded on FN- or PLL-coated coverslips (n = 109 (FN-DMSO), 106 (FN-Inhibs.), 97 (PLL-DMSO), 83 (PLL-Inhibs.)). 2 h after seeding, the cells were treated for 60 min with either DMSO or a combination of 5 μM Doramapimod (p38), 5 μM Trametinib (MEK1/2) and 5 μM MK2206 (Akt) (Inhibs.).

H Representative confocal images of HUVEC cells treated with either control or 5 ng/ml TNF-α for 2.5 h. Scale bars 20 μm.

I Quantification of the number of GFP-TNS1 condensates in HUVEC cells treated with either control or 5 ng/ml TNF-α from Supplementary Fig. 11H. Quantification was performed from n = 78 (CTRL) and 77 (TNF-α) cells.

Statistical analysis was performed using Friedman test with Dunn's multiple comparisons test comparing the individual timepoints to t = 0 min (**A**-**B**, **E**-**F**), Kruskal-Wallis test with Dunn's multiple comparisons test (**C**) and two-tailed Mann-Whitney U test (**G**, **I**). $p < 0.05$ (*); $p < 0.01$ (**); $p < 0.001$ (***); ns. – not significant.
Source data including exact p values are provided as a Source Data file.

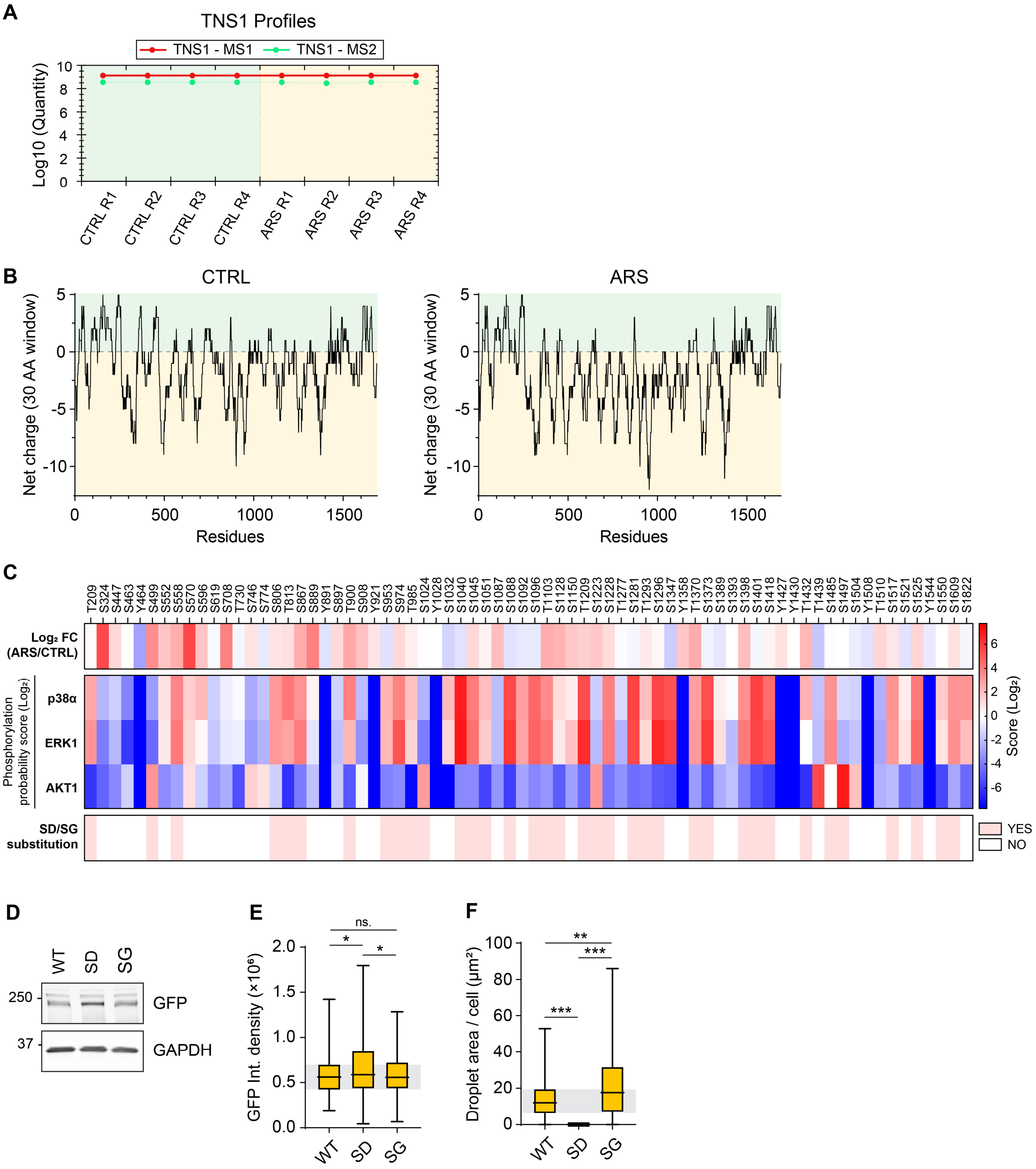

Supplementary Fig. 12.

A Plot of normalized TNS1 quantities identified by mass spectrometry.

B Cumulative net charge distribution over 30 AA window in TNS1 from CTRL (left) or ARS-treated (right) cells.

C Heatmap of phosphorylation probability score for individual phosphorylation sites by p38α, ERK1 and AKT1 kinases as predicted by Kinase Library tool[67]. Top panel represents Log_2 fold-change of individual TNS1 phosphosites in CTRL and ARS-treated conditions. Bottom panel (SD/SG substitution) indicates phosphorylation sites selected for experimental validation.

D Representative immunoblots from lysates of U2OS cells with inducible expression of the indicated GFP-TNS1 variants. Molecular weight markers are in kDa.

E Quantification of integrated density of cells analysed in Fig. 6J-L and Supplementary Fig. 12F (n = 339 (WT), 371 (SD) and 351 (SG) cells).

F Quantification of TNS1 droplet area per cell as quantified from n = 343 (WT), 358 (SD) and 354 (SG) cells.

Statistical analysis was performed using Kruskal-Wallis test with Dunn's multiple comparisons test (**E**-**F**). $p < 0.05$ (*); $p < 0.01$ (**); $p < 0.001$ (***); ns. – not significant.
Source data including exact p values are provided as a Source Data file.

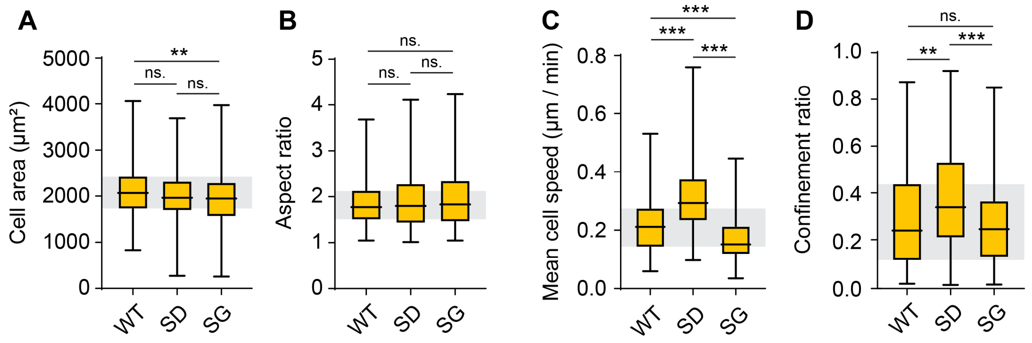

Supplementary Fig. 13.

A Quantification of cell area from n = 339 (WT), 374 (SD) and 355 (SG) cells.

B Quantification of cell aspect ratio from n = 331 (WT), 367 (SD) and 351 (SG) cells.

C Quantification of mean cell speed of cells analysed in Fig. 7F. n = 136 (WT), 153 (SD) and 135 (SG) cells.

D Quantification of confinement ratio of cells analysed in Fig. 7F. n = 137 (WT), 153 (SD) and 135 (SG) cells.

Statistical analysis was performed using Kruskal-Wallis test with Dunn's multiple comparisons test (**A**-**D**). $p < 0.01$ (**); $p < 0.001$ (***); ns. – not significant.
Source data including exact p values are provided as a Source Data file.

Supplementary Table 1

Table indicating reagent ratios for desired hydrogel stiffness.

~Young's modulus (kPa)	PBS (µl)	Acrylamide (µl)	Bis-acrylamide (µl)	Final acrylamide %	Final bis-acrylamide %
0.5	397	63	10	5.4	0.04
2	365	63	17.5	5.7	0.08
10	356	94	50	7.5	0.2
20	300	150	50	12	0.2
