# Supplementary material for: Adhesion-derived condensates control component availability to regulate adhesion dynamics
Source: Nat Commun. 2026 Jun 5;17:7222. doi: 10.1038/s41467-026-74001-3 (PMC13396368; doi:10.1038/s41467-026-74001-3)
Supplement: Supplementary file 2 — Description of Additional Supplementary Files [file 41467_2026_74001_MOESM2_ESM.pdf]

Description of Additional Supplementary Files

File name: Supplementary Data 1
Description: Document file containing the list of synthesized DNA sequences and oligonucleotides used in this study.

File name: Supplementary Data 2
Description: Excel file containing processed MS data from BioID experiment. Related to Fig. 3.

File name: Supplementary Data 3
Description: Excel file containing processed TNS1 phosphoproteomic data. Statistical significance assessed by unpaired t-test. Related to Fig. 6.

File name: Supplementary Video 1
Description: Fusion of GFP-TNS1 condensates. Related to Fig. 1C.

File name: Supplementary Video 2
Description: Fission of GFP-TNS1 condensates. Related to Fig. 1D.

File name: Supplementary Video 3
Description: TNS1 condensate formation upon focal adhesion disassembly, related to Supplementary Fig. 1C.

File name: Supplementary Video 4
Description: GFP-TNS1 dynamics in living cells. Related to Supplementary Fig. 1D.

File name: Supplementary Video 5
Description: Representative FRAP time-lapse. Related to Fig. 1E.

File name: Supplementary Video 6
Description: Time-lapse of U2OS GFP-TNS1 cells seeded on PLL, with subsequent integrin activation by $MnCl_2$. Scale bar 10 μm. Related to Supplementary Fig. 7G.

File name: Supplementary Video 7
Description: Time-lapse of control U2OS GFP-TNS1 cells. Scale bar 20 μm. Related to Fig. 6A.

File name: Supplementary Video 8
Description: Time-lapse of arsenite-treated U2OS GFP-TNS1 cells. Scale bar 20 μm. Related to Fig. 6A.

File name: Supplementary Video 9
Description: Time-lapse of U2OS GFP-TNS1 cells pre-treated with inhibitors before the arsenite treatment. Scale bar 20 μm. Related to Fig. 6E.
