# Supplementary material for: Adhesion-derived condensates control component availability to regulate adhesion dynamics
Source: Nat Commun. 2026 Jun 5;17:7222. doi: 10.1038/s41467-026-74001-3 (PMC13396368; doi:10.1038/s41467-026-74001-3)
Supplement: Supplementary file 3 — Supplementary Data 1 [file 41467_2026_74001_MOESM3_ESM.docx]

**Supplementary information**

**Supplementary Table 1**

**List of synthetic DNA sequences and primers used in the study**

TNS1 SD_1

GTACAAGTCCGGACTCAGATCTCGAGCTCAAGCTTACATGAGTGTGAGCCGGACCATGGACAGCTGTGAGCTGGACCTGGTGTACGTCACAGAGAGGATCATCGCTGTCTCCTTCCCCAGCACAGCCAATGAGGAGAACTTCCGGAGCAACCTCCGTGAGGTGGCGCAGATGCTCAAGTCCAAACATGGAGGCAACTACCTGCTGTTCAACCTCTCTGAGCGGAGACCTGACATCACGAAGCTCCATGCCAAGGTACTGGAATTTGGCTGGCCCGACCTCCACGATCCAGCCCTGGAGAAGATCTGCAGCATCTGTAAGGCCATGGACACATGGCTCAATGCAGACCCTCACAATGTCGTTGTTCTACACAACAAGGGAAACCGAGGCAGGATAGGAGTTGTCATCGCGGCTTACATGCACTACAGCAACATTTCTGCCAGTGCGGACCAGGCTCTGGACCAGTTTGCAATGAAGCGGTTCTATGAGGATAAGATTGTGCCCATTGGCCAGCCATCCCAAAGAAGGTACGTGCATTACTTCAGTGGCCTGCTCTCCGGCTCCATCAAAATGAACAACAAGCCCTTGTTTCTGCACCACGTGATCATGCACGGCATCCCCAACTTTGAGTCTAAAGGAGGATGTCGGCCATTTCTCCGCATCTACCAGGCCATGCAACCTGTGTACACATCTGGCATCTACAACATCCCAGGAGACAGCCAGACTAGCGTCTGCATCACCATCGAGCCAGGACTGCTCTTGAAGGGAGACATCTTGCTGAAGTGCTACCACAAGAAGTTCCGAAGCCCAGCCCGAGACGTCATCTTCCGTGTGCAGTTCCACACCTGTGCCATCCATGACCTGGGGGTTGTCTTTGGGAAGGAGGACCTTGATGATGCTTTCAAAGATGATCGATTTCCAGAGTATGGCAAAGTGGAGTTTGTATTTTCTTATGGGCCAGAGAAAATTCAAGGCATGGAGCACCTGGAGAACGGGCCGAGCGTGTCTGTGGACTATAACACCTCTGACCCCCTCATCCGCTGGGACTCCTACGACAACTTCAGTGGGCATCGAGATGACGGCATGGAGGAGGTGGTGGGACACACGCAGGGGCCACTAGATGGGAGCCTGTATGCTAAGGTGAAGAAGAAAGACGATCTGCACGGCAGCACCGGGGCTGTTAATGCCACACGTCCTACACTGTCGGCCACCCCCAACCACGTGGAACACACGCTTTCTGTGAGCAGCGACTCGGGCAACTCCACAGCCTCCACCAAGACCGACAAGACCGACGAGCCTGTCCCCGGGGCCTCCAGTGCCACTGCTGCCTTGGATCCCCAGGAGAAGCGGGAGCTGGACCGCCTGCTGAGTGGCTTTGGCTTAGAGCGAGAGAAGCAAGGCGCCATGTACCACACCCAGCACCTCAGGTCCCGCCCAGCAGGGGGCTCGGCTGTGCCCTCCTCTGGACGCCACGTTGTCCCAGCCCAGGTTCATGTCAATGGTGGGGCGTTAGCATCTGAGCGGGAGACAGACATCCTGGACGATGAATTGCCAAACCAGGATGGTCACAGTGCGGGCAGCATGGGCACACTCTCTTCTCTGGACGGGGTCACCAACACCAGTGAGGGGGGCTACCCAGAGGCCCTGTCCCCACTGACCAACGGTCTGGACAAGTCCTACCCCATGGAGCCTATGGTCAATGGAGGAGGCTACCCCTACGAGTCTGCCAGCCGGGCGGGGCCTGCCCATGCTGGCCACACGGCCCCCATGCGGCCCTCCTACTCTGCACAGGAGGGTTTAGCTGGCTACCAGAGGGAGGGGCCCCACCCAGCCTGGCCACAGCCAGTGACCACCTCCCACTATGCCCATGACCCCAGCGGTATGTTCCGCTCTCAATCCTTTTCGGAAGCTGAACCCCAGCTGCCCCCAGCTCCGGTCCGAGGGGGAAGCAGCCGGGAGGCTGTGCAAAGGGGACTGAATTCATGGCAGCAGCAGCAGCAGCAGCAGCAGCAGCCTCGCCCACCTCCACGCCAGCAGGAAAGAGCCCACTTGGAGAGTCTTGTAGCCAGCAGGCCCGATCCTCAGCCATTGGCAGAGGATCCCATCCCCAGTCTCCCTGAGTTCCCGCGAGCAGCCTCCCAGCAGGAGATTGAACAGTCCATCGAAACACTCAATATGCTGATGCTGGACCTGGAGCCAGCCTCCGCTGCTGCCCCACTACACAAGTCCCAGAGTGTCCCCGGGGCCTGGCCAGGGGCTGATCCACTCTCCTCCCAGCCCCTCTCTGGATCCTCCCGTCAGTCCCATCCACTGACCCAGTCCAGATCTGGCTATATCCCCAGTGGGCATTCGTTGGGAGATCCTGAGCCAGCCCCACGGGCCTCTCTGGAGTCTGTCCCTCCTGGCAGGTCTTACTCACCTTATGACTATCAGCCATGTTTGGCTGGGCCTAACCAGGATTTCCATTCAAAGAGCCCAGCCTCTTCCTCCTTGCCTGCCTTCCTTCCGACCACCCACGATCCTCCAGGGCCTCAGCAACCCCCAGCCTCTCTCCCTGGCCTCACTGCTCAGCCTCTGCTCGATCCAAAGGAAGCGACTTCAGACCCCTCCCGGGATCCAGAGGAGGAGCCATTGAATTTAGAAGGGCTGGTGGCCCACAGGGTAGCAGGGGTACAGGCTCGGGAGAAGCAGCCTGCAGAGCCCCCAGCCCCTCTGCGGAGGCGGGCGGCCGATGATGGACAGTATGAGAACCAGTCTCCAGAAGCCACATCCCCTCGTGATCCTGGGGTTCGCGATCCTGTCCAGTGTGTCGATCCGGAGCTGGCTCTTACCATCGCTCTCAATCCTGGAGGGCGGCCCAAAGAGCCCCATTTGCACAGCTACAAGGAGGCCTTCGAGGAGATGGAGGGAACCTCCCCGAGC

TNS1 SD_2

TGGAGGGAACCTCCCCGAGCGATCCACCACCCAGTGGGGTGCGGGATCCCCCGGGTCTGGCCAAGGATCCCCTGTCTGCTCTGGGCCTGAAACCTCACAACCCAGCGGACATCCTGTTGCACCCCACAGGAGAGCCCCGGGATTATGTGGAGTCTGTGGCACGGACAGCGGTGGCTGGACCCCGAGCTCAGGACTCTGAGCCCAAGAGCTTTAGTGCTCCAGCCACCCAGGCCTATGGCCATGAGATACCCCTGAGGAACGGGACCCTGGGTGGCTCCTTTGTCTCCCCCAGCCCCCTCTCCACCAGCAGCCCCATCCTCAGTGCTGACAGCACTTCAGTGGGGAGTTTCCCGTCGGGAGAGAGCAGTGACCAGGGTCCCCGGGATCCCACCCAGCCTCTGTTGGAGTCTGGCTTCCGCTCAGGCGATCTGGGACAGCCCGATCCATCTGCCCAGAGAAACTACCAGAGCTCTTCTCCTCTCCCGACTGTGGGCAGTAGCTACAGCAGCCCCGACTACTCACTTCAGCATTTCAGCTCCTCTCCGGAAAGCCAGGCCCGAGCTCAGTTCAGTGTGGCTGGCGTCCACACGGTGCCTGGGGATCCTCAGGCGCGCCACAGAACAGTGGGCACCAACGATCCCCCTGATCCTGGCTTCGGCCGGCGGGCCATCAATCCCAGCATGGCTGCCCCCAGCAGTCCCAGTTTGAGCCATCACCAGATGATGGGTCCACCAGGCACTGGCTTCCATGGTAGCACTGTCTCCAGCCCCCAGAGCAGTGCAGCGACCACCCCGGGGAGCCCCAGCCTGTGTCGGCACCCAGCAGGGGTCTACCAGGTTTCTGGCCTCCACAACAAAGTGGCCACCGATCCGGGGGATCCCAGCCTGGGCCGGCACCCTGGGGCTCACCAAGGCAACCTGGCCTCCGGTCTTCATAGCAATGCAATAGCCGATCCTGGAGATCCCAGCCTGGGCCGTCACCTCGGAGGGTCTGGATCTGTGGTTCCCGGCGATCCCTGCTTGGACCGGCATGTGGCCTATGGCGGCTATTCTGATCCGGAGGATCGGAGACCCACACTGTCCCGGCAGAGCAGTGCCTCTGGCTACCAGGCTCCTTCCACGCCCTCCTTCCCTGTCTCCCCTGCCTACTACCCTGGCCTGAGCAGCCCTGCCACCTCCCCGTCACCAGACTCCGCAGCCTTCCGGCAAGGGGATCCAACACCAGCCTTGCCAGAGAAGCGAAGGATGGATGTGGGAGACCGGGCAGGCAGCCTCCCCAACTATGCCACCATCAATGGGAAGGTGTCTGATCCTGTCGCCAGCGGCATGTCCGATCCCAGCGGGGGCAGCACCGTCTCCTTCTCCCACACTCTGCCCGACTTCTCCAAGTACTCCATGCCAGACAACGATCCGGAGACGCGGGCTAAAGTGAAGTTTGTCCAGGACACTTCTAAGTATTGGTACAAGCCTGAGATCTCCAGGGAGCAGGCCATCGCGCTCCTCAAGGACCAGGAGCCGGGGGCCTTCATCATCCGCGACAGTCACTCCTTCCGAGGCGCGTACGGGCTGGCCATGAAGGTGTCTGATCCACCTCCAACCATCATGCAGCAGAATAAAAAAGGAGACATGACCCATGAGCTGGTCAGGCATTTTCTGATAGAGACTGGCCCCAGAGGAGTCAAGCTCAAGGGCTGCCCCAATGAGCCAAACTTCGGATCGCTGTCTGCCCTGGTCTACCAGCACTCCATCATCCCATTGGCCCTGCCTTGCAAGCTGGTCATTCCAAACCGAGACCCCACAGATGAATCGAAAGATAGCTCCGGCCCTGCCAACTCAACTGCAGACCTGCTGAAACAAGGGGCAGCCTGCAATGTGCTCTTCATCAACTCTGTGGACATGGAGTCACTCACTGGGCCACAGGCCATCTCTAAAGCCACATCTGAGACGTTGGCTGCAGACCCCACACCAGCTGCCACCATCGTTCACTTCAAAGTCTCTGCCCAGGGAATCACTCTGACTGACAACCAGAGAAAGCTCTTTTTCAGACGCCACTACCCTCTCAACACTGTCACCTTCTGTGACCTGGATCCACAGGAAAGAAAGTGGATGAAAACAGAGGGTGGTGCCCCTGCTAAGCTCTTCGGCTTCGTGGCCCGGAAGCAGGGCAGCACCACGGACAACGCCTGCCACCTCTTTGCTGAGCTTGACCCCAACCAGCCGGCCTCTGCCATCGTCAACTTCGTCTCCAAGGTCATGCTGAATGCCGGCCAAAAGAGATGAGTCGACGGTACCGCGGGCCCGGGAT

TNS1 SG_1

GTACAAGTCCGGACTCAGATCTCGAGCTCAAGCTTACATGAGTGTGAGCCGGACCATGGACAGCTGTGAGCTGGACCTGGTGTACGTCACAGAGAGGATCATCGCTGTCTCCTTCCCCAGCACAGCCAATGAGGAGAACTTCCGGAGCAACCTCCGTGAGGTGGCGCAGATGCTCAAGTCCAAACATGGAGGCAACTACCTGCTGTTCAACCTCTCTGAGCGGAGACCTGACATCACGAAGCTCCATGCCAAGGTACTGGAATTTGGCTGGCCCGACCTCCACGGACCAGCCCTGGAGAAGATCTGCAGCATCTGTAAGGCCATGGACACATGGCTCAATGCAGACCCTCACAATGTCGTTGTTCTACACAACAAGGGAAACCGAGGCAGGATAGGAGTTGTCATCGCGGCTTACATGCACTACAGCAACATTTCTGCCAGTGCGGACCAGGCTCTGGACCAGTTTGCAATGAAGCGGTTCTATGAGGATAAGATTGTGCCCATTGGCCAGCCATCCCAAAGAAGGTACGTGCATTACTTCAGTGGCCTGCTCTCCGGCTCCATCAAAATGAACAACAAGCCCTTGTTTCTGCACCACGTGATCATGCACGGCATCCCCAACTTTGAGTCTAAAGGAGGATGTCGGCCATTTCTCCGCATCTACCAGGCCATGCAACCTGTGTACACATCTGGCATCTACAACATCCCAGGAGACAGCCAGACTAGCGTCTGCATCACCATCGAGCCAGGACTGCTCTTGAAGGGAGACATCTTGCTGAAGTGCTACCACAAGAAGTTCCGAAGCCCAGCCCGAGACGTCATCTTCCGTGTGCAGTTCCACACCTGTGCCATCCATGACCTGGGGGTTGTCTTTGGGAAGGAGGACCTTGATGATGCTTTCAAAGATGATCGATTTCCAGAGTATGGCAAAGTGGAGTTTGTATTTTCTTATGGGCCAGAGAAAATTCAAGGCATGGAGCACCTGGAGAACGGGCCGAGCGTGTCTGTGGACTATAACACCTCTGACCCCCTCATCCGCTGGGACTCCTACGACAACTTCAGTGGGCATCGAGATGACGGCATGGAGGAGGTGGTGGGACACACGCAGGGGCCACTAGATGGGAGCCTGTATGCTAAGGTGAAGAAGAAAGACGGACTGCACGGCAGCACCGGGGCTGTTAATGCCACACGTCCTACACTGTCGGCCACCCCCAACCACGTGGAACACACGCTTTCTGTGAGCAGCGACTCGGGCAACTCCACAGCCTCCACCAAGACCGACAAGACCGACGAGCCTGTCCCCGGGGCCTCCAGTGCCACTGCTGCCTTGGGACCCCAGGAGAAGCGGGAGCTGGACCGCCTGCTGAGTGGCTTTGGCTTAGAGCGAGAGAAGCAAGGCGCCATGTACCACACCCAGCACCTCAGGTCCCGCCCAGCAGGGGGCTCGGCTGTGCCCTCCTCTGGACGCCACGTTGTCCCAGCCCAGGTTCATGTCAATGGTGGGGCGTTAGCATCTGAGCGGGAGACAGACATCCTGGACGATGAATTGCCAAACCAGGATGGTCACAGTGCGGGCAGCATGGGCACACTCTCTTCTCTGGACGGGGTCACCAACACCAGTGAGGGGGGCTACCCAGAGGCCCTGTCCCCACTGACCAACGGTCTGGACAAGTCCTACCCCATGGAGCCTATGGTCAATGGAGGAGGCTACCCCTACGAGTCTGCCAGCCGGGCGGGGCCTGCCCATGCTGGCCACACGGCCCCCATGCGGCCCTCCTACTCTGCACAGGAGGGTTTAGCTGGCTACCAGAGGGAGGGGCCCCACCCAGCCTGGCCACAGCCAGTGACCACCTCCCACTATGCCCATGACCCCAGCGGTATGTTCCGCTCTCAATCCTTTTCGGAAGCTGAACCCCAGCTGCCCCCAGCTCCGGTCCGAGGGGGAAGCAGCCGGGAGGCTGTGCAAAGGGGACTGAATTCATGGCAGCAGCAGCAGCAGCAGCAGCAGCAGCCTCGCCCACCTCCACGCCAGCAGGAAAGAGCCCACTTGGAGAGTCTTGTAGCCAGCAGGCCCGGACCTCAGCCATTGGCAGAGGGACCCATCCCCAGTCTCCCTGAGTTCCCGCGAGCAGCCTCCCAGCAGGAGATTGAACAGTCCATCGAAACACTCAATATGCTGATGCTGGACCTGGAGCCAGCCTCCGCTGCTGCCCCACTACACAAGTCCCAGAGTGTCCCCGGGGCCTGGCCAGGGGCTGGACCACTCTCCTCCCAGCCCCTCTCTGGATCCTCCCGTCAGTCCCATCCACTGACCCAGTCCAGATCTGGCTATATCCCCAGTGGGCATTCGTTGGGAGGACCTGAGCCAGCCCCACGGGCCTCTCTGGAGTCTGTCCCTCCTGGCAGGTCTTACTCACCTTATGACTATCAGCCATGTTTGGCTGGGCCTAACCAGGATTTCCATTCAAAGAGCCCAGCCTCTTCCTCCTTGCCTGCCTTCCTTCCGACCACCCACGGACCTCCAGGGCCTCAGCAACCCCCAGCCTCTCTCCCTGGCCTCACTGCTCAGCCTCTGCTCGGACCAAAGGAAGCGACTTCAGACCCCTCCCGGGGACCAGAGGAGGAGCCATTGAATTTAGAAGGGCTGGTGGCCCACAGGGTAGCAGGGGTACAGGCTCGGGAGAAGCAGCCTGCAGAGCCCCCAGCCCCTCTGCGGAGGCGGGCGGCCGGAGATGGACAGTATGAGAACCAGTCTCCAGAAGCCACATCCCCTCGTGGACCTGGGGTTCGCGGACCTGTCCAGTGTGTCGGACCGGAGCTGGCTCTTACCATCGCTCTCAATCCTGGAGGGCGGCCCAAAGAGCCCCATTTGCACAGCTACAAGGAGGCCTTCGAGGAGATGGAGGGAACCTCCCCGAGC

TNS1 SG_2

TGGAGGGAACCTCCCCGAGCGGACCACCACCCAGTGGGGTGCGGGGACCCCCGGGTCTGGCCAAGGGACCCCTGTCTGCTCTGGGCCTGAAACCTCACAACCCAGCGGACATCCTGTTGCACCCCACAGGAGAGCCCCGGGGATATGTGGAGTCTGTGGCACGGACAGCGGTGGCTGGACCCCGAGCTCAGGACTCTGAGCCCAAGAGCTTTAGTGCTCCAGCCACCCAGGCCTATGGCCATGAGATACCCCTGAGGAACGGGACCCTGGGTGGCTCCTTTGTCTCCCCCAGCCCCCTCTCCACCAGCAGCCCCATCCTCAGTGCTGACAGCACTTCAGTGGGGAGTTTCCCGTCGGGAGAGAGCAGTGACCAGGGTCCCCGGGGACCCACCCAGCCTCTGTTGGAGTCTGGCTTCCGCTCAGGCGGACTGGGACAGCCCGGACCATCTGCCCAGAGAAACTACCAGAGCTCTTCTCCTCTCCCGACTGTGGGCAGTAGCTACAGCAGCCCCGACTACTCACTTCAGCATTTCAGCTCCTCTCCGGAAAGCCAGGCCCGAGCTCAGTTCAGTGTGGCTGGCGTCCACACGGTGCCTGGGGGACCTCAGGCGCGCCACAGAACAGTGGGCACCAACGGACCCCCTGGACCTGGCTTCGGCCGGCGGGCCATCAATCCCAGCATGGCTGCCCCCAGCAGTCCCAGTTTGAGCCATCACCAGATGATGGGTCCACCAGGCACTGGCTTCCATGGTAGCACTGTCTCCAGCCCCCAGAGCAGTGCAGCGACCACCCCGGGGAGCCCCAGCCTGTGTCGGCACCCAGCAGGGGTCTACCAGGTTTCTGGCCTCCACAACAAAGTGGCCACCGGACCGGGGGGACCCAGCCTGGGCCGGCACCCTGGGGCTCACCAAGGCAACCTGGCCTCCGGTCTTCATAGCAATGCAATAGCCGGACCTGGAGGACCCAGCCTGGGCCGTCACCTCGGAGGGTCTGGATCTGTGGTTCCCGGCGGACCCTGCTTGGACCGGCATGTGGCCTATGGCGGCTATTCTGGACCGGAGGATCGGAGACCCACACTGTCCCGGCAGAGCAGTGCCTCTGGCTACCAGGCTCCTTCCACGCCCTCCTTCCCTGTCTCCCCTGCCTACTACCCTGGCCTGAGCAGCCCTGCCACCTCCCCGTCACCAGACTCCGCAGCCTTCCGGCAAGGGGGACCAACACCAGCCTTGCCAGAGAAGCGAAGGATGGGAGTGGGAGACCGGGCAGGCAGCCTCCCCAACTATGCCACCATCAATGGGAAGGTGTCTGGACCTGTCGCCAGCGGCATGTCCGGACCCAGCGGGGGCAGCACCGTCTCCTTCTCCCACACTCTGCCCGACTTCTCCAAGTACTCCATGCCAGACAACGGACCGGAGACGCGGGCTAAAGTGAAGTTTGTCCAGGACACTTCTAAGTATTGGTACAAGCCTGAGATCTCCAGGGAGCAGGCCATCGCGCTCCTCAAGGACCAGGAGCCGGGGGCCTTCATCATCCGCGACAGTCACTCCTTCCGAGGCGCGTACGGGCTGGCCATGAAGGTGTCTGGACCACCTCCAACCATCATGCAGCAGAATAAAAAAGGAGACATGACCCATGAGCTGGTCAGGCATTTTCTGATAGAGACTGGCCCCAGAGGAGTCAAGCTCAAGGGCTGCCCCAATGAGCCAAACTTCGGATCGCTGTCTGCCCTGGTCTACCAGCACTCCATCATCCCATTGGCCCTGCCTTGCAAGCTGGTCATTCCAAACCGAGACCCCACAGATGAATCGAAAGATAGCTCCGGCCCTGCCAACTCAACTGCAGACCTGCTGAAACAAGGGGCAGCCTGCAATGTGCTCTTCATCAACTCTGTGGACATGGAGTCACTCACTGGGCCACAGGCCATCTCTAAAGCCACATCTGAGACGTTGGCTGCAGACCCCACACCAGCTGCCACCATCGTTCACTTCAAAGTCTCTGCCCAGGGAATCACTCTGACTGACAACCAGAGAAAGCTCTTTTTCAGACGCCACTACCCTCTCAACACTGTCACCTTCTGTGACCTGGATCCACAGGAAAGAAAGTGGATGAAAACAGAGGGTGGTGCCCCTGCTAAGCTCTTCGGCTTCGTGGCCCGGAAGCAGGGCAGCACCACGGACAACGCCTGCCACCTCTTTGCTGAGCTTGACCCCAACCAGCCGGCCTCTGCCATCGTCAACTTCGTCTCCAAGGTCATGCTGAATGCCGGCCAAAAGAGATGAGTCGACGGTACCGCGGGCCCGGGAT

mGL-TNS1 HDR template sequence

TACATGAGTGTGAGCCGGACCATGGCCCCTCCCTTCCTCACCCAGTGCCCAGTGGAGAGGATGGCCAGCCCAGGGGCCTCCCCACCTCTCCGGGAAACGCATGGTGCTGGCGGCTGCCCGCCACTGCAGCAACAAGGGCTCTGTGCCCTCTTGTTTTCTGGGCCCTTAGAAATCAGGGAGGCTGCTGCCAAGAAGCAGCTCTGGCAGGCAGTGGGAGCTGCCAGAGGTGGGGAGAAGGCCAAGGAACAAAGGGCAGGAGTTGGCCTGCCTCCGGGAGGGCTGGAATGTGTGCTGTGGTCTGGCTTACCTGGAACCCAGGTGAACGTGTCACCTGAGATGCCCCCTCCCCTGAGAGGTTTCCCGCCCACTCCACAGCTCGAGAGCTAGGGGCGGGCAGTGGGAAGTCTCCGTTCCAGGGCTGGAGAGCGCCCAGCGCAGGCTCTCCTTCCTCTTCCAGAGCAGTCTGGGGCCTTTCGGGAGACTTCTGCCTGGCCAGGCAGCCAGTGTTGGGAGCCAGAGCTCCTGGGTTCCTGGGCCAGTCTGGCCCTGGCTCTGAGCTCTGGAGTCACAGGTTGAAGGCAGTGGCCAAAGGACTGATGGGAACCAAGAATTGGTGTCATCACAGTTTGGTTCTGAAATGGCCTGAGGTCCTTGCCAGCCCTACCCAGCTTGTTCTCGCCGGCACTCCCAAGCCGGCCACATTGGCCTTGCCAGAGGAAGCCTGTGGCCTTGGCCTTGGGTTGAAGAGCAGCCAAGCACCCTGGGCTGCATATGGGGGTCACACAGGGTGTGTATGGGGTGCAGCTATCTGGGGCCTTCCCTGTGGGCTGGATTCTGATATGGACCCTCCGCTGCCTGCTGTCTCCCTAGAAACATGGTGAGCAAGGGCGAGGAGCTGTTCACCGGGGTGGTGCCCATCCTGGTCGAGCTGGACGGCGACGTAAACGGCCACAAGTTCAGCGTCCGCGGCGAGGGCGAGGGCGATGCCACCAACGGCAAGCTGACCCTGAAGTTCATCTGCACCACCGGCAAGCTGCCCGTGCCCTGGCCCACCCTCGTGACCACCTTAGGCTACGGCGTGGCCTGCTTCGCCCGCTACCCCGACCACATGAAGCAGCACGACTTCTTCAAGTCCGCCATGCCCGAAGGCTACGTCCAGGAGCGCACCATCTCTTTCAAGGACGACGGTACCTACAAGACCCGCGCCGAGGTGAAGTTCGAGGGCGACACCCTGGTGAACCGCATCGTGCTGAAGGGCATCGACTTCAAGGAGGACGGCAACATCCTGGGGCACAAGCTGGAGTACAACTTCAACAGCCACAAGGTCTATATCACGGCCGACAAGCAGAAGAACGGCATCAAGGCTAACTTCAAGACCCGCCACAACGTTGAGGACGGCGGCGTGCAGCTCGCCGACCACTACCAGCAGAACACCCCCATCGGCGACGGCCCCGTGCTGCTGCCCGACAACCACTACCTGAGCCATCAGTCCAAACTGAGCAAAGACCCCAACGAGAAGCGCGATCACATGGTCCTGAAGGAGAGGGTGACCGCCGCCGGGATTACACATGACATGGACGAGCTGTACAAGGGAGGTGGTAGTGGTGGAGGAAGTGGTGGAGGTATGTCTGTATCTAGAACTATGGAGGACAGCTGTGAGCTGGACCTGGTGTACGTCACAGAGAGGATCATCGCTGTCTCCTTCCCCAGCACAGCCAATGAGGAGAACTTCCGGAGCAACCTCCGTGAGGTGGCGCAGATGCTCAAGTCCAAACATGGAGGCAACTACCTGGTGAGGATGGATCCTCGTGCCCACTGTCCTGTGCCCTCTATGCTTCCATCTGCTCACCACCTCTACATGACTGGTATCCCTGCCACCAGCCCCTGTGCCAGCATGCCCTCTTCTCTTTGTTTATCTGCCTTTCCCCTCAGGCAGCTCTCGTGCCTGAGTCTTCAATCACTCTCCAGCCCAGCTCTCCTCTAAGGAGTGTGCTGCAGCTGGAATGTAGCATCTGGAACTCTGTGTGTGCATGTGTGTGCTGGCCAGGAGAAGAGAGTACCAATAAGAGTTCAGGTTATTGAGCGCTTGCTACTAGCCAGGCCCTGTTCCAACTGCTTTCCACGCACTTAACCTTTGCAGCAGCCCTAGCAAGCAGGCACTTGCCAGAGCACAGAGAGGTGGGGTTATTTGCCTAAGGTTGCAGGGTCAGGAAGTGGCAGAGCTGGCACAGGGTCGGTGCCTCTGAGAAGTCTGAGGGCAGAGTCTTGGCAACATGTGTCTTAAGGGACCCTGGGTTGCTGGCACCTGGCTCGGCCTGGCCCAGAGGAGTCACCTTGGCACACGGGAGAGAGGTGCTGGCGGTGGTCTCCACTAACGCAGAAAGGCTGATCCGTCCTTGGCCCCAGAGGAGGGCTCAGGTCCATTTTAGAGGGAGGTGCCTGTGGACTGGACCCACAGATCTGATCTGTACAACCTCAGGTGGCTGTCCCCACATGAGTGTGAGCCGGACCATGGAT

IDR132_1

aggctgtgcaaaggggactgaattcatggcagcagcagcagcagcagcagcagcagcctcgcccacctccacgccagcaggaaagagcccacttggagagtcttgtagccagcaggcccagccctcagccattggcagagacccccatccccagtctccctgagttcccgcgagcagcctcccagcaggagattgaacagtccatcgaacccatcctcagtgctgacagcacttcagtggggagtttcccgtcgggagagagcagtgaccagggtccccggacgcccacccagcctctgttggagtctggcttccgctcaggcagcctgggacagcccagcccatctgcccagagaaactaccagagctcttctcctctcccgactgtgggcagtagctacagcagccccgactactcacttcagcatttcagctcctctccggaaagccaggcccgagctcagttcagtgtggctggcgtccacacggtgcctgggagccctcaggcgcgccacagaacagtgggcaccaacactccccctagtcctggcttcggccggcgggccatcaatcccagcatggctgcccccagcagtcccagtttgagccatcaccagatgatgggtccaccaggcactggcttccatggtagcactgtctccagcccccagagcagtgcagcgaccaccccggggagccccagcctgtgtcggcacccagcaggggtctaccaggtttctggcctccacaacaaagtggccaccaccccggggagtcccagcctgggccggcaccctggggctcaccaaggcaacctggcctccggtcttcatagcaatgcaatagccagccctggaagccccagcctgggccgtcacctcggagggtctggatctgtggttcccggcagcccctgcttggaccggcatgtggcctatggcggctattctaccccggaggatcggagacccacactgtcccggcagagcagtgcctctggctaccaggctccttccacgccctccttccctgtctcccctgcctactaccctggcctgagcagccctgccacctccccgtcaccagactccgcagccttccggcaagggagcccaacaccagccttgccagagaagcgaaggatgtcagtgggagaccgggcaggcagcctccccaactatgccaccatcaatgggaaggtgtcttcgcctgtcgccagcggcatgtccagtcccagcgggggcagcaccgtctccttctcccacactctgcccgacttctccaagtactccatgccagacaacagcccggagacgcggacactcaatatgctgatgct

IDR132_2

Acactcaatatgctgatgctggacctggagccagcctccgctgctgccccactacacaagtcccagagtgtccccggggcctggccaggggcttctccactctcctcccagcccctctctggatcctcccgtcagtcccatccactgacccagtccagatctggctatatccccagtgggcattcgttgggaacccctgagccagccccacgggcctctctggagtctgtccctcctggcaggtcttactcaccttatgactatcagccatgtttggctgggcctaaccaggatttccattcaaagagcccagcctcttcctccttgcctgccttccttccgaccacccacagccctccagggcctcagcaacccccagcctctctccctggcctcactgctcagcctctgctctcaccaaaggaagcgacttcagacccctcccggactccagaggaggagccattgaatttagaagggctggtggcccacagggtagcaggggtacaggctcgggagaagcagcctgcagagcccccagcccctctgcggaggcgggcggccagtgatggacagtatgagaaccagtctccagaagccacatcccctcgtagccctggggttcgctcccctgtccagtgtgtctccccggagctggctcttaccatcgctctcaatcctggagggcggcccaaagagccccatttgcacagctacaaggaggccttcgaggagatggagggaacctccccgagcagcccaccacccagtggggtgcggtcccccccgggtctggccaagacacccctgtctgctctgggcctgaaacctcacaacccagcggacatcctgttgcaccccacaggagagccccggagctatgtggagtctgtggcacggacagcggtggctggaccccgagctcaggactctgagcccaagagctttagtgctccagccacccaggcctatggccatgagatacccctgaggaacgggaccctgggtggctcctttgtctcccccagccccctctccaccagcagcgctaaagtgaagtttgtcca

IDR2scr_1

aggctgtgcaaaggggactgaattcatggcagcagcagcagcagcagcagcagcagcctcgcccacctccacgccagcaggaaagagcccacttggagagtcttgtagccagcaggcccagccctcagccattggcagagacccccatccccagtctccctgagttcccgcgagcagcctcccagcaggagattgaacagtccatcgaacagttcaagggctacgaggcctctagccagtccgtgcaactggaaagcctgacccagggcgcctgtgaccccgacccttttgccgagcggccttctatgctgggcggaagcggaggcctgggaagcaacaacgtggcccccccaccagcccctcaggccgacaccccttccagcgccctggaacctgagggtacaacccctagacgggcccagagaagcgtgccaagtccccacggcccgctgctgtgccctgccacacccagaaccgccctgcctgctagccctggcctcgccaaggcctccgccctgcagcctctgtatagacctcatgtgcagctgagccccagccacaagcacaacgtgccctcttctgcccggagagtcgccgagggaagccaaagcgtgcagtggggccacagctcccacagaatccccagccctagctacatcggcaacagcgctgagggccctccccacaatgcccctttctctgatcagcggggcctggcctacgagcctctgttcagcgttcctctgtacatcgccctgaagagccctacagagagcggcgagcctgctcgccacagcgacgccgaaaccagcagcgtgcctcaggtggagggactgtacagcagcccaagcgagcacccccccgagagacccgctctgctgacacctccaaaagccgcttctagacctagccctagcccaagcagcaccgccagactgcagaacggcgctctgcccgtgctgctgcaaatggaacctgctgatctgcctcactacaccgacggctctcagcagggcggcagcctgcacaaagccaggggccctgccagcttctacggcccttctacacctgccgagcgggaatctgcaaccagcgccagagtgtccaagacccctctgggccccagcacacctctggaaaagctgctgcttctgcctcctatgctgccaccaaccgaggaaggcgtgccccccgcctccgagctgagcggctccctcccgaagatccctcctgaagccccttctaataccagatcc

IDR2scr_2

CcccttctaataccagatcccccatcctcagtgctgacagcacttcagtggggagtttcccgtcgggagagagcagtgaccagggtccccggacgcccacccagcctctgttggagtctggcttccgctcaggcagcctgggacagcccagcccatctgcccagagaaactaccagagctcttctcctctcccgactgtgggcagtagctacagcagccccgactactcacttcagcatttcagctcctctccggaaagccaggcccgagctcagttcagtgtggctggcgtccacacggtgcctgggagccctcaggcgcgccacagaacagtgggcaccaacactccccctagtcctggcttcggccggcgggccatcaatcccagcatggctgcccccagcagtcccagtttgagccatcaccagatgatgggtccaccaggcactggcttccatggtagcactgtctccagcccccagagcagtgcagcgaccaccccggggagccccagcctgtgtcggcacccagcaggggtctaccaggtttctggcctccacaacaaagtggccaccaccccggggagtcccagcctgggccggcaccctggggctcaccaaggcaacctggcctccggtcttcatagcaatgcaatagccagccctggaagccccagcctgggccgtcacctcggagggtctggatctgtggttcccggcagcccctgcttggaccggcatgtggcctatggcggctattctaccccggaggatcggagacccacactgtcccggcagagcagtgcctctggctaccaggctccttccacgccctccttccctgtctcccctgcctactaccctggcctgagcagccctgccacctccccgtcaccagactccgcagccttccggcaagggagcccaacaccagccttgccagagaagcgaaggatgtcagtgggagaccgggcaggcagcctccccaactatgccaccatcaatgggaaggtgtcttcgcctgtcgccagcggcatgtccagtcccagcgggggcagcaccgtctccttctcccacactctgcccgacttctccaagtactccatgccagacaacagcccggagacgcggGctaaagtgaagtttgtcca

IDR3scr_1

aggctgtgcaaaggggactgaattcatggcagcagcagcagcagcagcagcagcagcctcgcccacctccacgccagcaggaaagagcccacttggagagtcttgtagccagcaggcccagccctcagccattggcagagacccccatccccagtctccctgagttcccgcgagcagcctcccagcaggagattgaacagtccatcgaaAcactcaatatgctgatgctggacctggagccagcctccgctgctgccccactacacaagtcccagagtgtccccggggcctggccaggggcttctccactctcctcccagcccctctctggatcctcccgtcagtcccatccactgacccagtccagatctggctatatccccagtgggcattcgttgggaacccctgagccagccccacgggcctctctggagtctgtccctcctggcaggtcttactcaccttatgactatcagccatgtttggctgggcctaaccaggatttccattcaaagagcccagcctcttcctccttgcctgccttccttccgaccacccacagccctccagggcctcagcaacccccagcctctctccctggcctcactgctcagcctctgctctcaccaaaggaagcgacttcagacccctcccggactccagaggaggagccattgaatttagaagggctggtggcccacagggtagcaggggtacaggctcgggagaagcagcctgcagagcccccagcccctctgcggaggcgggcggccagtgatggacagtatgagaaccagtctccagaagccacatcccctcgtagccctggggttcgctcccctgtccagtgtgtctccccggagctggctcttaccatcgctctcaatcctggagggcggcccaaagagccccatttgcacagctacaaggaggccttcgaggagatggagggaacctccccgagcagcccaccacccagtggggtgcggtcccccccgggtctggccaagacacccctgtctgctctgggcctgaaacctcacaacccagcggacatcctgttgcaccccacaggagagccccggagctatgtggagtctgtggcacggacagcggtggctggaccccgagctcaggactctgagcccaagagctttagtgctccagccacccaggcctatggccatgagatacccctgaggaacgggaccctgggtggctcctttgtctcccccagccccctctccaccagcagc

IDR3scr_2

GccccctctccaccagcagcagcagacagcctctggccttcagcagcagcacccctcctccattcggagatgattctgctgttgtgaactcctacttcgcggtgtcatcttaccacggccctggatctgctggtaccgccggcatgtccccaggcaacgcccctgaaggctttagccctgataacacctccggcagttttagcacctacatgaccacaggaagacggcaccaggcctgcagcctgcggcaatctggcagaacccctgccatgttcgccatccctgagcacgtgccccacagcttcagccctaacgacagcctggctagcgtgtcgagcggcatcagaagcgtgggcaattacctgaagcacaccggcagcggcctgctcttcggcgcctacgagcccctgctgagacctggcctgagcagcggcctgtaccctggctctcagcactctgtgcagcccaccgccggacctgggcatagacctgtgcctagcggctccggcacggaagtgtcccccggtacataccccagcccaagatctccaagccctaagatgcctgcctctctggccgagggcacaagcgacgccagacccgcccctccccctggcagccctcatagattcgctaatacccctagcctgagcctgctgctgttcagaagccagagcagcgcccctaggctggccagcgccggcatccccgtggacgcccacgcccctcctggcagtggaacatctctgagcagcgctacacacagctctgtctctgtgtccggaagcgcaccaagctatagcagcgcccagggcgagcctaaaaccgacctgctgagcgacatgagcagccccgtctacggcaacagcgccagccccgacggcagccagacctccggcggaggcaccggcagccaatctacaggccaccggcagcctagctcaagaagcatccggcggacctcttcctccccaccacctagagtgacacagagccccaccgtgacctctcagggccagcgcccacctggcagctactacagccagcacaacagccccaagggcgtgggagcccggcacccccaccagcctctgtcaccctcccccgccatgcaggtgagctgtgtgaacGctaaagtgaagtttgtcca

IDR23scr_1

aggctgtgcaaaggggactgaattcatggcagcagcagcagcagcagcagcagcagcctcgcccacctccacgccagcaggaaagagcccacttggagagtcttgtagccagcaggcccagccctcagccattggcagagacccccatccccagtctccctgagttcccgcgagcagcctcccagcaggagattgaacagtccatcgaacagttcaagggctacgaggcctctagccagtccgtgcaactggaaagcctgacccagggcgcctgtgaccccgacccttttgccgagcggccttctatgctgggcggaagcggaggcctgggaagcaacaacgtggcccccccaccagcccctcaggccgacaccccttccagcgccctggaacctgagggtacaacccctagacgggcccagagaagcgtgccaagtccccacggcccgctgctgtgccctgccacacccagaaccgccctgcctgctagccctggcctcgccaaggcctccgccctgcagcctctgtatagacctcatgtgcagctgagccccagccacaagcacaacgtgccctcttctgcccggagagtcgccgagggaagccaaagcgtgcagtggggccacagctcccacagaatccccagccctagctacatcggcaacagcgctgagggccctccccacaatgcccctttctctgatcagcggggcctggcctacgagcctctgttcagcgttcctctgtacatcgccctgaagagccctacagagagcggcgagcctgctcgccacagcgacgccgaaaccagcagcgtgcctcaggtggagggactgtacagcagcccaagcgagcacccccccgagagacccgctctgctgacacctccaaaagccgcttctagacctagccctagcccaagcagcaccgccagactgcagaacggcgctctgcccgtgctgctgcaaatggaacctgctgatctgcctcactacaccgacggctctcagcagggcggcagcctgcacaaagccaggggccctgccagcttctacggcccttctacacctgccgagcgggaatctgcaaccagcgccagagtgtccaagacccctctgggccccagcacacctctggaaaagctgctgcttctgcctcctatgctgccaccaaccgaggaaggcgtgccccccgcctccgagctgagcggctccctcccgaagatccctcctgaagccccttctaataccagatcc

IDR23scr_2

ccccttctaataccagatccagcagacagcctctggccttcagcagcagcacccctcctccattcggagatgattctgctgttgtgaactcctacttcgcggtgtcatcttaccacggccctggatctgctggtaccgccggcatgtccccaggcaacgcccctgaaggctttagccctgataacacctccggcagttttagcacctacatgaccacaggaagacggcaccaggcctgcagcctgcggcaatctggcagaacccctgccatgttcgccatccctgagcacgtgccccacagcttcagccctaacgacagcctggctagcgtgtcgagcggcatcagaagcgtgggcaattacctgaagcacaccggcagcggcctgctcttcggcgcctacgagcccctgctgagacctggcctgagcagcggcctgtaccctggctctcagcactctgtgcagcccaccgccggacctgggcatagacctgtgcctagcggctccggcacggaagtgtcccccggtacataccccagcccaagatctccaagccctaagatgcctgcctctctggccgagggcacaagcgacgccagacccgcccctccccctggcagccctcatagattcgctaatacccctagcctgagcctgctgctgttcagaagccagagcagcgcccctaggctggccagcgccggcatccccgtggacgcccacgcccctcctggcagtggaacatctctgagcagcgctacacacagctctgtctctgtgtccggaagcgcaccaagctatagcagcgcccagggcgagcctaaaaccgacctgctgagcgacatgagcagccccgtctacggcaacagcgccagccccgacggcagccagacctccggcggaggcaccggcagccaatctacaggccaccggcagcctagctcaagaagcatccggcggacctcttcctccccaccacctagagtgacacagagccccaccgtgacctctcagggccagcgcccacctggcagctactacagccagcacaacagccccaagggcgtgggagcccggcacccccaccagcctctgtcaccctcccccgccatgcaggtgagctgtgtgaacGctaaagtgaagtttgtcca

TNS1_C-term

Gctaaagtgaagtttgtccaggacacttctaagtattggtacaagcctgagatctccagggagcaggccatcgcgctcctcaaggaccaggagccgggggccttcatcatccgcgacagtcactccttccgaggcgcgtacgggctggccatgaaggtgtcttcgccacctccaaccatcatgcagcagaataaaaaaggagacatgacccatgagctggtcaggcattttctgatagagactggccccagaggagtcaagctcaagggctgccccaatgagccaaacttcggatcgctgtctgccctggtctaccagcactccatcatcccattggccctgccttgcaagctggtcattccaaaccgagaccccacagatgaatcgaaagatagctccggccctgccaactcaactgcagacctgctgaaacaaggggcagcctgcaatgtgctcttcatcaactctgtggacatggagtcactcactgggccacaggccatctctaaagccacatctgagacgttggctgcagaccccacaccagctgccaccatcgttcacttcaaagtctctgcccagggaatcactctgactgacaaccagagaaagctctttttcagacgccactaccctctcaacactgtcaccttctgtgacctggatccacaggaaagaaagtggatgaaaacagagggtggtgcccctgctaagctcttcggcttcgtggcccggaagcagggcagcaccacggacaacgcctgccacctctttgctgagcttgaccccaaccagccggcctctgccatcgtcaacttcgtctccaaggtcatgctgaatgccggccaaaagagatgagtcgacggtaccgcgggcccgggat

**Translation of IDR2scr and IDR3scr sequences**

IDR2 WT

TLNMLMLDLEPASAAAPLHKSQSVPGAWPGASPLSSQPLSGSSRQSHPLTQSRSGYIPSGHSLGTPEPAPRASLESVPPGRSYSPYDYQPCLAGPNQDFHSKSPASSSLPAFLPTTHSPPGPQQPPASLPGLTAQPLLSPKEATSDPSRTPEEEPLNLEGLVAHRVAGVQAREKQPAEPPAPLRRRAASDGQYENQSPEATSPRSPGVRSPVQCVSPELALTIALNPGGRPKEPHLHSYKEAFEEMEGTSPSSPPPSGVRSPPGLAKTPLSALGLKPHNPADILLHPTGEPRSYVESVARTAVAGPRAQDSEPKSFSAPATQAYGHEIPLRNGTLGGSFVSPSPLSTSS

IDR2scr

QFKGYEASSQSVQLESLTQGACDPDPFAERPSMLGGSGGLGSNNVAPPPAPQADTPSSALEPEGTTPRRAQRSVPSPHGPLLCPATPRTALPASPGLAKASALQPLYRPHVQLSPSHKHNVPSSARRVAEGSQSVQWGHSSHRIPSPSYIGNSAEGPPHNAPFSDQRGLAYEPLFSVPLYIALKSPTESGEPARHSDAETSSVPQVEGLYSSPSEHPPERPALLTPPKAASRPSPSPSSTARLQNGALPVLLQMEPADLPHYTDGSQQGGSLHKARGPASFYGPSTPAERESATSARVSKTPLGPSTPLEKLLLLPPMLPPTEEGVPPASELSGSLPKIPPEAPSNTRS

IDR3 WT

PILSADSTSVGSFPSGESSDQGPRTPTQPLLESGFRSGSLGQPSPSAQRNYQSSSPLPTVGSSYSSPDYSLQHFSSSPESQARAQFSVAGVHTVPGSPQARHRTVGTNTPPSPGFGRRAINPSMAAPSSPSLSHHQMMGPPGTGFHGSTVSSPQSSAATTPGSPSLCRHPAGVYQVSGLHNKVATTPGSPSLGRHPGAHQGNLASGLHSNAIASPGSPSLGRHLGGSGSVVPGSPCLDRHVAYGGYSTPEDRRPTLSRQSSASGYQAPSTPSFPVSPAYYPGLSSPATSPSPDSAAFRQGSPTPALPEKRRMSVGDRAGSLPNYATINGKVSSPVASGMSSPSGGSTVSFSHTLPDFSKYSMPDNSPETR

IDR3scr

SRQPLAFSSSTPPPFGDDSAVVNSYFAVSSYHGPGSAGTAGMSPGNAPEGFSPDNTSGSFSTYMTTGRRHQACSLRQSGRTPAMFAIPEHVPHSFSPNDSLASVSSGIRSVGNYLKHTGSGLLFGAYEPLLRPGLSSGLYPGSQHSVQPTAGPGHRPVPSGSGTEVSPGTYPSPRSPSPKMPASLAEGTSDARPAPPPGSPHRFANTPSLSLLLFRSQSSAPRLASAGIPVDAHAPPGSGTSLSSATHSSVSVSGSAPSYSSAQGEPKTDLLSDMSSPVYGNSASPDGSQTSGGGTGSQSTGHRQPSSRSIRRTSSSPPPRVTQSPTVTSQGQRPPGSYYSQHNSPKGVGARHPHQPLSPSPAMQVSCVN

| **Primer** | **Sequence** |
| --- | --- |
| TNS1 dPTP F | TCAGATCTCGAGCTCAAGCTTACATGAACAACAAGCCCTTGTTTCT |
| TNS1 dPTP R | AGAAACAAGGGCTTGTTGTTCATGTAAGCTTGAGCTCGAGATCTGA |
| TNS1 dC2 F | GCTCTCCGGCTCCATCAAACCAGAGAAAATTCAAGGCATGGAG |
| TNS1 dC2 R | CTCCATGCCTTGAATTTTCTCTGGTTTGATGGAGCCGGAGAGC |
| TNS1 dIDR F | GCTGGGACTCCTACGACGCTAAAGTGAAGTTTGTCCAGGAC |
| TNS1 dIDR R | GTCCTGGACAAACTTCACTTTAGCGTCGTAGGAGTCCCAGC |
| TNS1 dIDR1 F | GCTGGGACTCCTACGACACACTCAATATGCTGATGCTGG |
| TNS1 dIDR1 R | CCAGCATCAGCATATTGAGTGTGTCGTAGGAGTCCCAGC |
| TNS1 dIDR2 F | CAGGAGATTGAACAGTCCATCGAACCCATCCTCAGTGCTGAC |
| TNS1 dIDR2 R | GTCAGCACTGAGGATGGGTTCGATGGACTGTTCAATCTCCTG |
| TNS1 dIDR3 F | CCCTCTCCACCAGCAGCGCTAAAGTGAAGTTTGTCCAGGACACT |
| TNS1 dIDR3 R | AGTGTCCTGGACAAACTTCACTTTAGCGCTGCTGGTGGAGAGGG |
| TNS1 dSH2 F | GTTTGTCCAGGACACTTCTAAGTATCGAGACCCCACAGATGAATC |
| TNS1 dSH2 R | GATTCATCTGTGGGGTCTCGATACTTAGAAGTGTCCTGGACAAAC |
| TNS1 dPTB F | CAGACCTGCTGAAACAAGGGTGAGTCGACGGTACCGC |
| TNS1 dPTB R | GCGGTACCGTCGACTCACCCTTGTTTCAGCAGGTCTG |
| TNS1 HiFi F | ggaggagaatcccggcccttCTATGAGTGTGAGCCGGAC |
| TNS1 HiFi R | agatgagtttttgttccattGATCTCTTTTGGCCGGCATTC |
