# Supplementary material for: Adhesion-derived condensates control component availability to regulate adhesion dynamics
Source: Nat Commun. 2026 Jun 5;17:7222. doi: 10.1038/s41467-026-74001-3 (PMC13396368; doi:10.1038/s41467-026-74001-3)
Supplement: Supplementary file 15 — Reporting Summary [file 41467_2026_74001_MOESM15_ESM.pdf]

nature portfolio

Corresponding author(s): Johanna Ivaska, Michal Dibus

Last updated by author(s): May 6, 2026

Reporting Summary

Nature Portfolio wishes to improve the reproducibility of the work that we publish. This form provides structure for consistency and transparency in reporting. For further information on Nature Portfolio policies, see our Editorial Policies and the Editorial Policy Checklist.

Statistics

For all statistical analyses, confirm that the following items are present in the figure legend, table legend, main text, or Methods section.

n/a	Confirmed	
☐	☒	The exact sample size (*n*) for each experimental group/condition, given as a discrete number and unit of measurement
☐	☒	A statement on whether measurements were taken from distinct samples or whether the same sample was measured repeatedly
☐	☒	The statistical test(s) used AND whether they are one- or two-sided<br>*Only common tests should be described solely by name; describe more complex techniques in the Methods section.*
☒	☐	A description of all covariates tested
☐	☒	A description of any assumptions or corrections, such as tests of normality and adjustment for multiple comparisons
☐	☒	A full description of the statistical parameters including central tendency (e.g. means) or other basic estimates (e.g. regression coefficient) AND variation (e.g. standard deviation) or associated estimates of uncertainty (e.g. confidence intervals)
☐	☒	For null hypothesis testing, the test statistic (e.g. *F*, *t*, *r*) with confidence intervals, effect sizes, degrees of freedom and *P* value noted<br>*Give P values as exact values whenever suitable.*
☒	☐	For Bayesian analysis, information on the choice of priors and Markov chain Monte Carlo settings
☒	☐	For hierarchical and complex designs, identification of the appropriate level for tests and full reporting of outcomes
☐	☒	Estimates of effect sizes (e.g. Cohen's *d*, Pearson's *r*), indicating how they were calculated

Our web collection on statistics for biologists contains articles on many of the points above.

Software and code

Policy information about availability of computer code

Data collection	Data was collected as indicated in the Methods section. All instruments used were commercially available and controlled using software provided by the manufacturer, unless otherwise stated.
Data analysis	Data analyses were performed as described in the Methods.

For manuscripts utilizing custom algorithms or software that are central to the research but not yet described in published literature, software must be made available to editors and reviewers. We strongly encourage code deposition in a community repository (e.g. GitHub). See the Nature Portfolio guidelines for submitting code & software for further information.

Data

Policy information about availability of data

All manuscripts must include a data availability statement. This statement should provide the following information, where applicable:

- Accession codes, unique identifiers, or web links for publicly available datasets
- A description of any restrictions on data availability
- For clinical datasets or third party data, please ensure that the statement adheres to our policy

Data supporting the findings of this study are available within the paper and its source data supplementary information files. Statistical source data and uncropped and unprocessed blots are provided for all figures. Proteomic datasets have been deposited to ProteomeXchange Consortium via the PRIDE partner repository (PXD069837, PXD069806) and to Zenodo (10.5281/zenodo.17426146, 10.5281/zenodo.17408117). Source data are provided with this paper (Source Data).

Research involving human participants, their data, or biological material

Policy information about studies with human participants or human data. See also policy information about sex, gender (identity/presentation), and sexual orientation and race, ethnicity and racism.

Reporting on sex and gender	N/A
Reporting on race, ethnicity, or other socially relevant groupings	N/A
Population characteristics	N/A
Recruitment	N/A
Ethics oversight	N/A

Note that full information on the approval of the study protocol must also be provided in the manuscript.

Field-specific reporting

Please select the one below that is the best fit for your research. If you are not sure, read the appropriate sections before making your selection.

☒ Life sciences ☐ Behavioural & social sciences ☐ Ecological, evolutionary & environmental sciences

For a reference copy of the document with all sections, see nature.com/documents/nr-reporting-summary-flat.pdf

Life sciences study design

All studies must disclose on these points even when the disclosure is negative.

Sample size	Experiments were performed at least three times, unless otherwise stated. For imaging data, representative images are shown. For those experiments with < 3 independent replicates, sufficient sample sizes (cells, measurements, etc) were used to ensure results are scientifically relevant.
Data exclusions	All the datasets have been tested for outliers - outliers were identified by the ROUT method (Q = 0.2%) and excluded from subsequent analyses.
Replication	To ensure reproducibility, experiments were replicated at least three times (unless otherwise indicated in the figure legends). Key experiments were performed at different times and with different batches of cells, for example.
Randomization	The experiments were not randomized. For randomization of imaging (immunofluorescence), images were taken of multiple fields (or individual cells/spheroids) from the same sample at different locations.
Blinding	Blinding was not considered necessary for experiments where samples needed to be clearly and correctly labelled in order to identify specific samples and experimental conditions (e.g. cell treatments). Analysis software/statistical packages were used as detailed in the methods for robust data analysis, removing user bias. In addition, appropriate controls were included in experiments and control versus treated samples were analysed in the same fashion.

Reporting for specific materials, systems and methods

We require information from authors about some types of materials, experimental systems and methods used in many studies. Here, indicate whether each material, system or method listed is relevant to your study. If you are not sure if a list item applies to your research, read the appropriate section before selecting a response.

Materials & experimental systems

n/a	Involved in the study	
☐	☒	Antibodies
☐	☒	Eukaryotic cell lines
☒	☐	Palaeontology and archaeology
☒	☐	Animals and other organisms
☒	☐	Clinical data
☒	☐	Dual use research of concern
☒	☐	Plants

Methods

n/a	Involved in the study	
☒	☐	ChIP-seq
☒	☐	Flow cytometry
☒	☐	MRI-based neuroimaging

Antibodies

Antibodies used	All antibodies used in the study are indicated in the Methods section.<br><br>Primary antibodies used for Western blotting were as follows: anti-GFP (1:5000, Abcam, ab290); GAPDH (1:4000, HyTest, 5G4 MAb 6C5); anti-TNS1 (1:1000, Sigma, SAB4200283); anti-ZYX (1:1000, Abcam, ab109316); anti-PXN (1:1000, GeneTex, GTX125891); anti-TLN1 (1:1000, Novus Biologicals, NBP2-50320); anti-VCL (1:1000, Sigma, V9131); anti-Myc-Tag (1:1000, CST, 2276S); anti-ITGB1 (1:1000, BD Transduction Laboratories, 610468) ; anti-ITGA5 (1:1000, Invitrogen, PA5-82027); anti-pSer/Thr (1:1000, BD Transduction Laboratories, 612549); anti-p38 (1:1000, CST, 9212S); anti-p-p38 (1:1000, CST, 9216S); anti-AKT (1:1000, CST, 2920S); anti-p-AKT (1:1000, CST, 9275S); anti-ERK (1:1000, CST, 4696S); anti-p-ERK (1:1000, CST, 4370S).<br><br>The antibodies used for IF were as follows: anti-TNS1 (1:100, Sigma, HPA036089); anti-FAK (1:100, BD Transduction Laboratories, 610088); anti-p130CAS (1:100, Cell Signalling Technologies (CST), 13846S); anti-ZYX (1:100, Abcam, ab109316); anti-KANK2 (1:100, Sigma, HPA015643); anti-TLN1 (1:100, Novus Biologicals, NBP2-50320); anti-TLN2 (1:100, BioRad, MCA4771GA); anti-VCL (1:100, Sigma, V9131); anti-PXN (1:200, GeneTex, GTX125891); anti-DCP1B (1:200, CST, 13233S); anti-DDX6 (1:200, Bethyl Laboratories, A300-460A); anti-EDC3 (1:100, Abcam, ab168851); anti-pY (1:100, BD Transduction Laboratories, 610000); anti-pCAS Y410 (1:100, CST, 4011S); anti-pFAK Y397 (1:100, CST, 8556S); anti-pPXN Y118 (1:100, CST, 2541S); anti-pZYX S142/143 (1:100, CST, 4863S); anti-active ITGB1 clone 12G10 (1:25, isolated from hybridoma cells, AB_928074); anti-Myc-Tag (1:200, CST, 2276S); anti-TNS3 (1:100, Rb33; gift from K. Clark, University of Leicester, Leicester, England, UK).
Validation	The antibodies have been validated either by the manufacturer or using RNAi silencing by the investigator team

Eukaryotic cell lines

Policy information about cell lines and Sex and Gender in Research

Cell line source(s)	Cell lines were acquired from ATCC (U2OS) or PromoCell (HUVEC)
Authentication	None of the cell lines were separately authenticated by the authors.
Mycoplasma contamination	Cell lines used in this study were regularly tested and confirmed negative for mycoplasma.
Commonly misidentified lines (See ICLAC register)	No commonly misidentified cell lines were used (ICLAC register version 13).

Plants

Seed stocks	N/A
Novel plant genotypes	N/A
Authentication	N/A
