# Supplementary material for: Adhesion-derived condensates control component availability to regulate adhesion dynamics
Source: Nat Commun. 2026 Jun 5;17:7222. doi: 10.1038/s41467-026-74001-3 (PMC13396368; doi:10.1038/s41467-026-74001-3)

**Supplementary Fig. 4A**

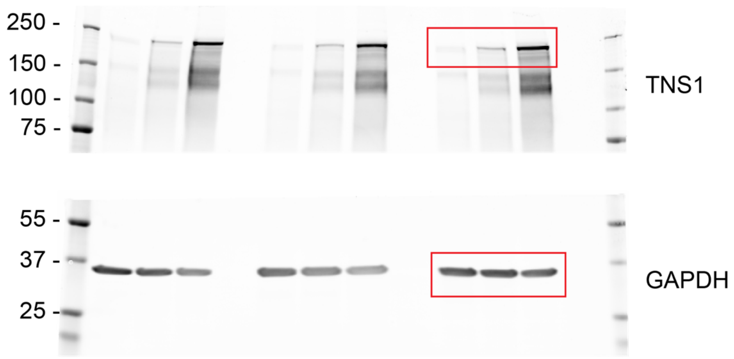

Supplement: Supplementary file 17 — Source Data [file 41467_2026_74001_MOESM17_ESM.zip › Source Data files/Uncropped blots/Supplementary Figure 4A.pdf]
