# Supplementary figures and images for: Adhesion-derived condensates control component availability to regulate adhesion dynamics
Source: Nat Commun. 2026 Jun 5;17:7222. doi: 10.1038/s41467-026-74001-3 (PMC13396368; doi:10.1038/s41467-026-74001-3)

**Fig. 3G**

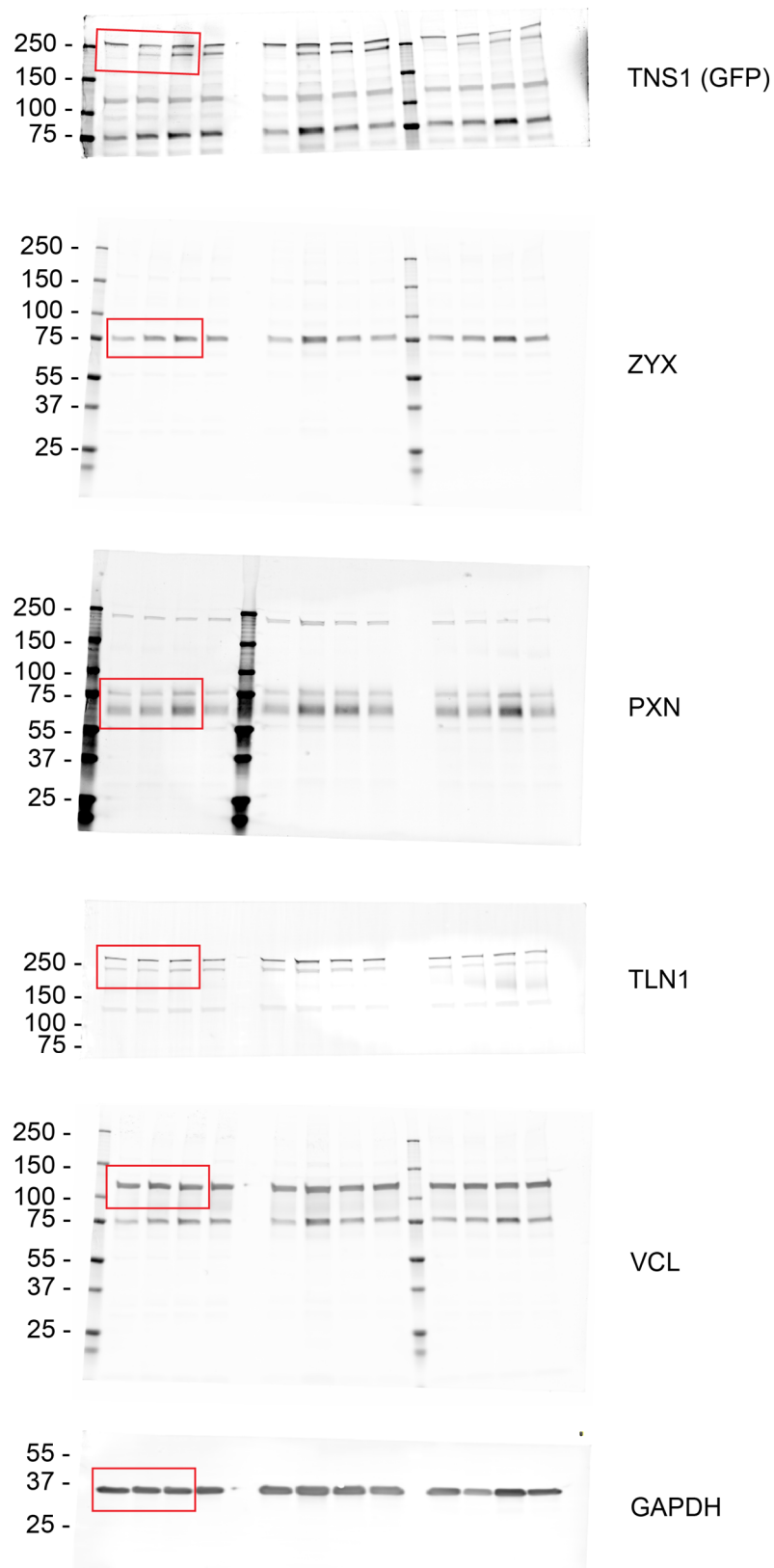

Supplement: Supplementary file 17 — Source Data [file 41467_2026_74001_MOESM17_ESM.zip › Source Data files/Uncropped blots/Figure 3G.pdf]

**Fig. 6C**

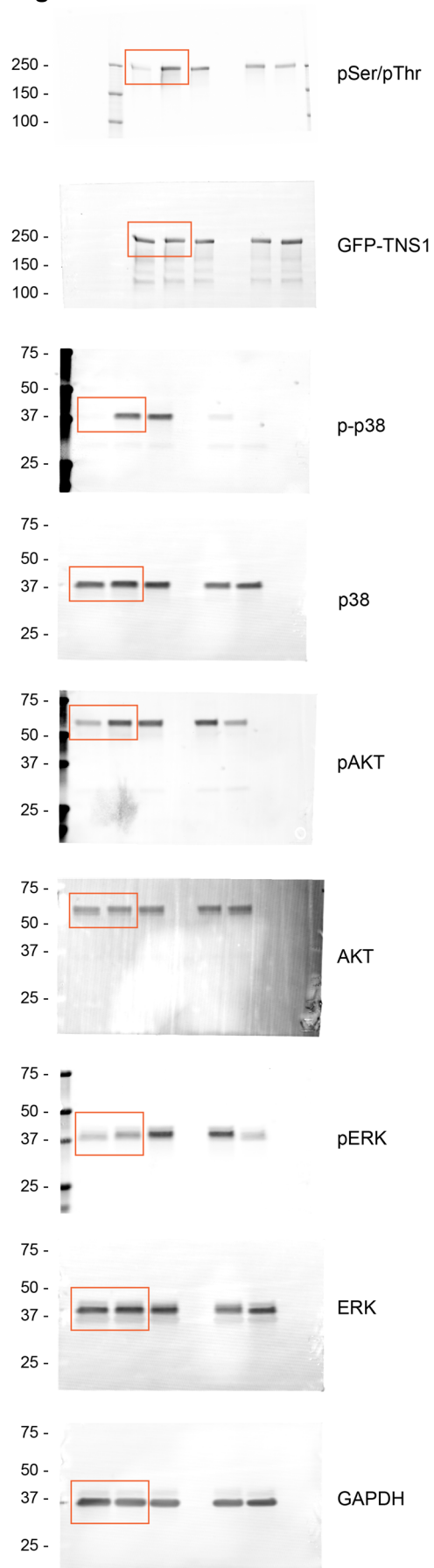

Supplement: Supplementary file 17 — Source Data [file 41467_2026_74001_MOESM17_ESM.zip › Source Data files/Uncropped blots/Figure 6C.pdf]

**Supplementary Fig. 10A**

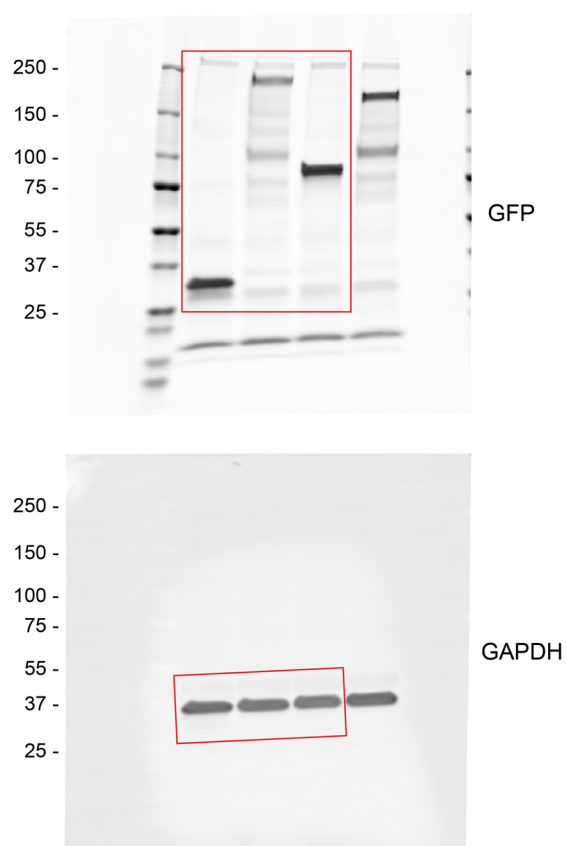

Supplement: Supplementary file 17 — Source Data [file 41467_2026_74001_MOESM17_ESM.zip › Source Data files/Uncropped blots/Supplementary Figure 10A.pdf]

**Supplementary Fig. 12D**

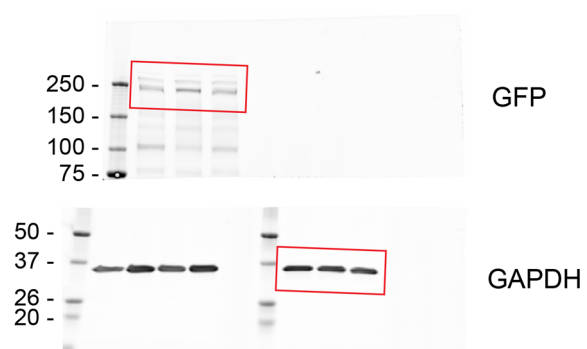

Supplement: Supplementary file 17 — Source Data [file 41467_2026_74001_MOESM17_ESM.zip › Source Data files/Uncropped blots/Supplementary Figure 12D.pdf]

**Supplementary Fig. 2B**

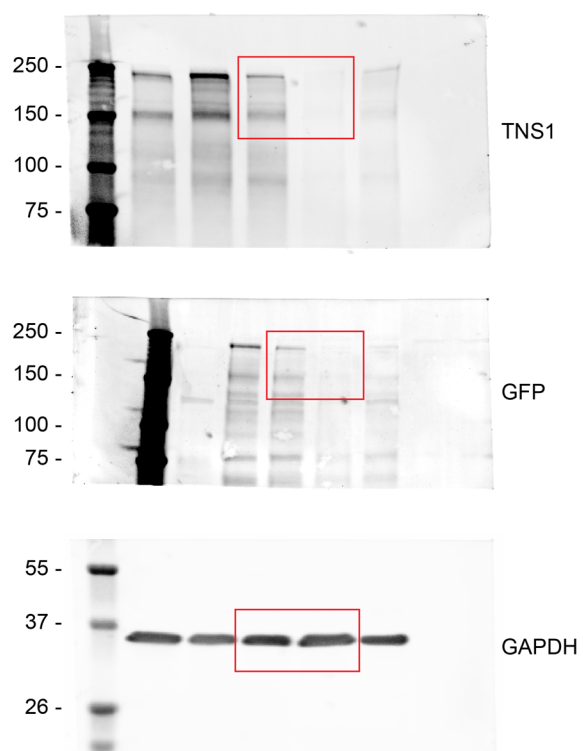

Supplement: Supplementary file 17 — Source Data [file 41467_2026_74001_MOESM17_ESM.zip › Source Data files/Uncropped blots/Supplementary Figure 2B.pdf]

**Supplementary Fig. 4C**

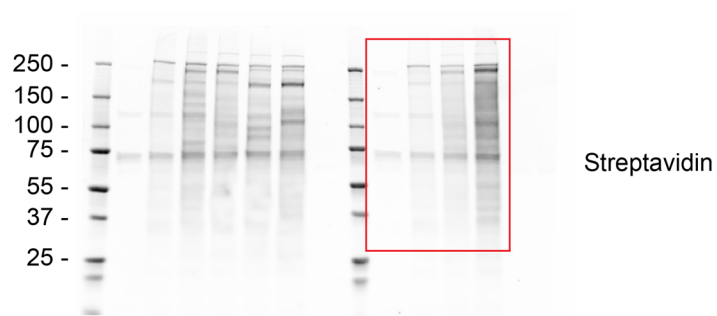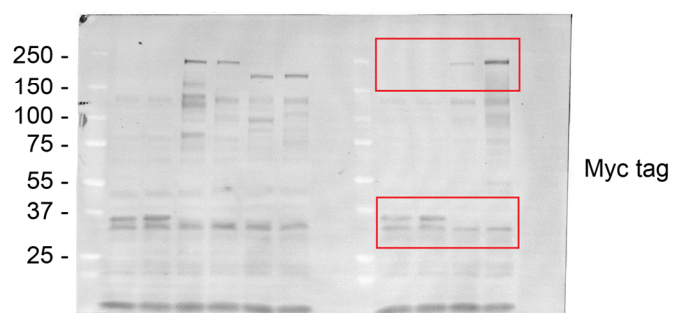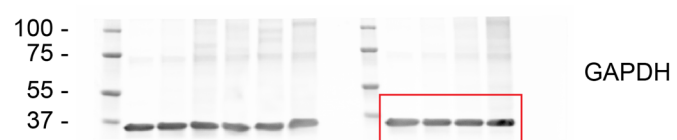

Supplement: Supplementary file 17 — Source Data [file 41467_2026_74001_MOESM17_ESM.zip › Source Data files/Uncropped blots/Supplementary Figure 4C.pdf]

**Supplementary Fig. 7B**

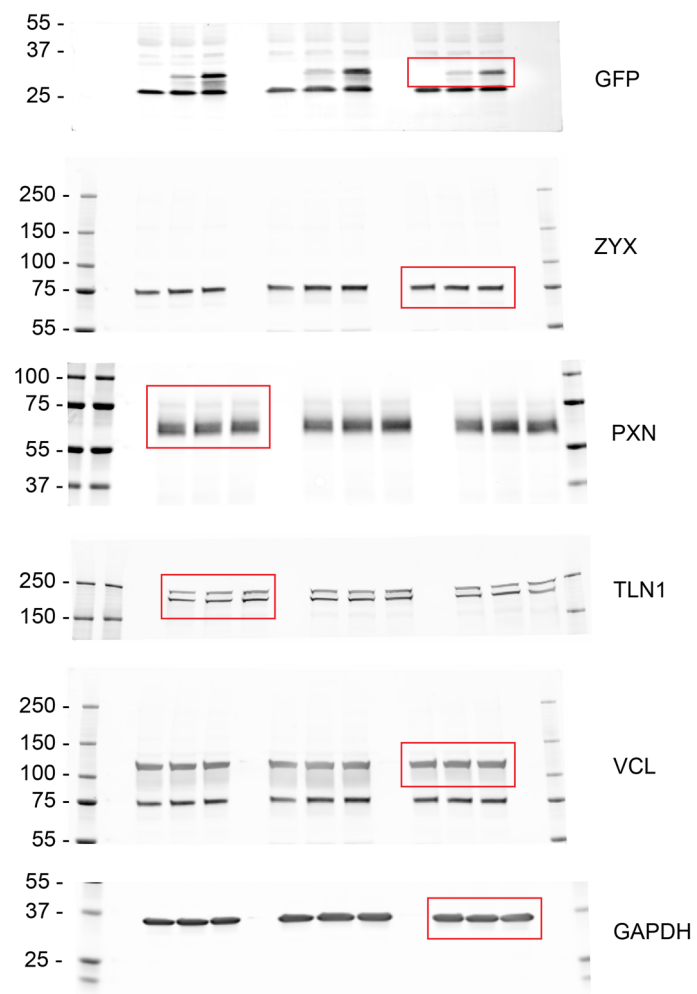

Supplement: Supplementary file 17 — Source Data [file 41467_2026_74001_MOESM17_ESM.zip › Source Data files/Uncropped blots/Supplementary Figure 7B.pdf]

**Supplementary Fig. 7D**

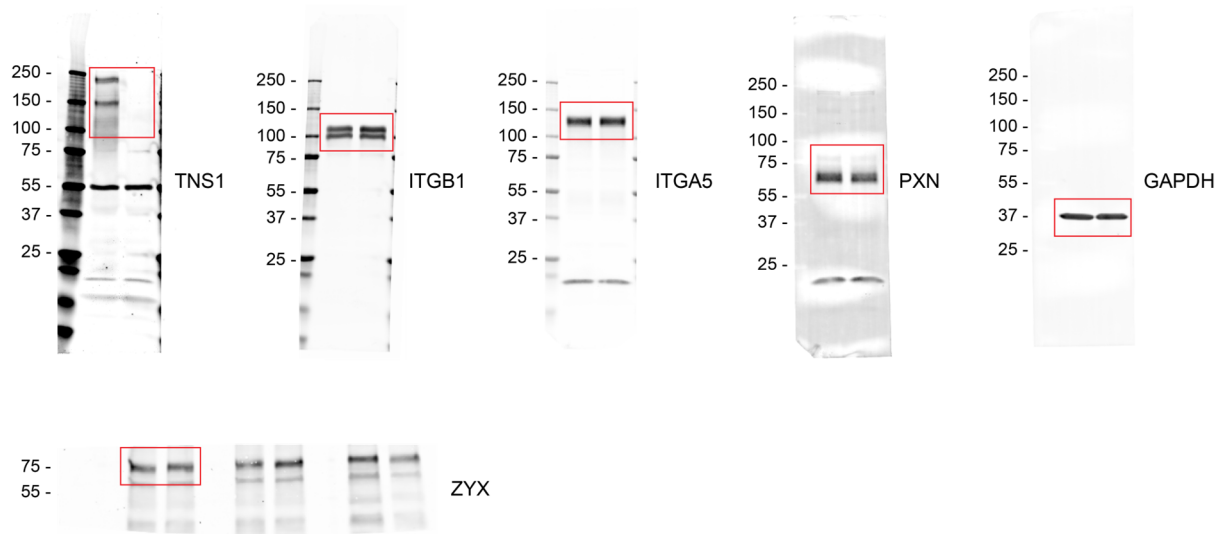

Supplement: Supplementary file 17 — Source Data [file 41467_2026_74001_MOESM17_ESM.zip › Source Data files/Uncropped blots/Supplementary Figure 7D.pdf]
