# Supplementary material for: Adhesion-derived condensates control component availability to regulate adhesion dynamics
Source: Nat Commun. 2026 Jun 5;17:7222. doi: 10.1038/s41467-026-74001-3 (PMC13396368; doi:10.1038/s41467-026-74001-3)
Supplement: Supplementary file 16 — Transparent Peer Review File [file 41467_2026_74001_MOESM16_ESM.pdf]

nature portfolio

Peer Review File

Adhesion-derived condensates control component availability to regulate adhesion dynamics

Corresponding Author: Professor Johanna Ivaska

This manuscript has been previously reviewed at another journal. This document only contains information relating to versions considered at Nature Communications.

This file contains all reviewer reports in order by version, followed by all author rebuttals in order by version.

Version 0:

Reviewer comments:

Reviewer #1

(Remarks to the Author)
The authors replied to the reviewers, but their response to Reviewer 3's main concern about TNS1 condensates in focal adhesion assembly is still not satisfying to me. There is a clear contradiction between what the authors say in their rebuttal and their main hypothesis in the manuscript. Also, the new experiments are missing important controls.

1. In their response, the authors state: "we did not suggest in our manuscript that the TNS1 condensates were directly incorporated into new FAs or fusing with nascent FAs. " However, this is directly against their main idea in the manuscript (Page 5, lines 16-18): "We next hypothesized that TNS1 condensates may function to buffer the local concentration of FA proteins." , They are also saying that the disappearance of condensates "correlated with the formation of prominent TNS1-positive adhesions" (lines 34-36). If the authors are now denying that condensates fuse with new FAs, it is very hard to understand what their claim is. They cannot drop the idea in the rebuttal while keeping the "buffering effects of TNS1 condensates" in their paper.

2. The authors added new live-cell imaging experiments. But simply showing that aggregates disappear does not prove they are joining new adhesions, which is what the text implies. To clearly show this, they need to include a negative control where no cell attachment happens (e.g., using non-tagged FN or no treatment) to see the normal disappearance rate of the condensates. Also, they need to actually measure and compare the changes in aggregate intensities over time between these conditions.

3. To properly answer Reviewer 3 about the stability of paxillin and zyxin in TNS1 KO cells, the experiment needs to match their proposed mechanism. If TNS1 really acts as a buffer, the difference between cells with and without TNS1 must be tested while the cells are actively moving, in which FAs are rapidly forming and breaking down. As the authors themselves noted, looking at these proteins in non-moving cells is not enough to show TNS1's protective role.

In conclusion, I think these experiments are necessary to clearly address Reviewer 3's concerns. The authors must either provide real evidence for their model, or completely rewrite the paper to remove the claims about the TNS1's buffering effect. But, I'm afraid that taking out the main idea will remove most of the paper's novelty.

Open Access This Peer Review File is licensed under a Creative Commons Attribution 4.0 International License, which permits use, sharing, adaptation, distribution and reproduction in any medium or format, as long as you give appropriate credit to the original author(s) and the source, provide a link to the Creative Commons license, and indicate if changes were made.
In cases where reviewers are anonymous, credit should be given to 'Anonymous Referee' and the source.
The images or other third party material in this Peer Review File are included in the article's Creative Commons license, unless indicated otherwise in a credit line to the material. If material is not included in the article's Creative Commons license and your intended use is not permitted by statutory regulation or exceeds the permitted use, you will need to obtain permission directly from the copyright holder.
To view a copy of this license, visit https://creativecommons.org/licenses/by/4.0/

Reviewer #1 (Remarks to the Author):

The authors replied to the reviewers, but their response to Reviewer 3's main concern about TNS1 condensates in focal adhesion assembly is still not satisfying to me. There is a clear contradiction between what the authors say in their rebuttal and their main hypothesis in the manuscript. Also, the new experiments are missing important controls.

We appreciate the time Reviewer 1 took to evaluate our responses to the original reviewers, which helped us better understand the concerns raised by Reviewer 3 and the apparent misunderstanding regarding our proposed mechanism of TNS1-mediated buffering of adhesion components. We apologize that we had failed to explain our model in sufficient detail. Hopefully, the remaining concerns are resolved with our textual revisions and explanations as detailed in our point-by-point responses to Reviewer 1 below.

1. In their response, the authors state: "we did not suggest in our manuscript that the TNS1 condensates were directly incorporated into new FAs or fusing with nascent FAs. " However, this is directly against their main idea in the manuscript (Page 5, lines 16-18): "We next hypothesized that TNS1 condensates may function to buffer the local concentration of FA proteins." , They are also saying that the disappearance of condensates "correlated with the formation of prominent TNS1-positive adhesions" (lines 34-36). If the authors are now denying that condensates fuse with new FAs, it is very hard to understand what their claim is. They cannot drop the idea in the rebuttal while keeping the "buffering effects of TNS1 condensates" in their paper.

We are grateful for this insightful comment as it helped us to understand the misunderstanding underpinning the initial concerns of original Reviewer 3 and now Reviewer 1. They had understood that we were implying that the "buffering effect of TNS1 condensates" equals to condensates "fusing or being directly incorporated into FAs". This had not been our narrative or our claim of how the 'buffering' works. However, in hindsight, we fully understand how we could have better explained our narrative that has been consistent throughout all the submissions both in the manuscript and the response letters. What we are implying, and provide supportive strong data for, is that TNS1 condensates **buffer local concentration of FA components acting as a reversible reservoir between FA components in the cytosol and in the condensates and thus controlling the availability and recruitment to focal adhesions**. We are not implying that condensate-sequestered proteins are directly delivered to adhesions, but are rather released to the cytosol to be subsequently recruited to adhesions. This would be in line with the prevailing view in the field of how adhesion assembly works. Work from others, including detailed single molecule studies, have demonstrated that intracellular FA components are recruited to adhesions directly from the cytoplasm (PMID: 23023225). In addition, to address the comments of both Reviewers 2 and 3, we showed that **TNS1 condensates in close proximity of newly forming adhesions disassemble**, releasing the stored adhesion components into the cytoplasmic pool, thereby locally increasing their availability to be recruited into newly forming adjacent adhesions. However, **TNS1 condensates do not directly fuse with or become incorporated into FAs as such**. We apologize if this has not been clear to the Reviewers and we have adjusted the manuscript text accordingly, explaining our proposed mechanism in more detail to avoid confusion:

"Time-lapse imaging with high temporal resolution revealed that a substantial number of GFP-TNS1 condensates at the cell periphery disassembled, thereby locally increasing the availability of the

adhesion components in the cytoplasmic pool, which correlated with the formation of prominent TNS1-positive adhesions in close proximity (Extended Data Fig. 7G, Supplementary Video 6). Importantly, we did not observe TNS1 condensates to directly fuse with newly forming FAs."

To further clarify our proposed mechanism, we have now also updated our model to emphasize that the increased integrin-ECM engagement leads to TNS1 condensate disassembly in proximity of newly forming adhesions, thereby increasing the adjacent cytoplasmic pool of adhesion proteins that can be recruited to FAs. We include the updated model with the respective figure legend below for the Reviewer's convenience:

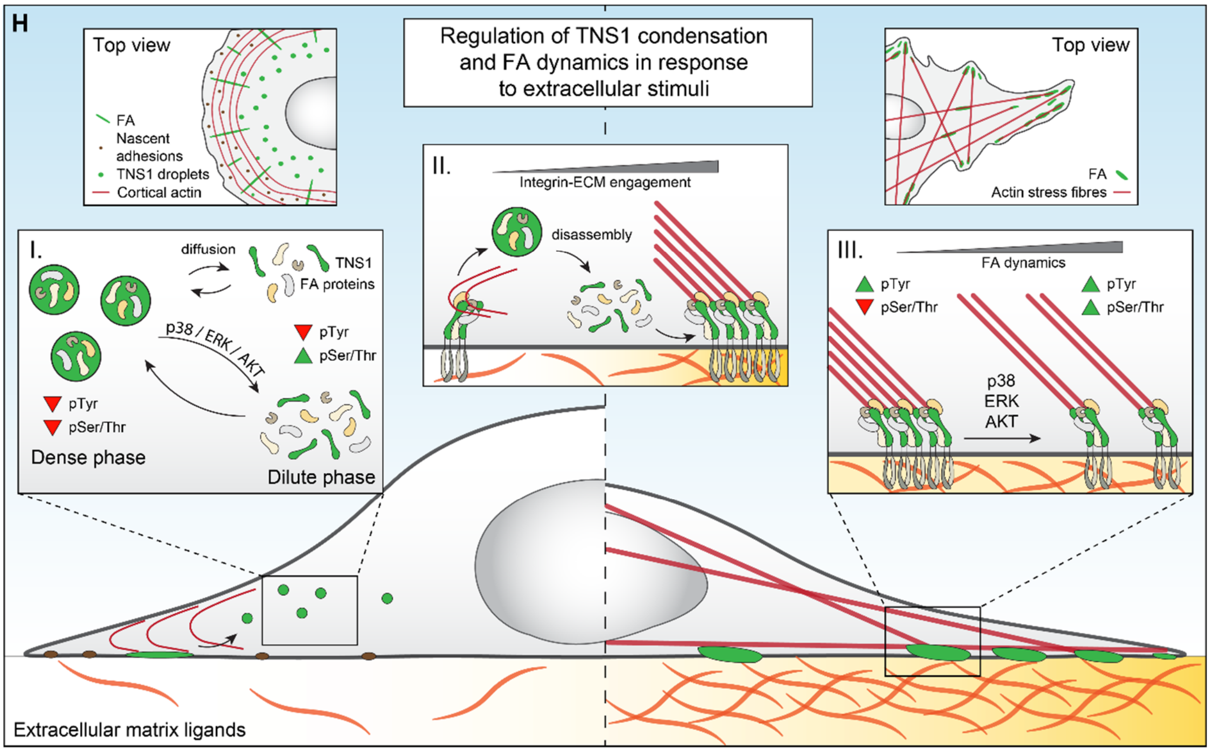

Figure legend:

Limited availability of integrin ECM ligands promotes formation of TNS1 condensates that selectively buffer the local concentration of unphosphorylated FA components. TNS1 condensates dynamically exchange material with the surrounding cytoplasm by passive diffusion or disassemble upon TNS1 phosphorylation by stress-activated kinases (I.). Increased integrin-ECM engagement leads to TNS1 condensate disassembly in proximity of newly forming adhesions, thereby increasing the adjacent cytoplasmic pool of adhesion proteins that can be recruited to FAs (II.). On ECM-rich substrates, stable adhesions are formed. Activation of stress-induced kinases leads to increased FA dynamics promoting directed cell migration (III.), likely mediated by increased phosphorylation of TNS1 in FAs and higher availability of FA components upon TNS1 condensate disassembly.

2. The authors added new live-cell imaging experiments. But simply showing that aggregates disappear does not prove they are joining new adhesions, which is what the text implies. To clearly show this, they need to include a negative control where no cell attachment happens (e.g., using non-tagged FN or no treatment) to see the normal disappearance rate of the condensates. Also, they need to actually measure and compare the changes in aggregate intensities over time between these conditions.

As explained above, it was not our intention to demonstrate that the TNS1 condensates directly fuse to newly forming adhesions, but rather that they disassemble to increase the local availability of cytoplasmic adhesion components in proximity to focal adhesions. As noted in our previous response letters, these experiments are technically challenging, and we were not able to acquire datasets large enough to support statistically robust quantitative analysis. Nevertheless, **we employed two independent approaches** (integrin activation by $MnCl_2$ and presentation of secondary ligands on functionalized surfaces), including **live cell imaging with high temporal resolution** and the use of **cell lines with endogenous TNS1 expression**, to substantiate our findings beyond a mere qualitative observation.

3. To properly answer Reviewer 3 about the stability of paxillin and zyxin in TNS1 KO cells, the experiment needs to match their proposed mechanism. If TNS1 really acts as a buffer, the difference between cells with and without TNS1 must be tested while the cells are actively moving, in which FAs are rapidly forming and breaking down. As the authors themselves noted, looking at these proteins in non-moving cells is not enough to show TNS1's protective role.

We thank the Reviewer for asking us to further clarify this point.
In response to the concerns of Reviewer 3 we noted the following: "*It is important to note that PXN and ZYX expression levels in TNS1 KO cells were assessed in **unperturbed cells** where one would not expect a high number of TNS1 condensates, but a high number of FAs where PXN and ZYX are recruited and stabilized regardless of TNS1 expression.*"
In hindsight, we regret our choice of wording and apologize for the apparent misunderstanding it has caused. In the context of our study, "unperturbed cells" do indeed represent cells that are actively moving, and in which FAs are rapidly forming and breaking down. In contrast, to fully answer the concerns of Reviewer 3, we would have to analyse the expression of paxillin and zyxin in conditions not supporting integrin-ECM adhesion, such as on PLL, where minimal number of adhesions are formed and the protective role of TNS1 condensates could be further demonstrated. We also noted in our previous response that this kind of experiment is, unfortunately, not technically feasible, since after a few hours the cells deposit enough of their own ECM on top of PLL to fully spread and form FAs that recruit and stabilize both paxillin and zyxin regardless of TNS1 expression. In addition, we do not think that the suggested experiment would provide conclusive proof on the role of TNS1 condensates in stabilizing paxillin and zyxin, since we cannot exclude the possibility that the lack of TNS1 in KO cells will be compensated by other adhesion-derived condensates, including TNS2 or TNS3. However, in our manuscript, we provide clear evidence that induction of TNS1 expression is able to acutely and specifically stabilize the protein level expression of paxillin and zyxin, which we further supported by the control experiments requested by the Reviewer 3 demonstrating that 1) induction of GFP expression is not enough to increase the expression of paxillin and zyxin, 2) the increase in paxillin and zyxin expression upon TNS1 induction is not mediated transcriptionally and 3) their expression does not increase in cells seeded on PLL when compared to other ECM substrates. To further clarify our findings, we made the following edits to the text (highlighted in yellow):

To support this notion, we first examined whether an increase in TNS1 expression could acutely stabilize the levels of FA components by their recruitment to TNS1 condensates. To test this, we induced GFP-TNS1 expression in U2OS cells using doxycycline for either 12 or 24 h and compared the expression levels of the selected proteins to those in uninduced cells. An increase in GFP-TNS1 expression led to significantly elevated levels of ZYX and PXN that are strongly recruited into TNS1 condensates. In contrast, the levels of TLN1 and VCL, which exhibit weak/limited recruitment to TNS1 condensates, were not affected by changes in GFP-TNS1 expression (Fig. 3G-H). Real-time

quantitative PCR confirmed that the increase in ZYX and PXN protein levels was not mediated by transcriptional regulation (Extended Data Figure 7A). Furthermore, expression of GFP control in U2OS cells did not affect protein expression of any of the studied FA components (Extended Data Figure 7B-C). These data suggest that elevated TNS1 levels are sufficient to support expression of ZYX and PXN proteins. However, TNS1 depletion (knock-out, KO) in U2OS cells did not alter the expression levels of ZYX and PXN in these cells (Extended Data Fig. 7D-E), implying that TNS1 is not necessary for their expression and their stability is expected to be regulated also by recruitment to adhesions and possibly to other adhesion-derived condensates.

In conclusion, I think these experiments are necessary to clearly address Reviewer 3's concerns. The authors must either provide real evidence for their model, or completely rewrite the paper to remove the claims about the TNS1's buffering effect. But, I'm afraid that taking out the main idea will remove most of the paper's novelty.

We thank Reviewer 1 again for taking the time to evaluate our rebuttal and for their valuable feedback that helped us clarify our main point and to improve our manuscript. We hope that we have addressed all the remaining concerns, allowing the publication of our study.
